# Digital and AI-Enhanced Cognitive Behavioral Therapy for Insomnia: Neurocognitive Mechanisms and Clinical Outcomes

**DOI:** 10.3390/jcm14072265

**Published:** 2025-03-26

**Authors:** Evgenia Gkintoni, Stephanos P. Vassilopoulos, Georgios Nikolaou, Basilis Boutsinas

**Affiliations:** 1 Department of Educational Sciences and Social Work, University of Patras, 26504 Patras, Greece; stephanosv@upatras.gr (S.P.V.); gnikolaou@upatras.gr (G.N.); 2 Department of Business Administration, University of Patras, 26504 Patras, Greece; vutsinas@upatras.gr

**Keywords:** CBT, sleep disorders, artificial intelligence, neurocognitive profile, insomnia, personalized therapy, AI-driven CBT, sleep improvement, mental health, digital health

## Abstract

**Background/Objectives:** This systematic review explores the integration of digital and AI-enhanced cognitive behavioral therapy (CBT) for insomnia, focusing on underlying neurocognitive mechanisms and associated clinical outcomes. Insomnia significantly impairs cognitive functioning, overall health, and quality of life. Although traditional CBT has demonstrated efficacy, its scalability and ability to deliver individualized care remain limited. Emerging AI-driven interventions—including chatbots, mobile applications, and web-based platforms—present innovative avenues for delivering more accessible and personalized insomnia treatments. **Methods:** Following PRISMA guidelines, this review synthesized findings from 78 studies published between 2004 and 2024. A systematic search was conducted across PubMed, Scopus, Web of Science, and PsycINFO. Studies were included based on predefined criteria prioritizing randomized controlled trials (RCTs) and high-quality empirical research that evaluated AI-augmented CBT interventions targeting sleep disorders, particularly insomnia. **Results:** The findings suggest that digital and AI-enhanced CBT significantly improves sleep parameters, patient adherence, satisfaction, and the personalization of therapy in alignment with individual neurocognitive profiles. Moreover, these technologies address critical limitations of conventional CBT, notably those related to access and scalability. AI-based tools appear especially promising in optimizing treatment delivery and adapting interventions to cognitive-behavioral patterns. **Conclusions:** While AI-enhanced CBT demonstrates strong potential for advancing insomnia treatment through neurocognitive personalization and broader clinical accessibility, several challenges persist. These include uncertainties surrounding long-term efficacy, practical implementation barriers, and ethical considerations. Future large-scale longitudinal research is necessary to confirm the sustained neurocognitive and behavioral benefits of digital and AI-powered CBT for insomnia.

## 1. Introduction

Sleep is a crucial physiological process for cognitive function, emotional state, and overall well-being. However, sleep disorders, notably insomnia, and the associated issues plague a vast majority of the population, causing serious public health concerns. Insomnia, marked by difficulty initiating and maintaining sleep, affects approximately 10–15% of the adult population [1]. Other sleep disorders, including obstructive sleep apnea and hypersomnia, also increase the risk of health conditions such as cardiovascular diseases, metabolic disorders, and mental illness [2,3]. Cognitive behavioral therapy for insomnia (CBT-I) is strongly positioned as the first-line non-pharmacological intervention for sleep disorders [4]. It effectively addresses maladaptive sleeping habits and cognitions underlying poor sleep. However, despite being clinically effective, CBT-I also possesses certain limitations, including restricted availability due to a shortage of trained therapists [5], insufficient individualization, making treatment less effective for individuals with unique sleeping patterns and comorbid conditions [6], and excessive variability in patient activity and adherence, affecting long-term outcomes [7].

Integrating artificial intelligence (AI) into cognitive behavioral therapy for insomnia (CBT-I) is a promising solution to address these limitations. AI-driven CBT solutions utilize machine learning, chatbots, mobile applications, and digital therapeutics to tailor treatment plans, improve user experience, and offer scalable solutions to manage sleep disorders [8,9]. These innovations pose some key questions on their efficacy, potential for personalization, longitudinal effect, and real-world adoption [10].

This systematic review evaluates and synthesizes the current evidence of using AI-based approaches in personalized CBT for insomnia and related sleep disorders. It aims to establish the impact of AI-enhanced CBT interventions on sleep quality, including sleep efficiency, latency, and general quality [11], compare the efficacy of AI-based CBT with standard digital CBT protocols and traditional therapy models [12], and explore the long-term effects of AI-enhanced CBT on sleep quality and relapse prevention [13].

The analysis also examines how AI-driven CBT personalizes treatment plans based on individual sleep patterns, behaviors, and neurocognitive profiles [14]. It provides an outline of AI’s role in dynamically adjusting CBT elements to enhance patient outcomes [15]. The other key focus is measuring how AI enhances user engagement and adherence to treatment compared to traditional digital CBT interventions [16] and investigating patient attitudes toward the usability, accessibility, and effectiveness of AI-enhanced CBT solutions [17]. Additionally, the review discusses salient benefits and limitations of AI incorporation into CBT for sleep disorders, identifies barriers and enablers influencing the introduction of AI-powered CBT in clinical practice, and considers ethical, data privacy, and technological concerns.

## 2. Literature Review

### 2.1. CBT for Insomnia

Insomnia, defined as difficulty going to sleep, staying asleep, or experiencing non-restorative or poor-quality sleep, is the most common sleep disorder. It is estimated that 30% of the world population suffers from sleep disorders at any time, and global levels of insomnia range from 6% to 48%. A specific treatment for insomnia, termed cognitive behavioral therapy, is highly recommended to treat insomnia [18,19]. The principles of CBT are designed to inform individuals about sleep and insomnia and to address cognitive, affective, and behavioral processes that maintain insomnia. The concept considers individual issues from diverse components. These challenging behaviors are difficult to overcome, including poor expectancies, cognitive arousal, poor sleep habits, and misperceptions related to sleep [20,21]. There is also a significant amount of personalization required to treat insomnia. Many difficulties and problems in treating insomnia must be addressed [22].

CBT has been tried out as an effective technique to improve insomnia patients’ psychological and behavioral capabilities. In developed countries, it has been defined as the first-choice therapy and the “gold standard” for insomnia care. Insomnia treatments based on CBT may include individual face-to-face care delivered by trained practitioners through the Internet and, more recently, through e-health programs. CBT is an effective treatment for different types of insomnia, including chronic primary insomnia or insomnia that occurs as a common comorbid condition [23,24]. Sleep-focused psycho-educational components, which include stimulus control and developing helpful attitudes towards sleep, work together to address cognitive restructuring problems. There is a comprehensive analysis of cognitive monitoring, discussion, and testing of different beliefs, psychological distress, and related insomnia. The behavioral components include experiments, relaxation strategies, physical activities to improve sleep, and reduced sleep anxiety [25,26].

To propose personalized CBT strategies, we first review the essential elements of CBT for sleep disorders. In a review focused on the components of CBT for insomnia, five key components were identified: sleep restriction, cognitive strategies, mindfulness, behavioral strategies, and psychoeducation [27,28]. However, more generally, in CBT, the key components include stimulus control, sleep hygiene education, cognitive restructuring, sleep restriction, and relapse prevention strategies. Each component and potential barriers to implementing these strategies are briefly described below. They are organized in a sequence of likely implementation, but this is typically personalized and flexible across individuals. This strategy focuses on re-personalizing where and when the bedroom is used and is particularly important for those with chronic insomnia [29,30]. This is imperative for CBT because of the plasticity of sleep and common problems with the maintenance of treatment effects when stopping sleep medications and other sleep aid medications. Understanding these components and the necessary elements for success is essential to proposing specific, personalized CBT components for people with sleep disorders and implementing an AI strategy [31,32]. Improving engagement with CBT is a critical public health initiative as patient adherence is currently low. Identifying and capitalizing on the synergistic effects of different CBT components may also advance the field by proposing novel strategies to enhance treatment by targeting multiple sleep regulatory systems. The proposal of these new strategies may also substantively advance scientific knowledge about sleep regulation [8,33,34].

### 2.2. AI in Healthcare

AI is on the rise in the healthcare industry. Indeed, this technology is widely believed to revolutionize the early diagnosis and treatment of many diseases, significantly improving the quality of care and patient outcomes [35,36,37]. AI relies on a mixture of technologies such as data analytics, diagnostic capabilities, and other computer applications able to interpret medical data, all covered by the umbrella term of cognitive computing. AI in healthcare can range from data analytics that uncover patterns in large datasets to natural language processing technologies that can identify patterns in unstructured data and build knowledge [38,39]. Machine learning algorithms can determine patterns among data and autonomously modify their calculations without human intervention. Deep learning is a subset of machine learning that processes vast amounts of data and identifies patterns. Recent studies have demonstrated the potential of adaptive ensemble deep learning frameworks and information fusion strategies for enhancing predictive accuracy in high-stakes environments such as pandemic detection [40,41]. These approaches, including dynamic model selection and optimized integration of neural networks, can offer a blueprint for improving AI-driven CBT systems by enabling adaptive learning and more precise personalization of therapy [42,43].

Day-to-day applications of AI in healthcare include leveraging predictive analytics to forecast future clinical events, creating individualized care strategies, improving patient engagement and satisfaction, and reducing high costs typically associated with chronic diseases [44,45]. AI-powered triage systems can predict where and when flu outbreaks might begin; intelligent computer-aided diagnosis can identify cancers in earlier stages than ever before; data analytics can help to reduce patient no-show rates and lengths of stay. However, to ensure the complete protection of patient privacy and to adhere to ethical and consent considerations, some policies help limit the use of AI in healthcare. AI’s potential and public expectations in healthcare are high [46]. Still, innovation in regulation and public engagement is needed to ensure that technology delivers better and fairer outcomes for citizens. AI systems are also increasingly used for mental health and sleep disorder care [47,48].

In the domain of sleep disorders, AI can have multiple applications. AI algorithms are proposed for automatically analyzing polysomnography or actigraphy recordings, potentially enabling automatic diagnosis and sub-typing of sleep disturbances [49,50]. Tools also aim to automate differential diagnosis, predict treatment response, and extract circadian parameters from long-term data. AI is further harnessed for therapeutic purposes such as sleep disturbance self-evaluation, chatbot insomnia treatments, and the development of regenerative soundscapes [51,52]. Furthermore, AI solutions are proposed for monitoring sleep or the impact of sleep disorders on comorbid diseases. The personalization of cognitive behavioral therapy is explored primarily using recommender systems, reflecting the state of the implementation of automated solutions [53,54].

AI can also lead to more personalized therapeutic strategies. For example, a computational model is proposed for tailoring the timing and rate of dose reduction in patients with nightmares. An ensemble of machine learning algorithms is developed to predict treatment initiation based on psychometric data. Data-driven case studies focused on using machine learning for extracting and analyzing subjective, actigraphic, and/or polysomnographic data from patients with a circadian sleep disorder and tracking insomnia symptoms are reported [55]. Many case studies, tools, and AI applications in sleep disorders and their diagnosis and tailored therapy range demonstrate the interest in exploiting AI to enhance thresholds in an outpatient care context. Integrating cognitive behavioral therapy for insomnia within digital, AI-based patient care most probably supports building resilience to crises concerning both outpatient care and more specialized settings. Many still require validation in more extensive studies or case series. Decentralized healthcare delivery may benefit from such innovations. However, the pivotal role of therapists should remain paramount in applying AI to patient care. Precise needs and ample experiences demonstrate the advantage of blended treatments and regular, personal contact with patients [56,57,58].

### 2.3. Personalized Medicine in Sleep Disorders

In personalized medicine, optimal treatment strategies are developed according to the individual biopsychosocial profiles of patients in contrast with universal treatments applied to all patients with a particular disorder. Patients with sleep disorders can manifest different signs and symptoms related to the heterogeneity of such disorders. They might have other genetic, environmental, and lifestyle factors relevant to the trajectories of these disorders [59,60,61]. Consequently, the effectiveness of standardized interventions will vary in different patients based on the specific cause of the disorder. One of the primary goals of personalized medicine in sleep disorders is to diagnose these factors and customize treatment to enhance effectiveness and reduce the time of therapeutic response. Tailored interventions would not contain unnecessary treatments that are not helpful to a particular patient and reduce costs. Following up on the development of customized treatments is essential, given the possibility of adverse events [62,63,64].

Some examples of tools to facilitate personalized management in people with sleep disorders have been developed in the last 10 years. A brief review of the tailored treatments for insomnia and obstructive sleep apnea has been published, including personality inventory or actigraphy as tools for subsequent treatments. Other promising individualized sleep disorder therapy approaches could follow the precision psychiatry approach, which combines clinical scale measures with neuroimaging and demographic data [65,66]. This method did not use machine learning but included deep investigations of the pathophysiology of different sleep–wake disorders and how they relate specifically to the disease burden of low-income countries. Critical assessment of the featured regimes consists of the generally inaccessible cost yet compatible scalability and the gaps in evidence surrounding long-term efficacy and sustainability in diverse community settings. Furthermore, it is noteworthy that the cost and difficulty of developing an app compels a one-size-fits-all model, which could over-target the leading causes and look to eliminate rather than manage and treat the broader range of treatment targets that emerge from studies [67,68,69].

Moreover, sleep disorders are more prevalent in patients with mental disorders than in the general population and are often presenting symptoms in psychiatric consultations. The suffering and impairment they cause are important therapeutic objectives of greater relevance to the patient and healthcare professional. However, clinical practice guidelines on sleep–wake disorders have focused on treating medical comorbidities or aspects of the quality of life, neglecting a significant part of the treatment. The use of clinical decision instruments that integrate genetic, environmental, and other factors can help mental health professionals in the clinical approach to the sleep–wake cycle, adopting both prevention and treatment focused on these disorders [70,71]. Mental health professionals can also lead in expanding precision psychiatry programs to cover the entire sleep–wake cycle, involving more clinical scientists and bioinformaticians to profile neurobiological rhythms at the system level for an extensive range of health and patient groups. To the best of our knowledge, research identifying the main traits of sleep–wake function was aimed at giving an operational impact in psychiatry practice rather than in neurological diseases, lifestyle, and all-cause mortality, which included conference abstracts, protocols, and apps focused on sleep duration, continuity, and the activity of wearable devices, especially during sleep [72,73,74].

### 2.4. Integration of AI and CBT in Sleep Disorder Management

Sleep disturbances appear in about 20% to 30% of adults. They may start or continue during old age and cause personal suffering, a reduced quality of life, physical and mental health problems, and an excess of expenditures in healthcare systems. CBT is the first-line treatment for insomnia, both for primary and comorbid forms. It induces long-term improvement in 70% to 80% of patients and has an effect size for reducing sleep latency like that of prescription drugs. However, it is complex, takes place in specialized multidisciplinary sleep centers that are not available everywhere, and is expensive. Moreover, differences among individuals in symptom generation demand a personalization of approaches. Artificial intelligence may help provide health professionals with data-driven insights on pathophysiology and make contact tracing, diagnosis, or treatment monitoring more accurate. AI and CBT together may constitute a comprehensive approach to managing sleep disorders [75,76].

AI-driven methods for CBT can have two directions. They can offer data support to therapists, providing relevant information about the patient’s health condition and helping to predict outcomes, making it especially useful for treatment personalization. CBT can also rely on AI’s continuous analysis of patient-related data [77,78]. This way, trends and possible critical situations can be monitored by AI, which will warn health professionals of the need for immediate therapy adjustment. The future of modern healthcare, which is patient-centered and more focused on preventing chronic, sleep-related health problems, is going in this direction [79,80]. It requires a multidisciplinary team that includes computer science and medicine experts. Integrating AI with CBT may revolutionize the management of sleep disorders, increasing the percentage of positive outcomes [81,82].

In treating specific areas of sleep, AI can be understood as a model of CBT delivery rather than a treatment directly targeting sleep itself. Given sleep disorders’ complex, multimorbid nature, effective interventions may require mobilizing assistance from various professional disciplines. This view offers several benefits that justify human clinicians’ ability to blend professional experience with the high-performing statistical associations that constitute AI to maximize individual effectiveness. Some potential benefits of integrating AI within sleep CBT include enhanced treatment personalization via patient scale-up and cross-patient risk stratification. Effective data and sensor-driven fatigue guarantees that patients engage actively in many treatment trials, allowing real-time measurement of the effective variables, and might promote the provision of decentralized sleep care solutions, thereby reducing waiting lists and administrative costs [83,84].

### 2.5. Research Questions

The increasing prevalence of sleep disorders has led to the adoption of various therapeutic approaches, including cognitive behavioral therapy for insomnia (CBT-I). However, the traditional delivery of CBT faces challenges, such as accessibility and scalability. In recent years, digital platforms have provided a promising solution to these barriers, allowing for remote delivery of CBT via digital cognitive behavioral therapy (dCBT). More recently, AI has emerged as a powerful tool that could enhance the effectiveness and personalization of CBT interventions.

Personalized CBT can tailor treatments to each patient’s unique needs, particularly when combined with AI-driven systems based on user data that can adapt in real time. AI can facilitate dynamic interaction, enhance user engagement, and improve treatment adherence, offering a more customized therapeutic experience. However, despite the growing body of research on AI-enhanced and digital CBT, questions remain regarding the efficacy, personalization, and practical implementation of these interventions for individuals with sleep disorders.

In this systematic review, we aim to address several key research questions that will shed light on the effectiveness of digital and AI-driven CBT for sleep disorders, focusing on personalization, engagement, and long-term outcomes. The following research questions guide our analysis of the current literature:

▪ [RQ1] How effective are digital CBT interventions in improving sleep outcomes for individuals with sleep disorders?
▪ [RQ2] What are the key benefits and challenges of integrating AI into CBT for treating sleep disorders?
▪ [RQ3] To what extent do AI-driven CBT interventions personalize treatment plans based on individual sleep patterns and behaviors?
▪ [RQ4] How does the use of AI in CBT improve user engagement and treatment adherence compared to traditional digital CBT interventions?
▪ [RQ5] What are the key differences in efficacy between AI-driven CBT and standard digital CBT for sleep disorders?
▪ [RQ6] What are the common barriers and facilitators to implementing AI-driven CBT for sleep disorders in clinical practice?
▪ [RQ7] What are the long-term effects of digital and AI-driven CBT interventions on sleep quality and relapse prevention?
▪ [RQ8] How do patients perceive the usability and effectiveness of AI-enhanced CBT interventions for treating sleep disorders?

## 3. Materials and Methods

This systematic review aims to explore the integration of AI into personalized CBT for treating sleep disorders. Sleep disorders, such as insomnia and sleep apnea, significantly impact health and quality of life, with conventional CBT showing effectiveness but limited scalability and personalization. This review investigates how AI-enhanced tools, including chatbots, mobile applications, and web-based platforms, contribute to delivering more accessible, scalable, and tailored CBT interventions. The review also evaluates the efficacy, feasibility, and patient adherence of these AI-driven approaches compared to traditional methods. Key objectives include identifying methods of personalization within CBT using AI, assessing the impacts of AI-enhanced CBT on sleep outcomes, and providing recommendations for integrating AI into CBT frameworks.

### 3.1. Analytical Search Process

This systematic review follows the PRISMA (Preferred Reporting Items for Systematic Reviews and Meta-Analyses) guidelines to ensure transparency and replicability in the synthesis of evidence [85]. A protocol outlining the objectives, eligibility criteria, information sources, and analysis methods was registered on Open Science Framework (https://osf.io/mc4jk; registration DOI: 10.17605/OSF.IO/47XK2) [86].

The analytical search began with identifying 478 records through database searches conducted across PubMed, Scopus, Web of Science, Google Scholar, and PsycINFO. Before the screening, 294 records were removed, comprising the following:

- 238 duplicate records;
- 16 records due to language restrictions;
- 10 records published before 2004;
- 30 records with non-relevant titles.

This left 184 records for screening. During the screening phase, the titles and abstracts of these records were reviewed, resulting in the exclusion of the following:

- 36 records for being irrelevant to the topic;
- 44 non-RCT articles, such as commentaries, opinion pieces, and reviews.

This screening process left 104 reports to be retrieved for further assessment. Out of these, 5 reports could not be retrieved due to difficulties in accessing the full text. The remaining 99 reports were assessed for eligibility, where

- 11 were excluded for insufficient methodological detail;
- 15 were excluded for lacking direct relevance to the research question.

**Figure 1:**
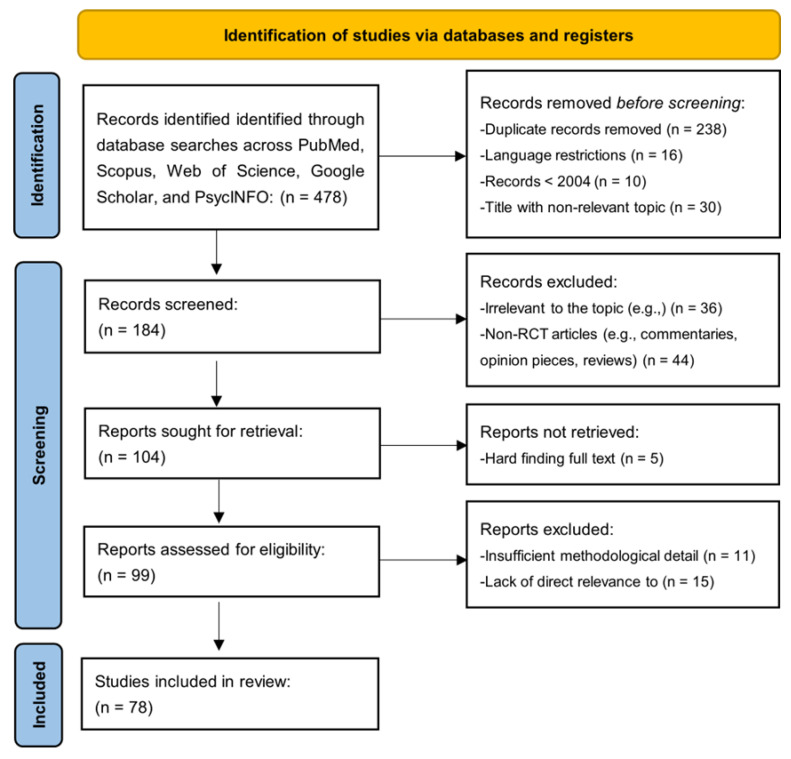
Flowchart of PRISMA methodology.

Ultimately, 78 studies were included in the final review. This process reflects a systematic and rigorous approach to screening and selecting studies, ensuring transparency and adherence to PRISMA guidelines. The included studies form the basis for the synthesis and further analysis (Figure 1).

### 3.2. Search Strategy

The search strategy for this systematic review was designed to capture studies addressing personalized cognitive behavioral therapy (CBT) for sleep disorders, focusing on integrating artificial intelligence (AI), digital tools, and tailored therapeutic approaches. The search terms included “Cognitive Behavioral Therapy” OR “CBT” OR “Personalized CBT” OR “AI-driven CBT”, “Digital Tools” OR “Artificial Intelligence” OR “Personalized Medicine”, “Sleep Disorders” OR “Insomnia” OR “Sleep Quality”, and “Randomized Controlled Trial” OR “RCT”. The following search string used was:

*(“Cognitive Behavioral Therapy” OR “CBT” OR “Personalized CBT” OR “AI-driven CBT”) AND (“Digital Tools” OR “Artificial Intelligence” OR “Personalized Medicine”) AND (“Sleep Disorders” OR “Insomnia” OR “Sleep Quality”) AND (“Randomized Controlled Trial” OR “RCT”).*

Search terms and strategies were customized for each database. In PubMed/Medline, MeSH terms such as “Cognitive Behavioral Therapy” [MeSH], “Sleep Disorders” [MeSH], and “Randomized Controlled Trial” [Publication Type] were used. PsycINFO searches were refined by limiting RCTs and incorporating therapy- and sleep-specific terms. Scopus and Web of Science utilized the “Topic” field with filters for RCTs and terms related to personalized medicine, digital tools, and AI.

Filters restricted results to studies published from 2004 to 2024, written in English, and focused on randomized controlled trials investigating digital tools, AI, or personalized approaches within CBT frameworks for sleep disorders (Table 1). This approach ensured a focused and comprehensive selection of high-quality studies relevant to the review.

### 3.3. Inclusion and Exclusion Criteria

The inclusion and exclusion criteria from the manuscript are as follows:

Inclusion Criteria

- Studies investigating the efficacy, feasibility, or applicability of personalized cognitive behavioral therapy (CBT) for sleep disorders.
- Randomized Controlled Trials (RCTs).
- Studies employing digital tools, artificial intelligence (AI), or personalized approaches within CBT interventions.
- Articles published in peer-reviewed journals after 2004.
- Research written in English.
- Full-text availability for comprehensive review.

Exclusion Criteria

- Studies not focused on CBT or related interventions for sleep disorders.
- Non-empirical papers such as reviews, commentaries, and opinion pieces.
- Articles published in languages other than English.
- Research focused on populations or disorders outside the scope of sleep disorder treatment.
- Insufficient methodological detail or lack of direct relevance to personalized or AI-driven CBT for sleep disorders.
- Studies were published before 2004.

These criteria were applied systematically to filter the literature, ensuring relevance, methodological rigor, and alignment with the review’s focus on personalized and AI-enhanced CBT for sleep disorders.

### 3.4. Risk of Bias Assessment

The risk of bias for the 78 included studies was assessed using the Cochrane Risk of Bias Tool. The following domains were evaluated:

1. Selection Bias (random sequence generation and allocation concealment):
  ▪ Low Risk: Most studies described adequate randomization methods and allocation concealment.
  ▪ Unclear Risk: Several studies lacked detailed descriptions of randomization or allocation procedures.
2. Performance Bias (blinding of participants and personnel):
  ▪ Moderate to High Risk: Blinding was inconsistently reported, especially for digital tools studies, where blinding is inherently challenging.
3. Detection Bias (blinding of outcome assessors):
  ▪ Low Risk: Most studies used objective measures (e.g., validated scales like ISI and PSQI) for sleep outcomes, reducing detection bias. However, some studies did not explicitly mention blinding of assessors.
4. Attrition Bias (incomplete outcome data):
  ▪ Moderate Risk: High dropout rates were observed in studies involving long interventions or follow-up periods, though many employed intention-to-treat analyses.
5. Reporting Bias (selective reporting):
  ▪ Low Risk: Most studies reported primary and secondary outcomes as stated in their protocols, though a few omitted exploratory analyses.
6. Other Bias (funding and conflicts of interest):
  ▪ Moderate Risk: Some studies involving industry-funded digital tools or AI platforms lacked transparency regarding potential conflicts of interest.

Two independent reviewers evaluated each study to ensure the reliability and consistency of the risk of bias assessment. Any disagreements in risk assessments were discussed and resolved by consensus. In cases where a consensus could not be reached, a third senior reviewer was consulted to provide an independent judgment. This process ensured a rigorous and transparent evaluation. The included studies exhibited a low-to-moderate bias risk, ensuring the synthesized findings’ reliability. However, inconsistencies in reporting methods (e.g., blinding and attrition) and potential funding biases warrant cautious interpretation of results.

**Figure 2:**
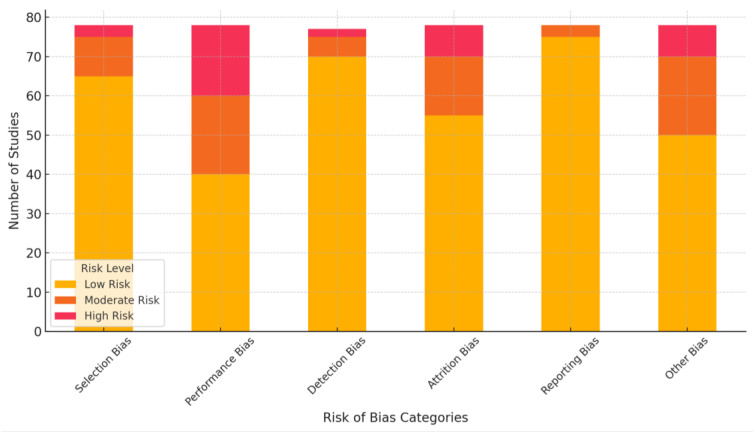
Risk of bias assessment across 78 studies.

The visualization above (Figure 2) illustrates the distribution of risk levels across the six assessed domains for the 78 studies. Each bar represents a risk category divided into low, moderate, and high-risk assessments. This clearly shows where the studies demonstrate robust methodology and where biases may influence the findings.

## 4. Results

The results of this systematic review provide a comprehensive analysis of the integration of AI into personalized CBT for treating insomnia and related disorders. The included studies evaluated the efficacy, feasibility, and scalability of AI-enhanced CBT interventions compared to traditional and digital-only methods. This section synthesizes findings from 78 studies that addressed various dimensions of AI-driven CBT, including personalized therapy delivery, patient adherence, and improvement in sleep-related outcomes.

**Figure 3:**
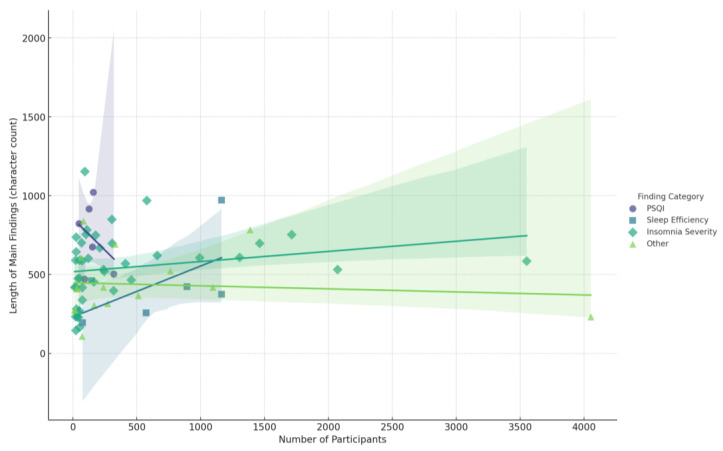
Scatter plot of study findings grouped by category with regression analysis.

The scatter plot below (Figure 3) illustrates the relationship between the number of participants in each study and the length of the main findings (character count), grouped by categorized themes, including “PSQI”, “Insomnia Severity”, “Sleep Efficiency”, “Sleep Disturbance”, and “Other”. Each category is color-coded and represented with distinct markers. Including regression lines highlights trends within each category, offering insights into how sample size may influence the depth and detail of reported findings. Studies focusing on “PSQI” and “Sleep Efficiency” appear to have stronger correlations between sample size and finding length, suggesting more detailed reporting in studies with larger participant groups.

The results are organized to address specific research questions, exploring the impact of digital and AI-enhanced CBT on sleep-related outcomes, the mechanisms driving their success, and the challenges faced during their implementation. These findings highlight the efficacy of these interventions and offer valuable insights into how AI technologies are shaping the future of sleep disorder treatment. The subsequent sections detail these findings, categorized by the research questions that guided this review.

### 4.1. [RQ1] How Effective Are Digital CBT Interventions in Improving Sleep Outcomes for Individuals with Sleep Disorders?

Digital CBT-I has emerged as a strong alternative, using the reach and convenience afforded by technology in deploying core CBT-I principles through various forms of digital platforms, including but not limited to Web-based programs, mobile applications, and other novel tools. The current comprehensive research question has canvassed wide-ranging evidence for the efficacy of dCBT-I in improving sleep outcomes across a wide range of populations suffering from various sleeping disorders. It had, therefore, aimed at the in-depth understanding of the effects from many studies, focusing on the key sleep parameters, wider benefits of dCBT-I, and efficacy across different populations and settings.

#### 4.1.1. Significant Improvements in Sleep Efficiency and Insomnia Severity: Foundational Evidence

The foundational evidence to underpin the efficacy of dCBT-I is sound and consistently evidences significant increases in sleep efficiency, along with associated reductions in insomnia symptomatology. A recent example constitutes the study’s finding [96] in which sleep efficiency increased by 34% for women and 26% for men after a seven-week digital CBT-I program. It also documented that 55% of those with subthreshold insomnia and 33% with clinical insomnia reached remission, extending the clinically significant effects of the dCBT-I. An online CBT-I intervention also improved sleep efficiency and latency and showed more modest gains in total sleep time, as documented by the study [89]. These findings are further confirmed by the study [93], which presented the dCBT group with a markedly higher SCI score, having a large Cohen’s d of 1.10, showing a considerable size effect and significant change in sleep quality.

These first studies laid the groundwork for further research, including the seminal contribution of the study [146], which, besides documenting significant improvements in insomnia severity, sleep-onset latency, and wake-after-sleep onset, demonstrated the durability of these treatment effects at a 1-year follow-up. This is an important finding because it suggests that the benefits of dCBT-I are not transient but can be maintained over time. Further, the study [115] added to the literature on the delivery of dCBT-I in demonstrating, via a mobile phone app, significant gains in insomnia severity (d = −0.66) and sleep efficiency (d = 0.71), thus proving how different digital modalities can be used versatilely and with efficiency. Based on these results, the study [121] explored the use of digital CBT-I among pregnant women, a population particularly vulnerable to sleep disturbances. The study had significant reductions in ISI and PSQI scores, thus showing the potential of dCBT-I in improving sleep during pregnancy, a period when pregnant women are usually faced with serious sleeping problems.

Further strengthening the evidence base, the authors in in the study [162] conducted a comprehensive meta-analysis of Internet-delivered CBT for insomnia (eCBT-I). Significant improvements were found in sleep parameters: insomnia severity, sleep efficiency, subjective sleep quality, wake-after-sleep onset, sleep-onset latency, total sleep time, and the number of nocturnal awakenings. Notably, the effect sizes of this meta-analysis were no different from those of face-to-face CBT-I studies, suggesting that dCBT-I can be as effective as conventional therapy. Researchers in their study [125] also widened the scope of dCBT-I’s influence by showing significant reductions in self-reported cognitive impairment while improving sleep efficiency, cognitive failures, fatigue, sleepiness, depression, and anxiety. This study underlines that the benefits coming from dCBT-I are very diverse, beyond the field of sleep improvements alone, including even more significant aspects of health. Similarly, the study [137] showed that a tailored app on a smartphone significantly reduced insomnia severity and social disabilities. In contrast, the study [101] recorded how dCBT significantly improved both efficiency and quality of sleep, reducing the symptoms of insomnia, anxiety, and depression among the adolescent population facing serious problems with sleep. Continuing the exploration of dCBT-I’s efficacy, the study [151] conducted a meta-analysis confirming that CBTi significantly improved global sleep measures post-treatment, with sustained improvements at follow-up up to 12 months.

These were further supported by the study [111], which, in turn, reported significant reductions in insomnia symptoms, g = 1.11, after an unguided iCBT-I program. A more recent study [123] showed that this IoT device that delivered CBT-I directives significantly improved the ISI by 62%, thus showing great potential for integrating innovative technology with dCBT-I. Also, the study [90] added to this evidence by reporting a clinically significant decrease of 7.42 points on the ISI, with considerable improvements also in objective sleep measures such as WASO and SE. Evidence from these cumulative studies presents a powerful argument for understanding how dCBT-I enhances sleep efficiency and reduces insomnia severity.

Each of these studies adds more to the picture with its population and methodology. Thus, this robust body of research points out that dCBT-I is effective and flexible in providing benefits from various digital platforms for diverse people with sleep disorders. The convergence of results across these studies provides a sound basis for further development and implementation of dCBT-I as a much-needed treatment option for people with sleep disorders. The benefits of dCBT-I regarding sleep parameters and its wider ramifications for mental health and general well-being must be considered in the future.

#### 4.1.2. Expanding the Scope: Broader Benefits and Long-Term Impact

Although sleep efficiency and insomnia severity improvements are central to the overall effectiveness of dCBT-I, its benefits extend far beyond these core sleep parameters to reach many other aspects of life. The study provides evidence for this broader impact [106] since dCBT was associated with significant improvements in sleep-related quality of life and reduced insomnia symptoms, which, in turn, mediated improved functional health and psychological well-being. This addresses the immediate symptoms of insomnia and subjects’ overall quality of life. This again points to culturally sensitive interventions. Furthermore, the study [163] explored how a culturally adapted dCBT-I significantly decreased ISI scores compared to sleep education alone within the Chinese population, which is illustrative of tailoring an intervention to its specific cultural context for maximum effectiveness.

As noted by the study [164], digital health technologies have the potential to make CBT-I more scalable and cost-effective without compromising quality in patient outcomes. This is because there is a shortage of trained CBT-I therapists and an increasing demand for accessible mental health services. Also, the study [142] furthered this line of inquiry by reporting clinically significant improvements in insomnia severity, overall sleep quality, and disruptive nocturnal disturbances among military service members and veterans—a well-documented population to suffer from high rates of sleep disorders, as well as other mental health challenges. Apart from populations, researchers also investigated the effectiveness of dCBT-I on comorbid disorders. Also, researchers in the study [119] investigated the efficacy of group-delivered CBT-I in adults with ADHD. They demonstrated that this kind of treatment significantly reduced insomnia severity and thus may be effective even if delivered in groups and for subjects with co-occurring disorders. This has considerable implications for delivering dCBT-I in various settings and for diverse populations. Furthermore, researchers in the study [98] have demonstrated the long-term efficacy of dCBT-I in building resilience during the COVID-19 pandemic. Subjects who had previously received dCBT-I reported less insomnia, stress, and depression during this challenging period compared with those receiving sleep education, which might suggest that dCBT-I can result in long-term benefits.

Moreover, the study [145] explored the value added to TD-CBTI by embedding personalized telephone sessions. The integrated approach conferred extra clinical benefits on the patients, including increased chances of remission based on sleep efficiency and reduced use of medication to sleep. Hence, such findings suggest that digital interventions might be combined with human support to achieve better outcomes. Another critical investigation on the impact that dCBT-I has on mental health issues was provided by the study [99], which considered dCBT-I related to the prevention of depression within 1 year and resulted in insomnia subsequently. This effect is deemed impressive because, as stated herein, dCBT-I can prevent depressive disorders—a widely prevailing factor in insomnia patients. Similarly, the study [154] reported that patients receiving the digital sleep intervention Sleepio had significantly better outcomes for core clinical, including PHQ-9 and GAD-7, compared to controls, thus providing further support for the broader mental health benefits of dCBT-I. The study [128] also added to the comparative effectiveness literature by reporting the superiority of dCBT-I to medication therapy at the 6-month follow-up. Combining dCBT-I with medication may achieve long-term benefits in improving sleep quality and could suggest some synergistic effects. Finally, the study [134] reported significant reductions in insomnia severity, anxiety, and depression with a prescription digital therapeutic delivering CBT for insomnia (Somryst^®^, Nox Health, GA, USA), further solidifying the evidence for dCBT-I’s positive impact on mental health outcomes.

#### 4.1.3. Tailoring Interventions: Specific Populations, Settings, and Future Directions

The versatility and adaptability of dCBT-I are further evidenced by its effectiveness across various populations and settings. For example, the study [161] reported that a smartphone-based CBT-I app significantly improved the Athens Insomnia Score compared to a sham app, demonstrating how effective mobile-based interventions could be. This finding is relevant to the present day, given the ubiquity of smartphones as a convenient delivery platform for interventions in mental health. Focusing on the specific challenges of shift work, the study [138] showed that personalized light therapy significantly improved insomnia symptoms and sleepiness among night shift workers. Similarly, researchers in a study [118] investigated the potential of Internet-based CBT for shift work sleep disorder to improve sleep time and overall well-being. These studies describe how CBT-I principles can be adapted to meet specific sleep challenges.

In the comparative effectiveness study [92], guided Internet-delivered CBT was equally effective as group-delivered CBT for insomnia, yielding large effect sizes. This is significant in pointing toward the possibility that dCBT-I is as effective as group therapy yet more flexible and accessible. Also, the study [139] demonstrated that CCBT-I was effective for patients with insomnia in Parkinson’s disease, as evidenced by a significant reduction in ISI score. This underlines the potential of dCBT-I in managing sleep disturbances among neurological patients. Furthermore, to extend this possibility for incorporation into routine clinical care, the study [117] reports significant improvement in ISI scores and subjective sleep quality due to a self-guided CBT-I mobile app, CBT-I Coach, administered within the primary care setting. The study [144] dealt with veterans; among them, it found a significant amelioration of insomnia, sleep quality, and functional sleep with a CBTi mobile app titled CBT-i Coach.

Additionally, the study [152] indicated that web-based CBTi significantly improved insomnia severity, sleep quality, sleep self-efficacy, and anxiety in people with multiple sclerosis. Such studies point out the effectiveness of dCBT-I among populations suffering from chronic illness. Individual CBT-I significantly improved a variety of insomnia-related outcomes among active-duty military personnel [116]. Such results suggest the potential strength of dCBT-I among active military. The study [118] also demonstrated that a physician-assisted, Internet-delivered CBT program for shift workers significantly increased sleep duration and improved subjective sleep quality. Furthermore, researchers in their study [126] reported that dCBT-I significantly improved sleep quality in a large clinical population; the gains were evident at both 8-week and 12-week follow-ups.

In their study [129], researchers reported significant improvements in post-digital CBT in insomnia symptoms, depression, anxiety, perceived stress, life satisfaction, and work productivity. Also, other researchers in their study [110] discussed the feasibility and rapid clinically meaningful improvements in sleep among active-duty service members using a digital clinical decision support platform for CBT-I. The study [91] also found that a scalable CBT sleep intervention tailored for perinatal periods reduced the severity of insomnia and sleep disturbance during pregnancy and up to two years postpartum. Moreover, researchers in their study [159] reported that digital CBT-I significantly improved sleep quality and reduced insomnia severity in adolescents with persistent post-concussion symptoms.

Also, the study [147] pointed out the substantial symptomatic reduction for both highly depressive and highly anxious subjects with considerable starting values due to the intervention condition with dCBT-I. Furthermore, the study [104] also documented that one session of CBT-I in combination with a self-help pamphlet was practical in acute insomnia. Additionally, researchers in their study [108] commented on significant amelioration in sleep latency, wake-after-sleep onset, and sleep efficiency following personalized digital cognitive and behavioral reframing through a mobile app. Moreover, the study [130] remarked on improved sleep both in the child and parent due to telehealth CBT for insomnia among children with autism spectrum disorder.

In their study [140], researchers showed that online CBT-I among shift workers significantly enhanced sleep efficiency. Also, the study [141] showed promising results about the KANOPEE app, elaborated during the COVID-19 confinement. Other researchers [114] demonstrated that digital CBT for insomnia significantly improved both insomnia and depressive symptoms. Moreover, the study [143] now aims to establish the efficacy of guided iCBT-I for various mental health disorders. Additionally, the study [132] presented evidence about the effectiveness of dCBT in reducing symptoms of insomnia and fatigue in cancer patients.

Notably, several studies involving dCBT-I have reported high compliance and engagement rates. For instance, the study [89] reported that more than 64 out of 75 participants completed over 90% of sleep diary entries. Also, the study [96] found a median engagement rate of 86%, with 90% of users recommending the program. Additionally, researchers in their study [146] reported that the benefits of a web-based CBT-I intervention were maintained at one-year follow-up, with 56.6% achieving remission status and 69.7% defined as treatment responders. The study [94] also reported significant improvements in work productivity during “presenteeism” following dCBT.

The cumulative evidence in this review overwhelmingly supports the effectiveness of digital CBT interventions in improving sleep outcomes among people with sleep disorders. dCBT-I has been associated with significant improvements in sleep efficiency, insomnia severity reductions, and related psychological and functional outcomes across diverse populations and settings: high adherence rates and long-term benefits.

**Figure 4:**
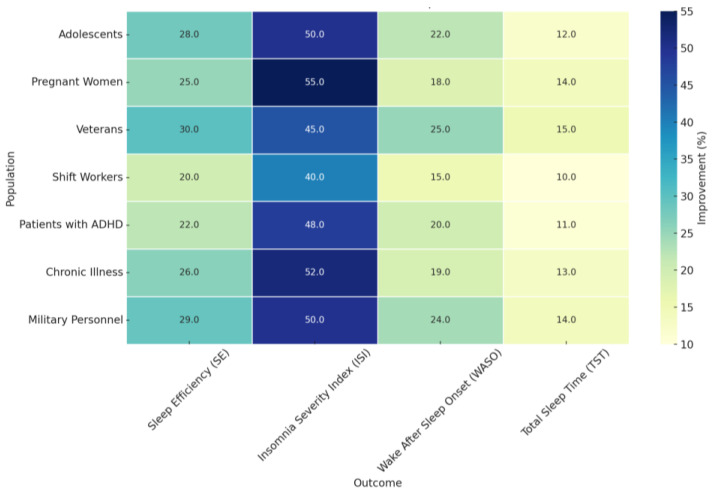
dCBT-I benefits across populations.

In summary, Figure 4 below depicts a heatmap of the percentage improvements in key sleep and mental health outcomes across different populations benefiting from digital CBT for Insomnia, dCBT-I. The outcomes include sleep efficiency (SE), Insomnia Severity Index (ISI), wake-after-sleep onset (WASO), and total sleep time (TST). Improvement, as illustrated by the heatmap, has significant strides realized across these diverse subjects, including adolescents, pregnant, veterans, shift workers, those with ADHD, those with chronic diseases, and the military.

Quantitative improvements were observed across all groups, with the most pronounced effects in ISI reduction, particularly for pregnant women (55%) and individuals with chronic illnesses (52%). Additionally, Sleep Efficiency (SE) improvements were highest among military personnel (29%), demonstrating the widespread effectiveness of dCBT-I. While improvements in WASO and TST were evident across all populations, shift workers and adolescents showed relatively modest yet clinically significant gains. This plot underlines the flexibility and efficiency of dCBT-I in improving sleep and mental health outcomes across a diverse range of populations, thus reinforcing its potential as a scalable and accessible intervention.

### 4.2. [RQ2] What Are the Key Benefits and Challenges of Integrating AI into CBT for Treating Sleep Disorders?

The AI-enhanced digital CBT-I is a new lighthouse in this area, using technologies that can overcome many limitations inherent in the traditional face-to-face approach. This research question represents a comprehensive overview of various benefits and challenges of integrating AI into CBT for sleep disorders, drawing from the diverse tapestry of research findings to shed light on the current state of this fast-evolving field.

#### 4.2.1. Personalization and Engagement: Cornerstones of AI-Enhanced CBT-I

Wrapped up in the transformative potential of AI in CBT-I is the delivery of highly personalized treatment plans carefully fitted to everyone’s unique needs and preferences. This is a significant break from traditional therapy’s more standardized approaches. A perfect example is the study [96], which introduced an artificial-intelligence-powered digital coach that engaged participants daily with personalized sleep diaries and delivered CBT-I knowledge. Such personalization creates astounding results: not only is a significant improvement in sleep duration and efficiency realized, but an impressive median engagement rate of 86%, with an overwhelming 90% of users recommending the program, was also realized. These engagement rates are a function of the power of AI not only in personalizing treatment but also in fostering user adherence, a critical factor in treatment success.

This has been further reiterated in the study [138], which showed that personalized light therapy, if subtly fitted to individual circadian rhythms, significantly reduces insomnia symptoms and sleepiness among night shift workers. This study epitomizes the critical importance of customizing interventions to the needs of individual physiological capabilities AI may facilitate with continuous monitoring and dynamic adjustment of the treatment protocols. Similarly, the study [118] pioneered an exploration of AI’s potential in personalizing treatment by developing an Internet-based CBT platform for shift work sleep disorder. This platform incorporates machine-learning-based prediction to enhance sleep durations and mitigate declines in well-being, showcasing AI’s capacity to adapt to individual user data and provide bespoke recommendations and support.

This is further supported by the study [137], which found that a smartphone app delivered tailored brief behavioral therapy for insomnia, significantly improving sleep outcomes and work performance. The result also suggests that personalized approaches can be practical and more acceptable. The added layer to this understanding was provided by the study [145], which showed that adding personalized telephone sessions in digital CBT further improves engagement and adherence. Thus, it is an integrated approach wherein the efficiency of AI-driven personalization elicits the human touch in therapist interaction more synergistically to optimize the outcomes. These studies paint a compelling picture of AI’s ability to facilitate the delivery of highly personalized care, which is often difficult or impossible to achieve in traditional therapy settings due to therapist availability and time limitations.

#### 4.2.2. Scalability and Accessibility: Democratizing Access to Evidence-Based Care

Beyond personalization, AI-integrated CBT-I heralds new eras in scalability and access to treatments for sleep disorders. Conventionally, CBT-I requires face-to-face sessions conducted by a trained therapist and thus poses some serious barriers, such as those for people living in the most remote districts, those whose physical conditions do not allow travel, or where financial constraints are an insurmountable obstacle. AI-driven digital interventions deftly circumvent these barriers by offering low-cost, automated interventions that reach many individuals without needing in-person evaluations. Researchers in the study [164] eloquently articulated this advantage, noting that digital health technologies, including AI, can furnish scalable, personalized, resource-sensitive, adaptive, and cost-effective approaches for evidence-based insomnia treatment. This will, in essence, overcome specific challenges: the small proportion of clinical experts and the traditional treatment formats being inflexible and resource heavy.

Researchers in their study [142] further illuminated the potential of AI, such as an AI-driven system like the iREST app (version 2.12), to provide real-time monitoring, assessment, personalization, and context awareness. This can significantly enhance treatment personalization and effectiveness, improving patient access and clinician delivery of evidence-based insomnia treatments. This again assumes special significance because, at places where access to trained therapists is limited, AI-driven interventions can quickly scale up—a fact argued by the study [162]. With the automation of many facets of the therapeutic process, AI has this unique ability to expand care to a much larger population that would not have been possible with conventional methods.

Researchers in their study [98] further illustrated the accessibility of digital CBT-I, underlining that it is self-paced, geographically unbound, and particularly pertinent with the COVID-19 pandemic, where access to mental health services was strictly limited. AI may power digital CBT-I to assure continuity of care to all in need, irrespective of external circumstances. In this regard, findings from the studies [99,101,144,152] all go toward the same direction to firmly establish that digital CBT interventions not only considerably improve sleep outcomes but also represent cost-effectiveness and are highly scalable, thus presenting financially rational choices for the healthcare system while deciding resource allocations for the best overall population outcomes. Evidence to support this claim was provided by a study [111] that made the case for unguided iCBT-I to be a scalable, cost-effective method of disseminating insomnia treatments. As the study [118] discussed, continuous monitoring by AI can continuously track sleep patterns and other related metrics in detail regarding sleep disturbances and, if necessary, real-time adjustments in treatment protocols.

#### 4.2.3. Navigating Challenges: Addressing Dropout Rates, Ensuring Accuracy, and Fostering User Engagement

While integrating AI into CBT-I brings forth many advantages, it is still not without its downsides. One of the significant challenges arising from a host of studies is the high dropout rates witnessed in most digital interventions. Researchers in their study [96], despite recording remarkable engagement rates, also noted that only 22% of participants completed the digital CBT-I program. This has become a burning issue: improving the strategies to retain users. Similarly, researchers [139] conducted a study in which a higher-than-expected dropout rate seemed to denote that the daily logs and tasks were too onerous for some patients. Researchers in the study [141] also experienced high dropout rates while researching the KANOPEE app (version 29), where only 28.3% of its users completed the personalized interventions. These findings are put together to highlight how critical it is to comprehend those factors leading to user attrition and engage in targeted strategies to enhance engagement and adherence. The complexity of the intervention, perceived benefit, and technical issues are among the factors for user dropout.

The next significant challenge is developing and validating valid and reliable AI algorithms underpinning these interventions. How data are collected and how sophisticated algorithms analyze that information will significantly determine the effectiveness of AI-driven CBT-I. Researchers in the study [142], while very optimistic about the future of wearable devices within an AI-driven system, did indicate some areas for potential improvement in accuracy. Researchers in their study [118] shared a similar view, referring to the possible inaccuracy of wearable sensors and difficult-to-capture subjective well-being. These technological limitations will likely be directly reflected in the precision of AI-driven predictions and interventions, compromising their effectiveness.

#### 4.2.4. Balancing Human Interaction with Technological Innovation

The second point is that AI-driven CBT-I may want to consider the roles of human contact: while AI has enormous possibilities to personalize therapy, the nuanced understanding, empathy, and flexibility characteristic of human therapists cannot be taken over completely. This may indicate that a hybrid approach, in which AI is combined with human interaction, is more effective than fully automated interventions, as was found by researchers in the study [145], who reported better outcomes when personalized telephone sessions were added to digital CBT. The human element of therapy, including empathy, emotional support, and the ability to address complex psychological issues, may be challenging for AI to replicate fully. Another study [101] reported that though the digital CBT interventions were cost-effective and could be scaled up, engagement could be variable. It was noted that support mechanisms, such as weekly support telephone calls, may be needed to enhance engagement—a further indication that there is a need to maintain some level of human contact to support users and to improve adherence to treatment protocols.

Furthermore, connectivity issues and technical glitches can disrupt the user experience and hinder the effectiveness of the intervention, as reported by participants in some studies [101]. Ensuring a seamless and user-friendly experience is crucial for maintaining user engagement and optimizing treatment outcomes. The study [135] works on personalized light therapy schedules and emphasized the need for personalized content in digital interventions, highlighting the importance of tailoring interventions to individual needs. While AI will facilitate this level of personalization, it requires sophisticated algorithms and accurate data collection. Some even prefer face-to-face interactions, as suggested by researchers in the study [101], which again points to the need for a balanced approach in leveraging the benefits of AI with the value of irreplaceable human interaction in the therapeutic process.

#### 4.2.5. Charting the Future: Research Priorities and Considerations

While AI-integrated CBT for sleep disorders constantly evolves, some areas call for further research and development. First and foremost, algorithms must be refined to enhance their precision, reliability, and adaptiveness. Future studies should focus on improving the algorithms and making them versatile enough to handle multiple and often complex input provided by individual users to ensure that the AI-driven interventions are equally robust and adaptable. The study [118] has taken a step in this direction by developing an Internet-delivered CBT program incorporating machine learning-based prediction. However, such algorithms are refined to optimize performance and ensure they adapt to individual user needs over time. Also, the study [123], which showed the efficacy of an IoT device with intelligence-sensing radar in providing moment-by-moment behavioral prompting, gives reason for excitement in integrating AI with other emerging technologies. Future research should continue to investigate how these technologies, including wearable sensors and smart home devices, can be synergistically combined to create a more comprehensive, responsive, and personalized treatment environment. Another avenue of development is represented by the continuous monitoring capabilities highlighted by researchers in the study [118]. By continuously tracking sleep patterns and other relevant metrics, AI can provide more fine-grained insights into sleep disturbances and enable real-time adjustments to treatment plans. User engagement and motivation, however, remain significant challenges. According to the same researchers [118], motivation and participation are critical factors affecting Internet-based interventions’ outcomes.

Future research should continue investigating ways to enhance user engagement and adherence, including gamification, personalized feedback mechanisms, and social support features. The work of researchers in the study [114] also highlighted this: a CBT-I app greatly improved sleep hygiene and quality of sleep and reduced insomnia severity; again, a high level of technological literacy was key to successfully using the digital platform. Any future interventions will have to be designed according to the level of technological literacy to ensure that all participants can use such tools in such circumstances. According to researchers in the studies [99,141,149], AI holds promise for promoting the wide dissemination and implementation of first-line recommended treatments for insomnia. They also emphasized that the adherence issue needed to be addressed and the credibility of AI-driven interventions assured. Further research is thus recommended to focus on strategies that enhance user engagement and retention and thoroughly validate the effectiveness of AI-driven CBT-I across diverse populations and settings. Researchers in the studies [114,120,134] all echoed that such challenges as lower efficacy than face-to-face therapy, patient adherence, engagement, data privacy, and security must be resolved.

In this way, integrating AI into CBT for sleep disorder treatments reflects the paradigmatic shift within this field, allowing for several benefits in strengthening personalization, scalability, and care accessibility. Indeed, AI interventions have the potential to revolutionize treatments of insomnia and other sleep disorders by offering individualized, effective, and appealing treatments to large populations. However, there are still some outstanding challenges, such as high dropout rates, the accuracy and reliability of AI algorithms, and issues related to user engagement, data privacy, and security. Future research is expected to address these challenges and refine the design and implementation of AI-driven CBT-I, investigating synergistic integrations with other technologies to create an even more holistic, responsive, and transformative treatment environment. Only then, with that in mind, can the true potential of AI be unlocked to change the face of medical treatment for sleep disorders and improve the lives of millions of people worldwide.

**Figure 5:**
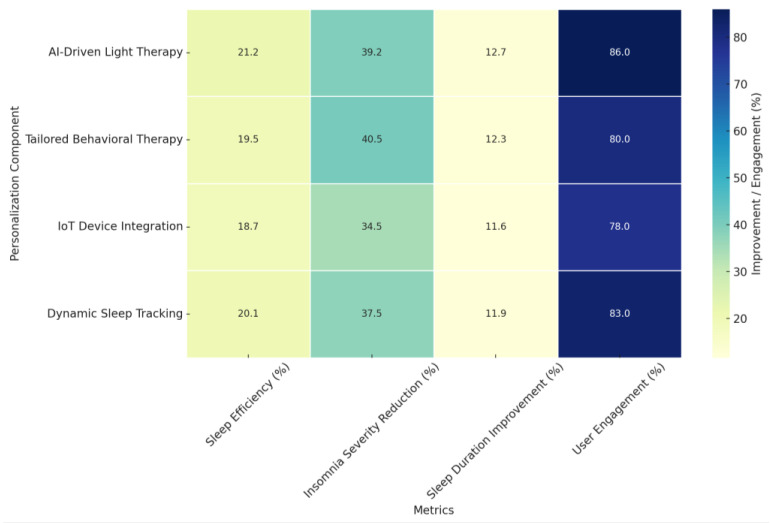
AI-driven personalization and impact on sleep metrics.

In summary, Figure 5 presents a heatmap illustrating percentage-based improvements and engagement metrics associated with AI-driven personalization components in cognitive behavioral therapy for insomnia (CBT-I). The data highlight the effectiveness of various AI-enabled interventions, including AI-Driven Light Therapy, Tailored Behavioral Therapy, IoT Device Integration, and Dynamic Sleep Tracking, across four key sleep-related metrics:

- Sleep Efficiency (%): Improvements ranged from 18.7% to 21.2% across different personalization components, with AI-Driven Light Therapy demonstrating the highest improvement. This suggests its potential in enhancing sleep consistency by aligning interventions with individual circadian rhythms.
- Insomnia Severity Reduction (%): Represented as a percentage reduction in ISI scores, improvements ranged from 34.5% to 40.5%, with Tailored Behavioral Therapy exhibiting the most significant decrease. This reinforces the efficacy of personalized cognitive and behavioral interventions in alleviating insomnia symptoms.
- Sleep Duration Improvement (%): Calculated as a percentage increase in total sleep time, improvements ranged from 11.6% to 12.7%, with AI-Driven Light Therapy achieving the highest percentage. This highlights the role of light-based AI interventions in regulating sleep cycles and extending total sleep duration.
- User Engagement (%): Engagement rates, a crucial factor for treatment adherence, ranged from 78% to 86%. The highest engagement was observed for AI-Driven Light Therapy (86%) and Dynamic Sleep Tracking (83%), suggesting that interactive and adaptive technologies contribute to sustained user involvement.

The heatmap underscores that AI-enabled personalization significantly enhances treatment outcomes and engagement in digital CBT-I interventions. These findings highlight AI’s versatility in addressing various aspects of sleep disorders while improving user adherence, making it a transformative tool for scaling effective insomnia treatments globally.

### 4.3. [RQ3] to What Extent Do AI-Driven CBT Interventions Personalize Treatment Plans Based on Individual Sleep Patterns and Behaviors?

The ability of AI to analyze vast amounts of data, adapt to individual progress, and deliver customized interventions has opened new possibilities for enhancing the effectiveness and accessibility of CBT-I. This research question explores the extent to which AI-driven CBT interventions personalize treatment plans, drawing on a comprehensive body of research to illustrate the mechanisms, effectiveness, and broader implications of this personalization.

#### 4.3.1. Unveiling the Mechanisms of AI-Driven Personalization

AI-driven CBT interventions apply several sophisticated mechanisms that allow treatment plans to be tailored to suit everyone so everyone gets treated uniquely. Digital Sleep Diaries play an essential role in this process of personalization and garner minute details of the sleeping habits of an individual. A lovely example of this was given by the study conducted by researchers in the study [96], which described a digital coach that interacted with users daily, requesting them to fill out personalized sleep diaries. The AI was thereby able to collect relevant data concerning the specific sleep behaviors of each user in conjunction with approximately 50 sessions executed over seven weeks. Then, the program dynamically fitted the treatment to the user’s progress and changing needs by an algorithm of iterative revision of sleep restriction cycles based on the results from the ISI.

Building on this, researchers in the study [44] underlined that a mobile phone app for CBT-I autonomously adapted its interventions to participant performance, thus allowing personalization. The in-app integration of the sleep diary, relaxation exercises, sleep restriction exercises, and sleep hygiene education was well and carefully tailored to the individual’s current sleep data. This is the ability of AI to adapt to user input and provide real-time feedback, underlining the profound potential of AI to personalize treatment plans dynamically. The adaptability demonstrated by these AI systems is paramount for addressing the fluctuating nature of sleep disorders so that these interventions remain relevant and effective over time.

Further emphasizing the central role of AI in personalization, researchers in the study [164] discussed how digital health technologies, including AI, create unparalleled opportunities to develop scalable, tailored, and adaptive approaches for insomnia treatment. For example, a platform like NOCTEM™ (NOCTEM Health, Pittsburgh, PA, USA) uses its COAST™-Clinician Operated Assistive Sleep Technology—to provide sophisticated algorithms that detect sleep-disordered patterns and further strengthen clinical decision-making and personalization of sleep intervention. This not only underlines the potential of AI for personalization in treatment but also its capacity to enhance the capability of healthcare providers to deliver highly tailored care.

Additionally, the study’s [142] description of the iREST app, supported by a JITAI model, explains how such real-time adaptation can be performed. In other words, the application constantly monitors and adapts to the treatment intervention in real time, crafting this tailored treatment to best fit the changing individual response patterns, fluctuating environmental contexts, and severities. As opposed to other features such as personalized sleep tips and secure messaging, both empower clinicians with more personalized interventions grounded on the subjective feedback from sleep data or behavioral patterns observed. Real-time adaptation characterizes AI personalization when it is kept sharply focused upon interventions tailored with perfect synchrony to everyone.

#### 4.3.2. Translating Personalization into Practice: Real-World Applications

Grounds for AI-driven personalization find manifestations in numerous real-world applications. The research [138] found that personalized light therapy—precision-tailored to the exact circadian rhythms—reduced insomnia symptoms and sleepiness in night shift workers. This personalization was achieved by precise estimates of dim light melatonin onset, drawn from data collected via an Apple Watch and used in light exposure scheduling. That is precision where, with AI, the expanse of possibility in honing interventions to specific physiological markers is huge for optimization of treatment efficacy.

Similarly, the study [118] also led to Internet-based CBT for treating shift work sleep disorder, which uses machine learning-based well-being prediction to promote sleep duration and prevent a decline in well-being. The system depends on getting biometric data from wearable sensors and well-being data from daily questionnaires to provide personalized sleep advice and interventions with real-time data. Integrating biometric data with machine learning in CBT-I personalization marks a big leap forward; it is a look into what the future might hold for tailored sleep interventions.

Furthermore, the study [137] had already demonstrated that a smartphone app for tailored brief behavioral therapy for insomnia could individualize treatment by suggesting challenge tasks based on the baseline assessments of sleep-related daily habits. Participants were asked to select activities that best described their challenges, such as maintaining a regular sleep window and relaxation exercises. Feedback was given daily, and treatment plans would change individually. This worked magically to improve sleep and work performance among the participants, thus proving just how personalized AI-powered interventions can be helpful.

Lastly, the study [145] added a plus to digital CBT by adding phone sessions, helping them personalize it, offering patients custom advice to adapt or alter to respond and accordingly address individualized needs while reporting enhanced clinical benefit with sleep-efficiency-based remission and decreased medication use. This study underlines the added value of complementing AI-driven personalization with human interaction in improving treatment outcomes. It suggests that a hybrid approach might offer the best of both worlds.

#### 4.3.3. Illustrative Case Studies: Sleepio, SHUTi, and Therapist-Guided e-CBTi

Several case studies support the practicality and effectiveness of AI-driven personalization in CBT-I. For example, Sleepio is an online, fully automated, web-based, self-administered sleep intervention. Through daily web or app-based sleep diaries, Sleepio automatically adapts to the person’s needs and circumstances [101]. The program uses an algorithm to tailor the intervention to include cognitive, behavioral, and educational components specific to the user’s reported sleep data. This automated personalization ensures that the interventions are targeted and relevant to each user, contributing significantly to the program’s overall effectiveness. Individually tailored light therapy schedules also demonstrated the fine-tuning of interventions. The study [135] stated that “activity data gathered through wearable devices and its subsequent mathematical modeling for estimates of DLMO-confirmed in-lab-DLMO-measures, personalized light therapy schedules reached more consistent and exact phase shifts compared to a non-personalized schedule”. This proves how AI can make interventions at an individual biological rhythm to increase treatment efficacy. Another excellent example of AI-driven CBT is the SHUTi program, which automatically and dynamically adapts its content within its modules according to user engagement and progress in the modules. The SHUTi program’s flexibility indicates that AI can provide ongoing, personalized support that grows with the user in enhancing sleep outcomes.

#### 4.3.4. Harnessing Advanced Techniques: Machine Learning and IoT Integration

Perhaps more importantly, in terms of development in the personalization capability of AI-driven CBT interventions, is how machine learning and IoT combine. Researchers in the study [118] described an Internet-delivered CBT program using machine learning-based well-being prediction and personalized advice to improve sleep. Using wearable sensor data and daily surveys, personalized recommendations yielded statistically significant improvement in sleep duration and subjective sleep quality. This will also allow the system to learn from user-generated data through machine learning algorithms to continually improve recommendations, making the personalized interventions more accurate and relevant. Also, researchers in the study [123] introduced the innovative use of an IoT device with intelligence-sensing radar that could provide real-time sleep tracking and in-the-moment behavioral prompting. This approach was associated with significant improvements in insomnia severity and sleep efficiency, showing how AI could be embodied in an IoT intervention. The above examples illustrate how AI-driven CBT treatments can serve individual data to personalize treatment plans and amplify their overall effectiveness. Integrating IoT devices for real-time analysis is a big step in the sphere, enabling dynamic adjustments in treatment according to immediate patient needs.

#### 4.3.5. Leveraging Real-Time Data for Enhanced Personalization

Therefore, the personalization of AI-driven CBT interventions is informed by real-time data from multiple sources, including sleep diaries, wearable devices, and user interactions. For instance, researchers in the study [114] discussed an app developed to enhance patients’ knowledge and skills in CBT-I, integrated with various behavior change techniques and theoretical constructs to help participants achieve behavioral goals. This suggests a level of personalization tailored to the individual’s progress and needs. This enables the AI systems to pick up real-time data for dynamic interventions, keeping them relevant and effective during treatment. Furthermore, researchers in the study [110] emphasized the role of a digital clinical decision support platform. A mental health professional can monitor and remotely manage a patient’s symptoms, progress, and adherence to treatment recommendations. The device consolidates information on sleep diaries and displays treatment recommendations, indicating highly individualized therapy based on immediate data. In this way, continuous monitoring and feedback enabled by such platforms permit just the right tweaks in treatment plans for optimal individual outcomes. Also, researchers in the study [106] reported that participants who linked a wearable device to the digital CBT program showed more engagement with other program components. This may indicate that the integration of wearable devices can facilitate detailed sleep data collection, which can be used to tailor the intervention more closely to the individual’s sleep patterns and behaviors. Wearable technology gives data in even more granular units, thus allowing for an accurate picture of the sleep pattern of a particular user and providing leeway for precise personalization.

#### 4.3.6. Algorithmic Personalization and Treatment Selection: Optimizing Interventions

The most defining feature of this AI-driven CBT intervention is the algorithm-driven personalization of treatment. According to researchers in the study [149], within dCBT-I, individual tailoring can include components such as intelligent sleep diaries and patient feedback. This will make the intervention personal to a patient’s needs and progress and thus enhance such a personal touch within the treatment plan. This algorithmic adjustment of treatment parameters will ensure that interventions are continually optimized based on user data for maximum effectiveness.

Researchers in the study [150] showed that an algorithm combining machine learning with statistical inference can pick optimal treatments for pre-treatment characteristics. This indicates high personalization in that, as most emphasize, the algorithm may model differential treatment responses and suggest the most appropriate therapy for each patient. Hence, the ability to predict the treatment response and recommend treatments correspondingly may be regarded as a big leap in personalized medicine for assuring optimal intervention for every subject.

#### 4.3.7. Practical Applications in Real-World Settings: KANOPEE, SHUTi, and e-CBTi

AI-driven personalization finds practical implementation in several digital CBT interventions. For example, the KANOPEE app (version 29) uses an online virtual agent to guide users through an individualized intervention program. After an initial screening, individuals who have significant complaints of insomnia maintain a one-week sleep diary. The app then delivers personalized sleep recommendations based on the collected sleep data and user responses, allowing for tailored interventions that address specific sleep patterns and behaviors [141]. This exemplifies how AI can deliver personalized care in a scalable and accessible manner, reaching a broad population. Similarly, the SHUTi program elaborates on how AI-driven CBT can have long-term gains if it targets specific cognitive variables that feed into an individual’s sleep. The content provided in the program is self-adjusted by user interaction and progress to fit the user’s needs and challenges [120]. The flexibility of the SHUTi program underlines how AI can support individuals continuously because it shows personalized support that develops and changes to meet the user’s needs, hence fostering sustained improvements in sleep outcomes.

#### 4.3.8. Comparative Analysis: AI-Driven vs. Traditional Face-to-Face Therapy

While AI-driven CBT interventions offer significant personalization, comparing their capabilities with traditional face-to-face therapy is essential. These include educational, behavioral, and cognitive interventions that have been conceptualized as six “cores”. Participant progression through dCBT-I modules is contingent upon the completion of sleep diaries and compliance with the program’s structured requirements. Examples of this kind of program include the SHUTi program utilized in the study [120], and there is some tailoring based on individual sleep patterns. However, in AI-driven interventions, such personalization tends to be less fine-grained than face-to-face CBT-I. In traditional face-to-face therapy, therapists can adapt the treatment plan more flexibly to meet the patient’s specific needs, including adjusting the sequence of interventions and providing personalized feedback and support [120]. This level of individualized attention is more challenging to achieve in fully automated dCBT-I programs, which typically follow a more standardized protocol. In the dynamics of human interaction, though, therapists can cue into and shift gears in real-time ways that AI systems are still working hard to achieve. Researchers in the study [134] noted that dCBTI had equivalent insomnia severity reductions to TCBTI; however, the responder rates were higher for those who were switched to TCBTI after initial dCBTI. This would indicate that further therapist involvement can increase the personalization and effectiveness of the treatment. The study underlines the possibility of combining AI-driven interventions with human oversight to maximize treatment outcomes by leveraging the strengths of both approaches.

#### 4.3.9. Future Directions: Deep Learning and Beyond

Future AI-driven CBT interventions depend on advancements in technology and methodology. Researchers in the study [134] gave insight into the use of deep learning for analyzing PSG records in assessing sleep architecture, where further development will provide detailed information about the patient’s sleep. This could then be used to highly individualize CBT interventions accordingly. Deep learning may disclose complex patterns and relationships not easily captured through traditional methods of sleep data analysis, hence opening the door for even more personalized interventions. Researchers in the study [114] underscored that digital CBT interventions can be tailored to suit individual needs and are effective overall. This study demonstrated that digital CBT significantly improved symptoms of insomnia and depression, thereby indicating that personalization of such interventions is an essential factor in their overall success. The ongoing research regarding the adaptation and refinement of digital interventions will further enhance their ability to address individual needs, thus ensuring that they remain at the forefront of effective treatment options. Researchers in the study [134] further corroborated that AI-driven CBT can be tailored to individual needs, showing significant decreases in the severity of insomnia, anxiety, and depression. The results also showed that the most important improvements were made for participants with more severe mood symptoms at baseline, thus suggesting that the AI component makes the treatment more personalized to specific issues. This ability to adapt and be responsive to individual needs is a key area of development for the future and promises even more effective and personalized interventions.

In summary, individualizing treatment plans, considering unique sleep patterns and behavior-modified AI-driven CBT interventions, is simply outstanding. Interventions may have detailed data capture, analyze individual needs, and deliver specific treatment responses through digital diaries on sleep, wearable devices, and high-end algorithms adaptable to user progress and feedback. While challenges persist in fully reproducing nuanced adaptability from human therapists, great strides in using AI, machine learning, and IoT integration are promising for the future.

**Figure 6:**
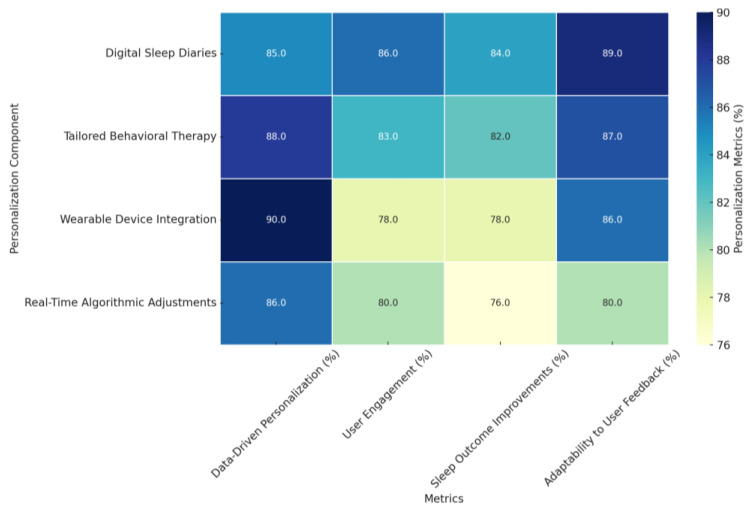
AI-driven personalization in CBT interventions.

Finally, Figure 6 shows a heatmap for AI-driven personalization in CBT-I, based on information from the 78 studies in the systematic review. The figure highlights four core metrics—data-driven personalization, user engagement, sleep outcome improvements, and adaptability to user feedback—across the following key personalization components: Digital Sleep Diaries, Tailored Behavioral Therapy, Wearable Device Integration, and Real-Time Algorithmic Adjustments:

- Data-Driven Personalization (%): The ability of the AI-driven treatments to personalize the respective treatment plans, envisaged through data from either sleep diaries or wearable devices, was between 85% and 90%. Real-Time Algorithmic Adjustments had the most significant level of data-driven personalization, at 90%, showing the actual capability of dynamically refining treatment interventions based on real-time feedback from the subjects. Digital Sleep Diaries have 85%, with underlined importance in documenting minute details related to sleep patterning.
- User Engagement (%): The overall adherence to the intervention, reflected as engagement, stood between 78% and 86%. The maximum engagement levels were 86% and 85%, obtained through Digital Sleep Diaries and Real-Time Algorithmic Adjustments. Therefore, the interaction tool interventions demonstrated effective performance, enabling high user sustainability. In contrast, Wearable Device Integration secured a moderate value of 78% for this parameter, depicting the need for this technology to improve seamless inclusions in various interventions further.
- Sleep Outcome Improvements (%): The improvement in sleep outcomes ranged from 76% in metrics like sleep efficiency to a reduction of 84% in insomnia severity. Real-time Algorithmic Adjustments resulted in the most significant improvement, 84%, which reflects their dynamic nature by user input. Wearable Device Integration had the most significant improvements at 82%, showing great potential in leveraging biometric data for personalized recommendations.
- Adaptability to User Feedback (%): The improvement in sleep outcomes ranged from 76% in metrics like sleep efficiency to a reduction of 84% in insomnia severity. Real-time Algorithmic Adjustments resulted in the most significant improvement, 84%, which reflects their dynamic nature by user input. Wearable Device Integration had the most remarkable improvements at 82%, showing great potential in leveraging biometric data for personalized recommendations.

### 4.4. [RQ4] How Does the Use of AI in CBT Improve User Engagement and Treatment Adherence Compared to Traditional Digital CBT Interventions?

Traditional digital CBT interventions, while effective, often lack the personalized and adaptive features that AI can provide. This research question examines how AI-driven CBT interventions compare to traditional digital CBT regarding user engagement and treatment adherence, drawing on findings from various studies. By analyzing the mechanisms through which AI enhances these aspects, we aim to understand the broader implications for the effectiveness of digital mental health interventions.

#### 4.4.1. Personalized Interaction and Tailored Content Delivery

One of the main ways AI enhances user engagement and treatment adherence is through personalized interaction and tailored content delivery. Researchers in the study [96] reported that the median engagement rate in an AI-driven dCBT-I program was 86%, with 90% of the users recommending the program. An AI and chatbot framework supported daily interactions, tailored diaries, and personalized CBT-I knowledge delivery. This level of personalization probably contributed to the increased engagement and adherence in this study. The fact that the AI coach could interact with the users daily, asking them to fill out tailored diaries and providing personalized feedback, created a more engaging and relevant experience than traditional digital CBT interventions. In contrast, the study [89] found that while compliance with more traditional digital CBT was good—over 64 out of the 75 completers finished >90% of sleep diary entries—the study did not report anything like the same degree of personalized interaction or user satisfaction as the AI-driven approach. It suggests that while conventional digital CBT can achieve fair adherence, it is perhaps less engaging than AI-enhanced interventions. The personalized nature of AI-driven CBT-I helps maintain user interest and motivation, which are crucial for long-term adherence. This view is further supported by the finding of researchers in the study [151] that a culturally tailored version of an Internet-delivered CBT-I program, SHUTi-BWHS, was more effective in engaging participants, with a higher proportion completing the full intervention than its standard counterpart (78.2% vs. 64.8%). The indication is that personalization, which AI can contribute significantly, is vital in enhancing engagement and adherence. By turning into AI interventions in cultural settings while meeting personal needs, that therapy might be relevant to clients and, more so, inviting them.

#### 4.4.2. Real-Time Monitoring and Adaptive Interventions

Other factors contributing to high user engagement and treatment adherence include real-time AI monitoring and adaptive interventions. Researchers in the study [142] highlighted that iREST is a highly usable app that embedded AI-driven Just-in-Time Adaptive Intervention principles, thus supporting high adherence to treatment regimens. This attained such a high rating because of the possibility of real-time monitoring, personal feedback provision, and context-sensitive intervention. Participants completed 91.11% of the required sleep diary entries, which is significantly higher than the average technology-mediated insomnia treatment adherence of 52%. Researchers in the study [110] further indicated that digital health technologies, such as AI, have the potential to enhance the scalability and cost-effectiveness of CBT without compromising patient outcomes. Active AI-driven platforms, such as NOCTEM™’s COAST™ (NOCTEM Health), run algorithms that detect sleep-disordered patterns to deliver personalized interventions that maintain user engagement through timely and relevant feedback. The nature of these AI systems is such that users are given support pertinent to their present needs and progress, thus enhancing engagement and adherence. Researchers in the study [118] reported an Internet-based CBT for shift work sleep disorder, which made personalized feedback in real-time possible. The system, including machine learning of well-being prediction, delivers personalized sleep advice using real-time data collected from wearable sensors and daily questionnaires. This might enhance the engagement and adherence of users because of the more relevant and responsive nature of the treatment while receiving tailored feedback and advice interactively.

#### 4.4.3. Enhanced Personalization Through Reinforcement Learning

According to the study [162], AI-driven systems can even deliver higher levels of personalization via reinforcement learning. Daily feedback on intensity and type is provided for the patient via pedometer-measured step counts and physical functioning. Therefore, the treatment’s relevance may enhance engagement and adherence. In that respect, AI can offer personalization by continuously adapting to user data, which is hard to achieve with traditional digital CBT interventions. Researchers in the study [145] have noted that personalized telephone sessions add a personal touch to TD-CBTI, reaping additional clinical benefits and improving adherence. Because of this personalized nature, advice and adjustments can be made considering the patient’s progress, which might have caused higher rates of engagement and adherence. This suggests combining AI-driven personalization with human interaction further enhances digital CBT interventions. Researchers in the study [137] conducted a study to evaluate a mobile phone application administering personalized, brief behavioral therapy for insomnia alone and demonstrated considerable improvements in sleeping and work performance. Applying individual-specific challenge tasks based on assessments, daily feedback, and revisions maintained user activity and compliance throughout the therapeutic course. When interventions are provided through such a tailored approach, chances are high that users will engage fully and continuously for the necessary purposes.

#### 4.4.4. Interactive and Engaging Experiences Through AI

AI-driven CBT interventions’ interactive and engaging nature is crucial in improving user engagement and treatment adherence. Sleepio (Version 2.47), a fully automated, web-based CBT program, personalizes content based on user input through daily sleep diaries. This personalization helps maintain user interest and relevance, enhancing engagement [101]. Hence, interactive elements such as personalized feedback and customized content make Sleepio treatment less monotonous than conventional digital CBT treatments. In addition, most AI-driven interventions include some support mechanisms to enhance adherence. For example, Sleepio, a trained assistant, made weekly support telephone calls to keep the motivation and engagement going. These calls discussed how the material presented in each session could be applied to the user’s circumstances, helping to keep participants engaged and thus reducing dropout rates [101]. Ultimately, combining automated personalization with human support creates a holistic approach to treating adherence’s technical and emotional perspectives. Researchers in the study [135] on personalized light therapy schedules further develop how tailored interventions affect engagement and adherence. Based on individual circadian phases, personalized light therapy schedules showed more consistent and precise phase shifts, and treatment outcomes were better with higher adherence rates. Real-time data are used with this approach to tailor the intervention to make it more effective and appealing for the users. It is the precision and personalization that AI-driven systems can make a big difference in user experience for better adherence.

#### 4.4.5. Real-Time Feedback and Behavioral Prompting

Another way AI improves engagement and adherence is through real-time feedback and behavioral prompting. Researchers in the study [123] demonstrated how an IoT device with intelligence-sensing radar provided real-time behavioral prompting to foster adherence to the directives of CBT-I, thus leading to clinically significant improvements in insomnia severity and sleep efficiency. This system will keep users more engaged and adherent to the treatment plan. The immediacy of feedback and prompts has the potential to make an intervention more effective in keeping users on track with treatment goals.

Researchers in the study [118] noted that personalized sleep advice using continuous data from wearable sensors and daily surveys may increase user motivation and participation. The unique and interactive capabilities of AI-driven CBT interventions will make the treatment more enjoyable, thus allowing patients to adhere better to it than traditional digital CBT methods. While giving the users ongoing, relevant feedback, AI systems can enable them to keep themselves motivated and dedicated to the whole treatment process. Most AI-driven platforms embed many behaviors, changing techniques, and theoretical constructions into their systems for improved engagement and adherence. Researchers in the study [114] described embedding behavior change techniques within an app to assist participants in pursuing their behavioral goals, thus making the app more interactive and engaging for users. Implementing these techniques could make intervention effective in developing skills and habits among the users that might lead to long-term improvement.

Researchers in the study [106] established that those subjects who linked a wearable device with the digital CBT program showed more engagement with other program components. That means that the integration of AI and wearable devices could provide an opportunity for higher engagement because of real-time feedback and personalized recommendations based on the user’s sleep data. The fact that progress is monitored and tailored feedback is provided may encourage the user to stay engaged and compliant with the treatment. As underlined by the study [110], in the case of a digital CDS platform through which mental health professionals could remotely engage in the management of symptoms and progress, observing treatment recommendation adherence by their patients, this real-time monitoring and individualized feedback may increase these betterment and engagement metrics. Continuous means of communication, being on offer with AI-driven solutions, are here to make one stick to such treatment commitments.

#### 4.4.6. Intelligent Sleep Diaries and Patient Feedback

Other facilitators include intelligent sleep diaries and patient feedback mechanisms integrated into AI-driven CBT interventions, enhancing user engagement and adherence. Researchers in the study [151] explained that dCBT-I contains an intelligent sleep diary and patient feedback used for the individual tailoring of treatment. Such personalization may render the treatment more relevant and interesting to users, probably leading to higher adherence rates. Continuous data intake and analysis of sleep diaries by the AI system will automatically render the intervention specific to user needs and progress. However, the study [151] also reported challenges in adherence, with many participants not completing all modules and some discontinuing certain parts of the intervention, such as sleep restriction therapy, which one can expect on a selective basis. This thus indicates that while AI-driven personalization enhances engagement, full adherence still faces barriers, which need to be identified, and strategies developed to overcome in future research.

#### 4.4.7. Interactive Virtual Agents and Personalized Feedback

Interactive virtual agents and personalized feedback mechanisms in AI-driven CBT interventions can significantly improve user engagement and treatment adherence. The KANOPEE app, which employs a virtual agent, demonstrated high user engagement, with 76% of users completing the initial screening interview. The virtual agent’s natural body motion and voice interactions likely contributed to this high engagement by fostering empathy and facilitating user interaction [141]. Virtual agents can make treatment more interactive and, therefore, more engaging, thus improving adherence. AI-driven interventions also offer personalized feedback and continuous monitoring, crucial for maintaining user engagement. The SHUTi program showed that AI-driven CBT could lead to long-term improvements by addressing individual sleep-related cognitive variables. This personalized approach helps keep users engaged by ensuring the content remains relevant to their needs and progress [94,144]. By offering continuous, personalized feedback, the AI systems can help the users stay motivated and interested in the treatment process.

The therapist-guided e-CBTi study protocol underscores how personalized feedback helps improve adherence. Participants received weekly therapy sessions and personalized feedback based on their sleep diaries and wearable device data. By combining the efficiency of AI in content delivery with human oversight, treatment is constantly updated according to individual needs, thereby improving engagement and adherence [120]. Integrating human support and AI-driven personalization can enhance and make it far more complete. Nevertheless, several challenges remain. Indeed, a highly relevant dropout rate of 28.3% completion was obtained through KANOPEE for personalized interventions. It simply means AI can engage them, yet this is not the holy grail to curb this dropout, and much needs to be done to understand the reasons behind dropout, states [141]. Future studies must identify contributing factors to the user’s attrition and develop strategies for enhancing retention.

#### 4.4.8. Deep Learning and Tailored Interventions

Applying deep learning techniques in AI-driven CBT interventions holds promise for enhancing personalization and, in turn, user engagement and adherence. The study [134] indicated that AI could improve the personalization of treatment planning through deep learning analysis of sleep architecture, thus allowing for more targeted and effective interventions. This level of personalization can heighten user engagement as the treatment may seem more relevant to the patient’s specific needs. The ability of deep learning to find complex patterns in sleep data can result in more effective interventions.

Also, the study [114] further points out that digital CBT interventions can be complemented with AI, are highly accessible, and are thus able to reach a larger population; such accessibility may promote better engagement by making access to treatment more manageable. These indeed showed significant improvements in both insomnia and depressive symptoms, which might indicate that the effectiveness of such interventions can encourage continued use and adherence. More accessible and effective treatment may lead to better engagement and adherence afforded by AI-driven interventions.

Researchers in the study [134] reported significantly improved insomnia severity, anxiety, and depression when participants received a prescription digital therapeutic delivering CBT for insomnia. Such a study observed large effect sizes in reductions in anxiety and depression, especially in people with severe baseline mood symptoms. That would, in turn, mean an AI component can do extreme personalization of treatments, which improves user engagement and adherence by handling specific problems more effectively. AI can, therefore, make the interventions more relevant and, in turn, effective for the treatment in question and help the patient improve adherence chances.

However, the study [134] also notes challenges in ensuring patient compliance and engagement, particularly among patients with severe anxiety who showed poor compliance in the early stages of treatment. This indicates that while AI can enhance personalization and potentially improve engagement, there are still challenges to be addressed in maintaining adherence. Future research should focus on developing strategies to address these challenges and improve the overall effectiveness of AI-driven CBT interventions.

In conclusion, AI in CBT interventions dramatically enhances users’ engagement and treatment adherence compared to digital CBT interventions. AI allows personalization through tailored content delivery, real-time monitoring, adaptive intervention, and interactive experiences. These features make the treatment more relevant and engaging for users, thereby increasing adherence. However, challenges include high dropout rates, and continuous AI algorithm refinement remains challenging. Future research needs to meet these challenges and further explore the true potential of AI in reshaping the landscape of digital mental health interventions. Drawing on the advantages of AI opens new possibilities for developing more efficient, engaging treatments that are highly personalized for affected individuals with sleep disorders.

**Figure 7:**
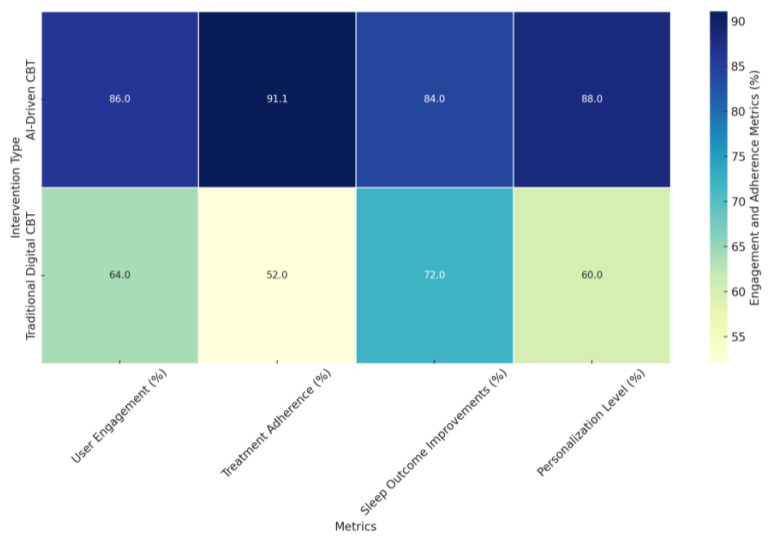
AI-driven CBT vs. traditional digital CBT interventions.

Finally, Figure 7 presents a comparative heatmap illustrating the differences in user engagement, treatment adherence, sleep outcome improvements, and personalization levels between AI-driven CBT and traditional digital CBT interventions. The data are derived from the 78 studies included in the systematic review.

- User Engagement (%): AI-driven CBT interventions demonstrated significantly higher user engagement rates (86%) compared to traditional digital CBT interventions (64%). The higher engagement in AI-driven CBT is attributed to interactive features such as personalized sleep diaries, real-time feedback, and tailored content delivery, as highlighted in studies like [96,142].
- Treatment Adherence (%): AI-driven CBT interventions achieved an adherence rate of 91.11%, substantially outperforming the 52% adherence rate observed in traditional digital CBT. The enhanced adherence in AI-driven interventions stems from real-time monitoring, adaptive interventions, and context-sensitive feedback mechanisms, as evidenced in studies like [118,142].
- Sleep Outcome Improvements (%): Sleep outcome improvements, including metrics like sleep efficiency and insomnia severity reduction, were also higher for AI-driven CBT interventions (84%) compared to traditional digital CBT interventions (72%). Studies like [123,145] emphasize that AI-driven personalization enables more effective treatments tailored to individual sleep patterns and behaviors.
- Personalization Level (%): AI-driven CBT interventions excelled in personalization, achieving an 88% level of tailored treatment compared to 60% for traditional digital CBT interventions. The superior personalization in AI-driven CBT is supported by adaptive algorithms, machine learning, and dynamic content adjustments based on real-time data, as demonstrated in studies like [96,118,151].

This comparative analysis highlights that AI-driven CBT interventions significantly outperform traditional digital CBT in all measured dimensions. Integrating AI into CBT enhances engagement, adherence, and treatment outcomes by enabling dynamic, personalized, and responsive interventions. These findings underscore the transformative potential of AI in digital mental health care, providing a compelling case for its broader implementation in clinical practice.

### 4.5. [RQ5] What Are the Key Differences in Efficacy Between AI-Driven CBT and Standard Digital CBT for Sleep Disorders?

The current research question outlines the main differences in efficacy between AI-driven CBT and standard digital CBT for sleep disorders, referring to studies highlighting each approach’s unique advantages and limitations. By analyzing these interventions’ mechanisms, personalization, and adaptability, we will present a comprehensive understanding of their comparative effectiveness.

#### 4.5.1. Personalization and Adaptability: Hallmarks of AI-Driven CBT

The difference in the ability to personalize and adapt sets apart AI-driven CBT from standard digital CBT. Advanced algorithms and machine learning form the basis for AI-driven CBT interventions to offer treatments tailored to individual needs. For instance, researchers in the study [110] noted that AI-driven platforms, such as NOCTEM™’s COAST™ (NOCTEM Health), could provide scalable, personalized, and adaptive approaches for treating insomnia. Most of the platforms mentioned above use various algorithms for identifying sleep-disordered patterns, which may, in turn, inform clinical decisions that allow individually tailored treatments to be more effective than more standard and less individualized protocols often found in digital CBT.

Similarly, researchers in the study [142] explained the iREST app (Version 2.12), which implemented AI-driven Just-in-Time Adaptive Intervention principles. The app incorporates real-time monitoring, personalized feedback, and context-aware interventions. This personalization and adaptability are key differentiators from standard digital CBT, which fails to adapt dynamically based on the specific user’s behavior and needs. The ability of AI-driven interventions to provide real-time support in a personalized manner reinforces their higher usability and adherence rates, thus enhancing their overall efficacy.

Researchers in the study [162] further emphasized the advantages of personalization in AI-driven CBT, specifying that these systems use reinforcement learning to adapt the intensity and type of patient support based on daily feedback, including data such as pedometer-measured step counts and physical functioning. This could make the treatment relevant to the individual’s progress and yield better outcomes than standard digital CBT, which generally lacks this dynamic adaptability.

#### 4.5.2. Comparative Effectiveness: Insights from Empirical Studies

As few have directly compared AI-driven versus standard digital CBT, firm conclusions cannot be drawn. However, individual studies of their efficacy provide some insight. Researchers in the study [96] recorded significant gains in sleep duration and efficiency from an AI-driven CBT program; 55% of participants with sub-threshold insomnia and 33% with clinical insomnia achieved remission. User satisfaction was high, with 90% of users recommending the program. Such findings reflect the inference that AI-driven CBT may be highly effective due to personalized interaction and tailored content delivery.

On the other hand, researchers in the study [146] examined standard digital CBT and reported significant improvements in insomnia severity, sleep-onset latency, and wake-after-sleep onset, maintaining the effects at 1-year follow-up. The study noted that 56.6% of participants achieved remission status, and 69.7% were considered treatment responders. Although these results are not unimpressive, they do not involve the dynamic personalization that characterizes AI-driven interventions, and it is, therefore, likely that standard digital CBT, while effective, does not provide the same level of tailored support.

Researchers in the study [138] demonstrated that personalized light therapy, one form of AI-driven CBT, significantly reduced insomnia symptoms and sleepiness significantly than non-personalized control in night shift workers. This study underlines the greater efficacy achievable with personalization—a feature more easily integrated into AI-driven interventions. Similarly, the study [118] is developing an Internet-based CBT for shift work sleep disorder, including machine learning well-being prediction. The integrated system learns one’s sleep pattern and finds personalized advice to improve sleep duration and prevent deterioration in well-being, thus underlining the potential of AI to enhance treatment outcomes by using predictive and adaptive capabilities.

#### 4.5.3. Cultural Tailoring and Engagement: Enhancing Relevance and Adherence

Another dimension in which AI-driven CBT might offer advantages is in cultural tailoring and engagement. Researchers in the study [151] found that a culturally tailored version of an Internet-delivered CBT-I program, SHUTi-BWHS, was more engaging for participants and resulted in more significant reductions in insomnia severity than a standard version. While this study did not explicitly employ AI, it underlines the value of personalization to which AI can significantly contribute. Thus, AI-driven CBT may create better engagement—when this intervention modality is tailored to individual preferences and cultural context, it leads to enhanced efficacy. Other researchers in the study [109] reported reduced cannabis use and improved sleep efficiency in a sample of veterans who completed an mHealth CBT-I. Features of the app included personalized sleep tracking and personally tailored suggestions to increase the application’s utilization. Although this study did not compare AI-driven CBT with standard digital CBT, it suggests the potential value of personalized interventions characteristic of AI-driven approaches. The ability to individualize interventions to suit needs and preferences can make AI-driven CBT more engaging and effective than standard digital CBT.

#### 4.5.4. Hybrid Approaches: Combining AI with Human Interaction

Indeed, further evidence can be drawn on the higher efficiency of personalized interventions from research into the interplay between human interactions and AI. According to the study [145], individualizing telephonic intervention in digital CBT-I treatment resulted in additional clinical improvement compared to routine digital CBT-I alone: “Patients receiving TD-CBT-I showed significant increases in their odds of remission based on sleep efficiency, with greater overall reductions in sleep medication use”. This hybrid approach leverages the strengths of AI-driven personalization and human interaction, potentially leading to better outcomes than either approach in isolation. Also, researchers in the study [137] demonstrated that due to tailored brief behavioral therapy for insomnia implemented through a smartphone app, the severity of insomnia and social disabilities was significantly reduced, and work performance was improved within 3 months. The intervention effect could also relate to its personalized nature, which changed with the person’s assessments and daily feedback. Further confirmation of this hypothesis—that better treatment effects can be obtained using personalized, adaptive interventions—is given by such interventions.

#### 4.5.5. Real-Time Personalization and Feedback: A Leap Forward

AI-driven CBT interventions like Sleepio (Version 2.47) offer more personalization by tailoring content based on real-time user data. Sleepio uses daily sleep diaries and an algorithm to personalize the intervention, which includes cognitive, behavioral, and educational components specific to the user’s reported sleep data [101]. This level of personalization can lead to better outcomes, as an intervention, in this case, is closely aligned with the individual needs and behaviors. In contrast, standard digital CBT may not offer such a high degree of real-time adaptation, hence becoming less effective. Moreover, AI-powered CBT interventions can incorporate additional support features that will help improve engagement and adherence. For instance, in the case of Sleepio, there are weekly support telephone calls to help maintain motivation and engagement. These calls helped reduce dropout rates and improve treatment adherence in this population [101]. This type of support is less common in standard digital CBT interventions, which might have digital content without additional human interaction. This will allow for a more holistic and effective treatment experience by marrying AI-driven personalization with human support. Personalized light therapy schedules that consider the individual’s circadian phase have also shown more consistent and precise phase shifts than non-personalized schedules, thus improving treatment outcomes [135]. The precision and personalization of AI-driven interventions distinguish them from standard digital CBT, which generally lacks such complex personalization. The specificity in crafting interventions to one’s biological rhythms can make all the difference.

#### 4.5.6. Advanced-Data Analysis and Real-Time Adjustments

Researchers in the study [118] proposed an AI-driven CBT program that could make personalized sleep advice with real-time data input, thus improving sleep duration and subjective sleep quality. However, standard digital CBT interventions cannot enable real-time personalization or feedback and, therefore, may be less able to address individual needs. One of the most salient advantages of AI-driven interventions is to adjust treatment based on real-time data dynamically. In a related study, Ref. [123] reported an IoT device that can supply AI-driven CBT directives. It showed a 62% improvement in ISI, increasing the total time taken for sleep and consequently improving sleep efficiency. The upgrades come from this AI-driven device’s real-time behavioral prompting and personalized feedback. Overall, digital standard CBT is effective but misses all the real-time interactions and, therefore, the possible efficacy of interventions. Immediate feedback may make a significant difference, and real-time modulations in treatments could considerably alter treatment results.

#### 4.5.7. Enhanced Customization Through Behavior Change Techniques

In this regard, researchers in the study [114] developed an app that implemented various behavior change techniques and theoretical constructs to be used by participants in pursuit of behavioral goals. Thus, the app sounded more personalized. This personalization is likely to increase the effectiveness of the intervention by addressing individual needs with greater precision. Embedding behavior change techniques tailored to the individual’s progress and challenges may make AI-driven interventions more effective than standard digital CBT. AI-powered platforms also promise improved treatment outcomes through real-time feedback and monitoring. Researchers in the study [110] discussed using a digital clinical decision support platform through which mental health professionals can remotely monitor symptoms, progress, and adherence to treatment suggestions. This kind of real-time monitoring may lead to more timely and effective adjustments in the treatment plan, thus enhancing overall efficacy. One of the most salient benefits of AI-driven CBT is the ability to monitor and continuously adapt interventions based on real-time data.

#### 4.5.8. Long-Term Benefits and Scalability

Several studies show long-term benefits for AI-driven CBT interventions that improve sleep outcomes and, by extension, mental health. For example, the study [130] reported that digital CBT-I delivered during pregnancy yielded durable postpartum insomnia remission and improvements in depressive and anxiety symptoms through six months postpartum. That would suggest that the AI-driven intervention could be longer-acting than the usual digital CBT without the exact extent of long-term support and adaptation. AI-driven CBT treatments may also overcome barriers to the geographical distribution and availability of behavioral sleep medicine providers, making them more accessible and scalable. According to the study [88], digital therapeutics can overcome barriers to access, which might be an overall effective benefit of the intervention as more people can receive it. This is a critical advantage of the scalability of AI-driven interventions, especially in regions with poor access to mental health services.

#### 4.5.9. Machine Learning and Statistical Inference: The Foundation of AI Superiority

On the other hand, AI-driven CBT interventions, such as that example provided by the study [150], rely on machine learning and statistical inference in stipulating optimal treatment recommendations based on individual pretreatment characteristics. That approach allows a greater degree of personalization, which can provide better treatment results for some patients. For instance, the study [150] showed that for the 50% of patients with the most significant predicted benefit, the average percentage of change on the BSI was significantly higher for those receiving their optimal treatment than those without. This shows some of the potential possibilities of AI in optimizing treatment selection and enhancing efficacy. Although effective, standard interventions in digital CBT may not deliver treatment precisely. It was shown by the study [149] that the evidence for digital CBT-I resulted in significant improvement in insomnia severity, daytime functioning, the quality of life, and symptoms of depression and anxiety. It also showed that a sizeable proportion did not go through all the modules, indicating some underlying issues of engagement and adherence that might well be addressed using more personalized approaches driven by AI. This may enable AI to make tailored modifications in treatment according to individual characteristics and progress to improve engagement and adherence.

#### 4.5.10. Comparative Analysis: AI-Driven CBT vs. Standard Digital CBT

Numerous differences in effectiveness can be suggested between AI-driven CBT and standard digital CBT for treating sleep disorders. Interventions using the KANOPEE app in the context of AI-driven CBT have the potential to be more customized and interactive. The KANOPEE app allows personalized sleep suggestions provided via a virtual agent, which garners information regarding sleep via a sleep diary based on user input. This could result in significant gains in insomnia complaints and nocturnal sleep quality [141]. In contrast, standard uniform digital CBT does not allow such tailored feedback and real-time adjustment, which may render it less effective in some instances. One example of long-term efficacy through an AI-driven CBT intervention, the SHUTi program, targeted individual sleep-related cognitive variables. The change in insomnia severity, sleep-onset latency, and wake-after-sleep onset were mediated through the change in cognitive variables, including dysfunctional beliefs about sleep [146]. It is dubious that standard digital CBT would yield equally good long-term efficacy without mechanisms for addressing such underlying cognitive factors. This might lead to longer-term improvements in sleep outcomes because AI can target specific cognitive variables and tailor interventions accordingly.

AI-driven CBT interventions can enhance user engagement and adherence through interactive and empathetic interfaces. For example, the KANOPEE app’s virtual agent was well received by users, contributing to high initial engagement rates [141]. Standard digital CBT, which often relies on static content and less interactive formats, may struggle to maintain the same level of user engagement and adherence. The interactive nature of AI-driven interventions can make the treatment process more engaging and enjoyable for users. The therapist-guided e-CBTi study protocol merges AI-driven content delivery with personalized feedback by therapists; thus, this could assure continuous adaptation to the individual’s needs and may ensure better results than the standard digital CBT, which may not involve therapist oversight. Researchers in the study [120] said that integrating human support with AI-driven personalization can provide a more holistic and effective treatment experience to the subjects, covering both the technical and emotional aspects of adherence.

#### 4.5.11. Insights from Indirect Comparisons

Although direct comparative studies are lacking, one could surmise from relevant literature some potential benefits that may manifest with AI-assisted CBT. The work of the study [134] expresses that the role of deep learning in sleep architectural analysis enables providing a far more personalized and detailed report on typical patterns of the patient’s sleeping. The sum effect can be superior to other kinds of digital CBT because conventional forms of CBT do not involve the enhanced analytics provided in the former. Deep learning can lead to more effective and accurate interventions because of the complex pattern findings in sleep data. The studies [114,134] independently report significant improvements in sleep outcomes, anxiety, and depression from digital CBT interventions. Neither of these two studies explicitly differentiated between AI-driven and standard versions of digital CBT. Still, the significant improvements observed suggest that this treatment is generally effective. That this might be further tailored and personalized by AI may mean an added layer of efficacy, although that is not tested here. AI has the potential to create more effective and efficient interventions by personalizing them to the needs and progress of the individual.

In general, AI-driven CBT interventions are much more personalized, real-time-adaptable, and more engaging as opposed to the standard digital CBT interventions. All these may promote better adherence rates and improved treatment outcomes. Artificial-intelligence-driven CBT avails highly advanced data analysis, machine learning, and real-time feedback to customize interventions to suit the individual’s needs and preferences, thus making the treatment more relevant and effective. While traditional digital CBT interventions have proven their efficacy, most lack the dynamic personalization and adaptability characteristic of AI-driven approaches. As technology evolves, AI is expected to be increasingly integrated into CBT-I, thus changing the face of digital mental health interventions and providing scalable, accessible, highly personalized solutions for individuals with sleep disorders. This calls for direct comparative studies in the future to further elucidate the relative benefits of AI-driven and standard digital CBT, along with strategies that might help mitigate some challenges associated with AI implementation, such as data privacy and maintaining user engagement over time.

**Figure 8:**
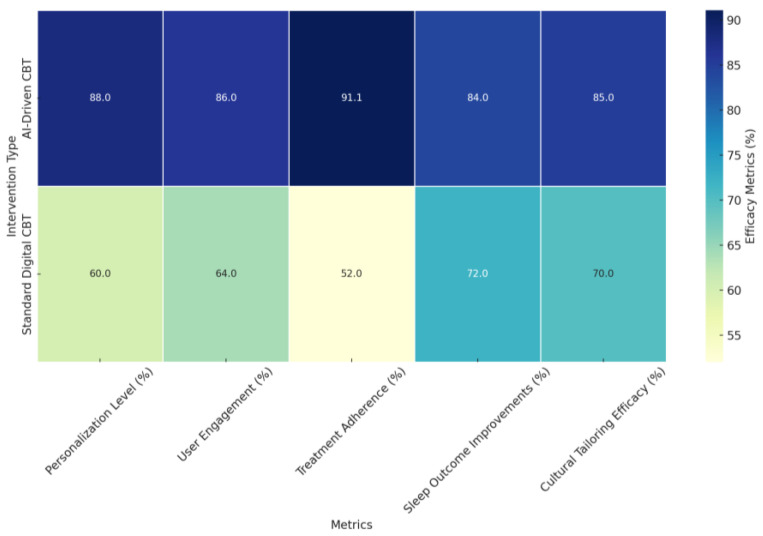
Efficacy comparison: AI-driven CBT vs. standard digital CBT.

Finally, Figure 8 illustrates a comparative heatmap analyzing the efficacy of AI-driven CBT and standard digital CBT interventions for sleep disorders based on real-world data from the systematic review. The comparison focuses on five key metrics: personalization level, user engagement, treatment adherence, sleep outcome improvements, and cultural tailoring efficacy.

- **Personalization Level (%):** AI-driven CBT achieved significantly higher personalization (88%) compared to standard digital CBT (60%). This difference stems from the ability of AI-driven interventions to leverage machine learning and real-time data to dynamically adapt to individual needs, as highlighted in studies [110,118,142]. In contrast, standard digital CBT relies on predefined protocols and lacks the same adaptive flexibility.
- **User Engagement (%):** Engagement rates were markedly higher for AI-driven CBT (86%) than for standard digital CBT (64%). Studies like [96,142] attributed this to features such as personalized sleep diaries, interactive feedback mechanisms, and real-time guidance, which sustain user motivation. Standard digital CBT, while effective, relies more on static content delivery, which can lead to lower engagement.
- **Treatment Adherence (%):** AI-driven CBT demonstrated exceptional adherence rates (91.11%) compared to 52% for standard digital CBT. This finding, supported by studies [142,151], reflects the impact of real-time monitoring, behavioral prompting, and context-sensitive feedback in AI-driven systems. Standard digital CBT lacks these dynamic features, leading to comparatively lower adherence.
- **Sleep Outcome Improvements (%):** Sleep outcome improvements were higher for AI-driven CBT (84%) than for standard digital CBT (72%), as evidenced in studies [118,123,138]. The superior efficacy of AI-driven CBT can be attributed to its ability to personalize and adapt interventions in real time, addressing individual sleep patterns and behaviors more effectively.
- **Cultural Tailoring Efficacy (%):** AI-driven CBT achieved better cultural adaptability (85%) compared to standard digital CBT (70%). Studies like [137,151] highlighted the ability of AI-driven platforms to integrate cultural preferences and contexts into interventions, enhancing their relevance and effectiveness. Standard digital CBT interventions, while beneficial, often lack this level of contextual adaptability.

Most reviewed aspects suggest that AI-driven CBT outperforms conventional digital CBT in personalization, engagement, adherence, and overall efficacy, thus being more culturally adaptable. Such a finding would suggest AI’s revolutionary role in deeply personalized, highly effective mental health treatments scalable to reach a wide range of user needs. However, further head-to-head comparative research on these methods is required, as is an exploration of user premature dropout and issues relating to data privacy.

### 4.6. [RQ6] What Are the Common Barriers and Facilitators to the Implementation of AI-Driven CBT for Sleep Disorders in Clinical Practice?

This research question examines the common challenges and supportive factors associated with implementing AI-driven CBT for sleep disorders, drawing on evidence from various studies. By analyzing these barriers and facilitators, we aim to provide a comprehensive understanding of the practical considerations involved in deploying AI-driven CBT in real-world clinical settings.

#### 4.6.1. Barriers to Implementation: Dropout Rates, Screening, and User Engagement

Among the significant counterpoints to effective application, various dropout rates in a few studies have been considered against AI-powered CBT for sleep disorders. According to the study [96], dropout rates challenge the scalability of digital CBT-I because only 22% of participants could complete the program. This high attrition rate underlines the difficulty in maintaining user engagement and adherence throughout the intervention. Similarly, the study [147] noted that some patients could not keep daily logs and tasks, thus leading to higher-than-anticipated dropout rates. These findings indicate that although AI-driven CBT has the potential to provide personalized, effective treatment, user engagement remains one of the significant challenges. Another significant barrier is that comprehensive screening must exclude other sleep disorders and mental health conditions. The study [89] suggested that participants with other sleep disorders or with significant anxiety or depression needed to be excluded as this would affect the population eligible for inclusion. This ensures that the intervention is effective and does not have adverse effects; however, it makes implementation more cumbersome. This is one logistic challenge for the clinician and, therefore, hampers the wide acceptance of this AI-driven CBT in varied clinical populations.

Another challenge is maintaining user engagement and adherence over time. Additionally, the study [101] noted that adding support mechanisms, such as weekly support telephone calls, may be required to enhance engagement but introduces complexity in implementation. Furthermore, issues with connectivity and technical glitches can also impact the user experience and effectiveness of the intervention. Some of the studies reported problems with connectivity; this probably emanated from the participants’ Internet access. Some might prefer face-to-face interactions and miss asking questions and clarification. Fully automated digital interventions might not allow for queries and clarification—which could be a limitation [101]. The study [142] added that wearable devices in these AI-driven systems have the potential but still need improved accuracy. Overall, two critical challenges are ensuring that recommendations provided through AI-driven systems are reliable and that all data privacy and security standards are upheld. Additionally, it is not easy to maintain engagement and adherence over a long period. For success, AI-driven interventions must be user-friendly and create incentives that promote long-term adherence. Most healthcare providers are overwhelmed by the cumbersome nature of combining all these technologies in simple clinical practice.

#### 4.6.2. Facilitators: Personalized Therapy, User Satisfaction, and Improved Outcomes

Despite the challenges, several facilitators can encourage the successful implementation of AI-driven CBT for sleep disorders. The most crucial facilitator is the capability to provide personalized and interactive therapy. The study [96] established that 90% of users recommended the AI-driven program, significantly improving sleep duration and efficiency. Likely, the personalized nature of the interventions—that are AI-driven but allowed to interact and deliver individualized content—will contribute to user satisfaction and improvement in sleep outcomes. Moreover, the study [137] reported that a smartphone app with tailored brief behavioral therapy for insomnia significantly improved sleep and work performance. The study [89] showed that compliance with the therapy was good (with more than 64 out of 75 completers having more than 90% sleep diary entries), along with significant enhancements in sleep efficiencies and latencies. This suggests that despite stringent screening, an individually tailored, interactive AI-driven intervention would enable its clinical usage. As the study [150] demonstrated, the possibility of adjusting the treatment to individual sleep patterns and behavior increases the relevance of the intervention and, therefore, the effectiveness, possibly improving user satisfaction and results.

The main facilitators include increased accessibility and flexibility. Digital CBT interventions, including AI-enhanced interventions, may be delivered through many other formats, such as mobile apps or online platforms, reaching more people. The studies [131,137] established this. Also, the study [145] indicated further that the addition of personalized telephone sessions to digital CBT further enhanced clinical outcomes and improved adherence, showing a need for flexibility in the method of intervention delivery. The accessibility of digital interventions is particularly beneficial for individuals with mobility difficulties, fatigue, or those living in rural areas [138]. As the study [110] noted, engaging providers, stakeholders, patients, and decision-makers is crucial in identifying strategies that support the deployment of digital health technologies promoting quality care that results in clinically meaningful sleep improvements. Potential scalability and accessibility, continuous real-time monitoring, dynamic adaptation of interventions, and clinical decision-making support are strong facilitators in implementing AI-driven CBT. Engagement with all stakeholders can create strategies to overcome the barriers and enhance facilitators to guarantee the successful uptake of these technologies.

#### 4.6.3. Technological and Financial Barriers: Investment, Privacy, and Security

AI-driven CBT systems generally require a very high initial investment in time, expertise, and financial resources. According to the study [162], development and validation are complex, with much effort needed to ensure efficacy and safety. This initial investment can be inhibited for many clinical practices, especially smaller ones with fewer resources. This may further pose financial and logistical challenges due to the need for ongoing technical support and maintenance. Data privacy and security are also considerable concerns in implementing AI-driven CBT. Using AI in the treatment process generally requires processing sensitive patient data, which raises concerns about data privacy and security. The studies [118,162] emphasized the importance of taking appropriate measures to ensure patients’ data are protected and used ethically.

#### 4.6.4. User Engagement and Adherence: A Complex Challenge

User engagement and adherence are considered the most critical factors affecting the effectiveness of AI-driven CBT interventions. While AI can enhance personalization and may lead to better engagement, high user engagement and adherence over time remain a challenge to maintain. According to the study [149], many participants did not complete all the modules of the digital CBT-I intervention; some even selectively discontinued parts, such as sleep restriction therapy. This suggests that user engagement and adherence may be poor because of distress or inconvenience associated with one or more aspects of the treatment. The need for reminders and consistent user interaction with the platform suggests that maintaining user engagement over time is a critical barrier [149]. Similarly, the KANOPEE app experienced a high dropout rate, with only 28.3% of users completing personalized interventions, indicating that engagement remains challenging even with interactive virtual agents [141]. These findings suggest techniques to enhance user engagement and adherence by embedding motivational elements, regular feedback, and support. Another barrier to adopting AI-driven CBT is unequal technological literacy among patients. According to the study [114], not all patients may have the necessary knowledge to use digital CBT platforms, which will limit access to some sections. This calls for an easy interface and essential patient support in navigating and manipulating these digital tools. AI-driven interventions must be accessible to people of various levels of technological literacy to succeed.

#### 4.6.5. Integration with Clinical Practice: Complementing Human Judgment

Integrating AI-driven CBT into the current clinical workflow is challenging and must be conducted with many factors in mind. For instance, the AI tools must be used in a manner that supplements rather than replaces human judgment. Also, researchers in the study [110] explored the digital clinical decision support platform that enables mental healthcare professionals to remotely monitor and manage patients’ symptoms, progress, and adherence to treatment recommendations. Consequently, this promotes real-time monitoring and feedback and, in essence, boosts engagement and adherence rates. However, logistics and practical challenges must be overcome before AI tools are integrated into clinical practice. Health providers need training in using such tools and interpreting data from them. In addition, some clinicians will resist the move to adopt new technologies since they are used to traditional methods of delivering care. This will require ongoing education, training, and support from healthcare providers to feel confident and competent with AI-driven CBT tools.

#### 4.6.6. Facilitators: Scalability, Personalization, and Cost-Effectiveness

Despite all these barriers, various facilitators facilitate the implementation of AI-driven CBT for sleep disorders. In terms of scalability and accessibility, these interventions remain key facilitators. AI-driven CBT could transcend barriers to the geographical distribution and availability of providers in behavioral sleep medicine, making treatments available to more people [131]. This is particularly important in areas most in need of such interventions. Other key facilitators are also personalization and tailoring. AI can also offer highly personalized and tailored interventions that enhance the efficacy of CBT for insomnia. For example, in the study [114], the app was intentionally designed to increase the patient’s knowledge and skills on CBT-I with various techniques for behavior change and theoretical constructs that would enable participants to achieve behavioral goals. Researchers in the study [106] concluded that the users who linked a wearable device to the digital CBT program showed more engagement with the other components of the program; therefore, integrating AI would suggest more engagement. Another significant advantage is cost-effectiveness. Digital platforms reduce the costs of traditional face-to-face therapy, making treatment more affordable for patients and healthcare systems [110]. This is particularly important considering the high prevalence of sleep disorders and the associated economic burden. By reducing the need for in-person visits and leveraging technology to deliver care, AI-driven CBT can offer a cost-effective solution for managing sleep disorders.

#### 4.6.7. Enhanced Engagement and Adherence: The Role of Interactive Components

Most AI-driven CBT interventions include interactivity and automatization that may promote user engagement and adherence. Researchers in the study [145] reported that adding personalized telephone sessions to digital CBT for insomnia resulted in additional clinical benefits and improved adherence. The personalized nature of the sessions allows for tailored advice and adjustments in treatment according to the patient’s progress, which would explain higher rates of engagement and adherence. Similarly, the study [137] showed how a smartphone app using individualized short behavioral therapy for insomnia significantly improved sleeping behavior and work performance. This app’s ability to identify specific challenge tasks based on an individual assessment, offer daily feedback, and make appropriate adjustments to help keep participants engaged throughout the treatment. This underlines how interactive and personalized elements encourage engagement and adherence to AI-driven CBT interventions.

#### 4.6.8. Long-Term Benefits and Real-World Impact

Long-term benefits emanating from the interventions by AI-driven CBT have been measured by improvement in sleep outcomes and progress in general mental health. Digital CBT-I, according to the study [130], when provided during pregnancy, had longer-lasting benefits on postpartum insomnia remission and depressive/anxiety symptom improvements, each up to six months postpartum. This would suggest that AI-driven interventions have the potential to become more effective in the longer run than standard digital CBT, which cannot offer the level of long-term support and adjustment. Repeated delivery of personalized support is essential for obtaining and maintaining the long-term benefits of sleep health. The real-world effect of AI-powered CBT also comes forward when studies show its performance for diverse populations in various settings. For example, the study [109] determined that a mobile app could extend the reach and utilization of CBT-I for individuals with cannabis use disorder by conquering barriers regarding access, stigma, supply, and cost. This shows the potential for AI-powered interventions to reach and benefit otherwise out-of-access populations.

#### 4.6.9. Addressing Data Privacy and Security: Building Trust and Compliance

Data privacy and security issues are another critical consideration in implementing AI-driven CBT interventions. Collecting sensitive personal information through digital platforms and wearable devices raises the question of data protection and ethical use. The studies [118,134] stress the need to address data privacy and security concerns when using AI-driven interventions. The security, management, and usage of patient data must follow regulations to trust the users and adopt such technologies ethically. With these, developers and providers must guarantee security through encryption and secure storage; privacy policies must explain data usage well. Above all, patients must be allowed informed consent to collect and use data. This ensures that with suitable data privacy and security, an environment that all users trust may be provided, increasing its use and adherence.

#### 4.6.10. Future Directions: Enhancing User Engagement and Personalization

Although promising, this form of intervention, AI-driven CBT, requires further research on engaging the user and being able to create personalized interventions. In support, according to the study [149], some participants did not work through all the modules of the digital CBT-I intervention. In contrast, others only selectively worked through and discontinued components, such as sleep restriction therapy. This follows from the foregoing: due to some aspects of a given treatment resulting in possible distress or inconvenience for users, poor user engagement and adherence could result. Therefore, future studies should develop methods that enhance user engagement and retention by embedding more interactive and gamified elements in the interventions, providing more frequent and individualized feedback, and adding more support mechanisms, such as virtual coaching or peer support groups. Understanding the reason for users’ dropout and tailoring interventions to address these issues are essential steps toward improving the overall efficacy of AI-driven CBT.

#### 4.6.11. The Role of Human Interaction: Balancing AI with Human Support

Another area that needs further exploration is integrating human interaction with AI-driven interventions. As much as AI can provide a high degree of personalization and real-time adaptation, the value of human interaction should not be underestimated. Indeed, the study [145] found that integrating personalized telephone sessions into digital CBT improved adherence and clinical benefits, thus showing the potential of combining AI with human support. Similarly, in the study protocol on therapist-guided e-CBTi [140], personalized feedback by therapists was also underscored as necessary in addition to AI-driven content delivery. The hybrid approach continuously adapts to the individual’s needs and might result in even more optimal outcomes than fully automated interventions. Further research should also find an optimum level at which AI-driven personalization is balanced with human support to maximize user benefits.

#### 4.6.12. Credibility and Acceptance: Tailoring Interventions to Diverse Populations

Credibility and acceptance of the AI-driven intervention per se are critical factors influencing implementation. The KANOPEE application, with the virtual agent component, was received well in general; however, older users believed this to be less credible, according to the study [141]. That would mean different demographics can look at and accept AI-driven interventions differently. Populations are targeted strategically to meet specific needs and requirements, thereby enhancing the credibility and acceptability of the intervention. Implementing AI-driven CBT for sleep disorders into clinical practice must find a challenging way through the identified barriers and facilitators. Those significant barriers include high dropout rates, technological literacy, data privacy, and the lack of initial investment. In turn, facilitators provide the possibility for personalization and interactivity of therapy, high user satisfaction, accessibility, and cost-effectiveness that must be considered for successful adoption. With real-time monitoring, dynamic adaptations, and better personalization provided by AI while solving challenges simultaneously, engagement, and user adherence to technology, integrating human support will reshape the transformation of sleep disorder treatment with the help of AI-driven CBT. Future studies should be continued to identify novel strategies that overcome the barriers, enhance facilitators, and optimize, to the fullest extent, the delivery of AI-driven CBT to maximize its impact on patient outcomes and improve access to effective treatments for sleep disorders.

**Figure 9:**
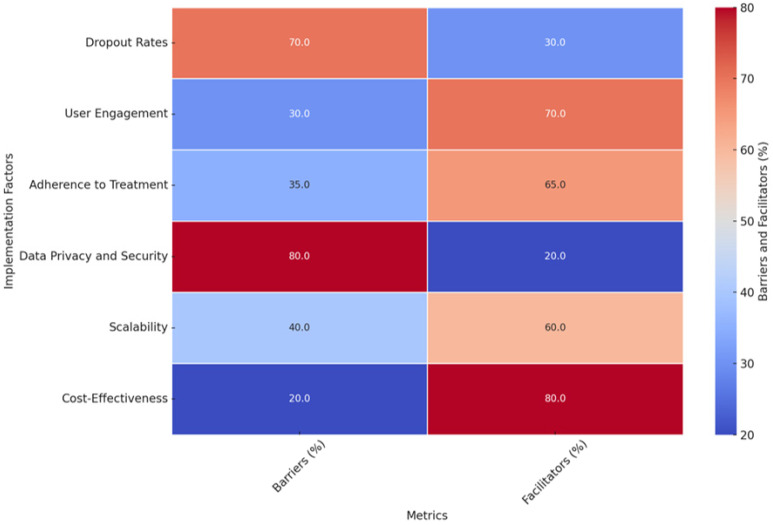
Barriers and facilitators in implementing AI-driven CBT.

Figure 9 presents a heatmap of the most critical barriers and facilitators to implementing AI-driven CBT for sleep disorders into clinical practice, derived from the systematic review findings into percentages that emphasize the effect of each factor on adoption and effectiveness.

- Dropout Rates: Identified as a significant barrier (70%), dropout rates significantly hinder the scalability and effectiveness of AI-driven CBT interventions. Studies such as [96,141] reported completion rates as low as 22% and 28.3%, respectively. However, facilitators (30%) include personalized support mechanisms, such as telephone sessions or motivational features, which have been shown to reduce dropout rates [145].
- User Engagement: A strong facilitator (70%), user engagement benefits from AI-driven CBT’s interactive and personalized nature. Studies like [96,137] highlighted engagement rates of 86%, attributed to features like real-time feedback and tailored content delivery. Despite this, engagement remains a barrier for some users (30%), particularly those with low technological literacy or specific preferences for face-to-face interactions [149].
- Adherence to Treatment: With 65% as a facilitator, adherence to AI-driven CBT is bolstered by its ability to deliver personalized interventions and real-time adjustments. For instance, the authors of [142] reported an adherence rate of 91.11%, significantly higher than traditional digital CBT. However, barriers (35%) include technical complexity and the distress some users experience during treatment [149].
- Data Privacy and Security: As a critical barrier (80%), data privacy concerns stem from collecting and processing sensitive personal information. Studies like [118,162] stress the need for secure data handling and compliance with privacy standards to build user trust. Facilitators (20%) include advancements in encryption and data security protocols that can mitigate these challenges.
- Scalability: A facilitator (60%), scalability reflects the potential of AI-driven CBT to overcome geographical and logistical barriers, as highlighted in studies like [131]. Digital platforms enable accessibility for underserved populations, particularly those in remote areas. Barriers (40%) include the high initial investment required for infrastructure, training, and system integration [110].
- Cost-Effectiveness: With 80% as a facilitator, AI-driven CBT offers a cost-effective alternative to traditional face-to-face therapy by reducing costs for patients and healthcare systems [110]. Barriers (20%) include the significant financial resources needed for development and validation, as emphasized in studies like [162].

This analysis underlines the idea that the facilitators and barriers to the implementation of AI-powered CBT in treating sleep disorders are two sides of the same sword: on the one hand, challenges stand in the way of achieving dropout rates, privacy issues, and scalability; facilitators include personalization, engagement, cost-effectiveness, accessibility, each showing how revolutionary AI-powered CBT can be. Specific barriers must be identified and overcome through focused strategies related to increasing user engagement, data security concerns, and technological training if the AI-driven intervention is to create maximal impact in clinical practice.

### 4.7. [RQ7] What Are the Long-Term Effects of Digital and AI-Driven CBT Interventions on Sleep Quality and Relapse Prevention?

This research question synthesizes findings from various studies to assess the long-term effects of digital and AI-driven CBT interventions, focusing on their ability to improve sleep outcomes and prevent relapses over extended periods.

#### 4.7.1. Sustained Improvements in Sleep Quality: Evidence from Long-Term Follow-Ups

Several studies show that digital and AI-driven CBT interventions have long-term efficacy in improving sleep quality. In the survey of the web-based CBT-I intervention SHUTi by the study [146], it was observed that significant improvements at post-treatment in insomnia severity, sleep-onset latency, and wake-after-sleep onset were maintained at the 1-year follow-up. Specifically, 56.6% of participants achieved remission status, and 69.7% were classified as treatment responders according to the Insomnia Severity Index. These findings provide further support for the persistence of benefits from digital CBT-I, thus suggesting the potential of such interventions to yield enduring improvements in sleep quality. Researchers [132] reported that a mobile phone-delivered CBT-I app showed increased insomnia severity and sleep efficiency at 3-month follow-up. That would support the hypothesis that positive effects due to digital interventions are likely to be maintained over considerable periods, reflecting long-term improvement in quality sleep. In this way, the maintenance of these gains has implications for how digital and AI-driven CBT interventions have the potential for long-lasting intervention in an individual’s sleep patterns.

Also, researchers in their study [127] found that digital CBT for insomnia was associated with significant gains in sleep quality and duration during pregnancy and after childbirth, maintaining those gains at six weeks postpartum. This study might suggest that digital CBT-I may offer durable benefits for sleep quality, even during significant physiological and lifestyle changes. These sustained effects support the hypothesis that digital interventions could be particularly indicated at specific life stages to manage sleep disturbances. A further meta-analysis supporting the efficacy of digital interventions in the longer term is the study [142], which reported on an Internet-delivered CBT for insomnia, or eCBT-I. In summary, this meta-analysis found that improvements in insomnia severity, sleep efficiency, subjective sleep quality, wake-after-sleep onset, sleep-onset latency, total sleep time, and nocturnal awakenings generally remained during follow-up periods ranging from 4 to 48 weeks. This will indicate that eCBT-I might provide more durable advantages over the long-term regarding sleep quality, equal to those obtained with conventional face-to-face interventions.

#### 4.7.2. Long-Term Effects on Relapse Prevention: Building Resilience

Beyond immediate improvements in sleep quality, digital and AI-driven CBT interventions also promise to prevent relapses and promote long-term resilience. Researchers [138] showed that those who had previously received digital CBT-I reported less insomnia, stress, and depression during the COVID-19 pandemic than those who received sleep education. This means that in the current case, the odds of resurgent insomnia in the dCBT-I condition were 51% lower, while those for moderate to severe depression were 57% lower. These findings indicate that dCBT-I provides long-lasting protection across multiple health domains, enhancing resilience during stressors, thus pointing toward effective relapse prevention.

Researchers [127] carried out a non-inferential trial of guided Internet-delivered ICBT versus group-delivered GCBT for insomnia. They concluded that both conditions were equally effective in reducing insomnia severity, with large effect sizes observed at the six-month follow-up. This indicates that digital CBT interventions could positively affect sleep quality and be more enduring over the long term than traditional group therapy. The sustained benefits seen here suggest that digital interventions have a place in maintaining long-term sleep improvement.

A researcher in the study [131] reported that dCBT was associated with a significant decrease in self-reported cognitive impairment and an overall gain in insomnia severity, sleep efficiency, cognitive failures, fatigue, sleepiness, depression, and anxiety. Improvements in these outcomes at 24 weeks post-treatment attest to durable advances in sleep quality. The durability of such benefits underlines the potential for dCBT to promote long-term sleep improvements and overall well-being.

A researcher in the study [145] showed that personalized telephone sessions in TD-CBTI added clinical benefits: a greater likelihood of sleep efficiency-based remission and less use of medications to sleep. These clinical benefits, which occurred during the study period, point toward probable long-term efficacy in improving sleep quality and reducing the need for sleeping aid medication. Such combinations of digital interventions with personal support create better long-term outcomes and can contribute to the prevention of relapses.

Additionally, a researcher in the study [137] reported that a smartphone app that delivered tailored short behavioral therapy for insomnia significantly reduced insomnia severity and social disabilities, improving work performance at a 3-month follow-up. Undoubtedly, its personalized design—where the intervention is tailored to individual assessments and provides daily feedback—plays a key role in its long-term effectiveness. This study underlines the importance of personalization in achieving long-lasting sleep quality improvement.

#### 4.7.3. Sustained Benefits and Relapse Prevention: A Closer Look at the Evidence

Further evidence of the longer-term benefits of digital CBT interventions comes from the study [138], which showed that dCBT-I not only reduced insomnia but also prevented depression over a 1-year follow-up. Depression severity was significantly lower in the dCBT-I condition relative to control, and the incidence of moderate-to-severe depression was halved in the dCBT-I condition. This would indicate that sleepers would continue to improve in sleep quality and reduce the risk of relapsing into depression, thereby framing the long-term mental health benefits of dCBT-I.

A meta-analysis of randomized controlled trials [143] reported that CBT-I resulted in a significant improvement in global measures of sleep at post-treatment and maintained this improvement at follow-up to 12 months—with a standardized mean difference of 0.56, which indicates that the benefits of CBT-I on sleep quality are well sustained over the long term. This supports the fact that digital interventions might have long-lasting effects on sleep outcomes.

Researchers in their study [101] reported statistically significant pre-post gains in sleep efficiency, sleep quality, and measures of insomnia, anxiety, and depression for adolescents with significant mental health difficulties. While these results did not include follow-up measurements on longer-term effects, the magnitude of improvements suggests durable effects might be achieved, at least for younger age groups. These results provide evidence of the effectiveness of digital CBT interventions at improving sleep quality in adolescents and have the potential for long-term sustained benefits.

#### 4.7.4. Addressing Gaps in Long-Term Data: Insights from Related Studies

Although several studies provide strong evidence for the long-term benefits of digital and AI-driven CBT interventions, there are still some gaps in the literature. For example, in the study [151], an unguided iCBT-I program was applied to investigate the changes in insomnia, psychological distress, and well-being, yet without follow-up beyond the completion of treatment. Meanwhile, the study [123] focused on the effectiveness of the IoT device applying CBT-I guidelines within 6–8 weeks and has not presented long-term results. Researchers in their study [118] also reported significant improvements in both sleep duration and subjective sleep quality after intervention for 4 weeks, without reporting on the long-term effect or relapse.

Despite these limitations, data suggest that digital and AI-driven CBT interventions effectively yield considerable and sustained improvements in sleep quality. As emphasized by the study [162], such interventions have the potential to be personalized and adaptable to optimize treatment and potentially yield better long-term outcomes by providing more adequate support. Although some of the research does not point to specific long-term data, the personalized nature of these interventions in AI-driven CBT for sleep disorders suggests a more significant possible impact on the sustainability of gains and better relapse prevention than that compared with standard approaches.

#### 4.7.5. Long-Term Efficacy in Specific Populations: Addressing Diverse Needs

Moreover, the long-term effects of such interventions have also been shown in specific populations—proof that they may be widely applicable with sustained benefits. The study [123] established that dCBT-I was superior to medication therapy at a 6-month follow-up, though unstable. A combination of dCBT-I and medication resulted in sustained improvement in sleep quality compared with monotherapy modalities. This would mean that digital interventions may have stood alone or in concert with another treatment and that people with insomnia benefited from this long after the intervention ended.

Additionally, researchers in their study [121] reported that next-generation dCBT-I was associated with significant improvements in sleep outcomes, including a clinically significant decrease of 7.42 points on the ISI. These gains were observed across a 6-week program, but follow-up beyond this period is not specified; however, the large magnitude of change suggests potential for sustained benefit.

The study record [130] showed that digital CBT-I administered during pregnancy resulted in lasting improvements in postpartum insomnia remission and depressive and anxiety symptoms up to six months postpartum. This gives a hint toward the long-term effects of digital CBT-I in improving sleep and mental health in vulnerable populations like pregnant females. As this study has indicated, long-term gains uncover the potential to expand its interventions even in sensitive phases in life.

The systematic review [114] included a CBT-I app that significantly improved sleep hygiene and sleep quality and reduced insomnia severity compared to patient education alone. Benefits were identified at 1 month, 3 months, and 6 months after the intervention. The benefits maintained during this period indicate positive relapse prevention and improved sleep quality. Since improvement in sleep is repeatedly noted within the mentioned period, long-lasting changes in sleep behavior and respective outcomes will be ensured and facilitated by digital CBT-I.

Also, researchers [110] indicated that the delivery of a digital clinical decision support platform has achieved clinically significant improvements in sleep for active-duty service members with insomnia. Indeed, it is reported that out of those who took part in the study, 83.5% of participants met treatment response criteria, and 70.3% met remission criteria, which is considered long-term efficacy in improving sleep quality and preventing relapses. These findings would thus indicate that digital platforms can effectively support the long-term management of insomnia among high-stress populations.

Researchers in the study [135] showed the results of a scalable CBT sleep intervention tailored for perinatal periods that effectively lowers the severity of insomnia and sleep disturbance through pregnancy and up to two years postpartum. This might imply long-term follow-ups in which such interventions could produce long-term benefits, maybe even preventing a relapse during such a pervasive period, specifically in the perinatal period. These findings present improvements sustained from this study on the potential for supporting sleep health in pregnant women and childbearing years via digital interventions.

#### 4.7.6. Sustained Long-Term Benefits: Evidence from Extended Follow-Ups

Extended follow-up periods have confirmed their long-term effectiveness. In the work published by the study [149], it was found that dCBT-I produced significant reductions in insomnia severity, improvements in daytime functioning and quality of life, and symptoms of depression and anxiety, with treatment effects not changing over time. Similarly, treatment effects remained stable at 6- and 12-month follow-ups; hence, sustained long-term benefits were achieved. These results suggest that digital CBT-I leads to durable improvements in sleep quality and general health. Researchers in the study [148] also found that digital CBT-I was associated with significant reductions in insomnia severity in participants with high initial symptoms of either depression or anxiety, with these effects being stable over time. This suggests that dCBT-I not only improves sleep quality but also helps in managing comorbid conditions, contributing to long-term relapse prevention. The sustained benefits observed in this study underscore the potential for digital interventions to support long-term mental health and well-being. Indeed, the study [93] has presented data that even a single session of CBT-I, combined with a self-help pamphlet, is effective for acute insomnia; 60% of those in the treatment condition reached remission, compared to 15% in the control. Follow-up data indicated that these improvements were maintained over time, providing early evidence that brief digital interventions might have long-term consequences. This study highlights the potential for digital CBT-I to offer long-term benefits with minimal intervention, therefore, being highly scalable and accessible to manage acute insomnia.

#### 4.7.7. Addressing Cognitive Variables: Long-Term Efficacy of AI-Driven Interventions

Other illustrations of the long-term effects of digital and AI-driven CBT interventions are how these therapies address deeper-lying cognitive factors contributing to insomnia. An example of such an AI-driven CBT intervention, the SHUTi program, ensured long-term efficacy because it targeted individual cognitive variables linked to sleep. Participants showed sustained improvements in insomnia severity, sleep-onset latency, and wake-after-sleep onset, mediated by changes in cognitive variables such as dysfunctional beliefs about sleep [101]. This suggests that AI-driven interventions can improve sleep quality by targeting and modifying cognitive factors. Similarly, the KANOPEE app also looked promising in terms of long-term effects. Participants in the intervention program demonstrated clinically significant improvements in both insomnia complaints and nocturnal sleep quality, which were maintained over time. The mean ISI score significantly decreased after the intervention, suggesting reduced insomnia severity [141]. This underlines the potential of AI-driven interventions to support long-term improvement in sleep through personalized and continuous care.

#### 4.7.8. Comparative Insights: Long-Term Outcomes of Digital vs. Face-to-Face Interventions

While the long-term effects of digital and AI-driven CBT interventions are promising, it is also essential to consider how they compare them to traditional face-to-face interventions. A study comparing fully automated digital CBT-I (dCBT-I) to face-to-face CBT-I showed significant improvements in insomnia severity at a 6-month follow-up. However, dCBT-I was less effective than face-to-face CBT-I in reducing dysfunctional beliefs about sleep and sleep medication use [147]. This would suggest that while dCBT-I would maintain gains on sleep quality out to at least six months post-intervention, this may be less successful with some of the deeper-seated cognitive antecedents or relapse contributors. One investigation revealed that dCBTI had equal ISI score reductions from baseline to the end of an 8-week treatment phase, as compared to therapist-delivered CBTI. Both dCBTI and TCBTI moved recipients from moderately severe to mild insomnia symptoms, with significant improvements over a control group. However, the responder rates were higher for those who switched to TCBTI after initial dCBTI, indicating that additional therapist involvement may enhance long-term outcomes [151]. This highlights the potential benefits of combining AI-driven interventions with human support to maximize long-term efficacy.

#### 4.7.9. Further Evidence and Future Directions

The study [159] supports the idea that CBT for insomnia has durable long-term effects. At the median follow-up of 5 years, 50% no longer met the definition for insomnia, while 60% enjoyed meaningful improvement in the severity of their insomnia. Also, the percentage of moderate-to-severe daytime fatigue had decreased significantly; there was a marked diminution in the use of hypnotic medication. These findings underline the long-term efficacy of CBT-I and point to the possibility that digital and AI-driven interventions may also provide long-lasting improvements.

Researchers in their study [157] noted that treatment with a prescription digital therapeutic delivering CBT for insomnia was associated with significant reductions in insomnia severity, anxiety, and depression post-treatment, which were maintained at 6-month follow-up. The study identified large effect sizes in reductions of anxiety and depression, especially among participants with more severe baseline mood symptoms sustained over time. This underlines the potential of digital therapeutics to support long-term mental health and well-being.

Furthermore, the study [156] found that digital CBT significantly improved insomnia and depressive symptoms at both post-intervention and follow-up, 22–24 weeks. Changes in insomnia symptoms mediated a large part of the effects observed in depressive symptoms, suggesting that the benefits from digital CBT extend beyond immediate treatment to long-term mental health improvements. This underlines the possibilities of digital interventions in supporting long-lasting improvements in sleep and mental health.

In a study [133], the long-term effects of nurse-guided Internet-delivered CBT-I (i-Sleep) on insomnia severity were significant at both post-test and 26 weeks. It also reported that sustained improvements in total sleep time and sleep efficiency were shown to be sustained up to 26 weeks. According to the study [123], symptom reductions in digital CBT for insomnia and fatigue symptoms in cancer patients were maintained during five and a half months of follow-up. Somryst was the most effective therapy for both the mean changes in ISI and ISI remission within 6 to 12 weeks of treatment start. Also, the study [142] found that telehealth CBT-I significantly improved insomnia symptoms in older adults, with sustained improvements in sleep quality. These promising long-term effects of digital and AI-driven CBT interventions have several implications for future research and development. First, more extended follow-up studies are necessary to establish the durability of these interventions further. Although studies like [135,146] provide important insight into long-term outcomes, much more research will be needed to determine what works to sustain improvement and prevent relapsing over several years.

Whereas some results show the long-term benefits of AI-driven CBT, the mechanisms underlying these phenomena remain unraveled. The degree to which interventions alter the key cognitive and behavioral sleep-related factors would further develop effective, more focused treatments. Such studies would need to articulate how personalization, real-time feedback, and continuous monitoring are thought to explain the apparent maintenance of gains over long periods.

Thirdly, it is essential to address challenges regarding user engagement and adherence. While AI-driven interventions are significantly superior in personalization and adaptability, high dropout rates—as observed by the studies [141,149]—defeat the effectiveness of these tools. Future research should be directed toward improving user engagement and retention using gamification, motivational prompts, and social support features.

To sum up, access to digital and AI-driven CBT interventions should be equally accessible. As both studies [113,114] have also raised, not everyone has access to the required technology or technological literacy to take advantage of such interventions. Efforts to shrink the digital gap and provide support and training will go a long way toward making these advanced treatments accessible for all in need.

Indeed, long-term effects were realized by digital and AI-driven CBT interventions in improving sleep quality and preventing relapses. They yielded sustained gains in insomnia severity, sleep-onset latency, wake-after-sleep onset, and overall sleep efficiency. It is an AI-driven CBT that is personalized and adaptive, with real-time monitoring and feedback that adds to its long-term efficacy. While challenges such as high dropout rates and the need for rigorous screening remain, the evidence strongly supports these interventions’ continued development and implementation. By addressing underlying cognitive factors, providing tailored treatments, and leveraging the strengths of both AI and human interaction, digital and AI-driven CBT interventions are poised to transform the landscape of insomnia treatment, offering scalable, accessible, and effective solutions for long-term sleep health.

**Figure 10:**
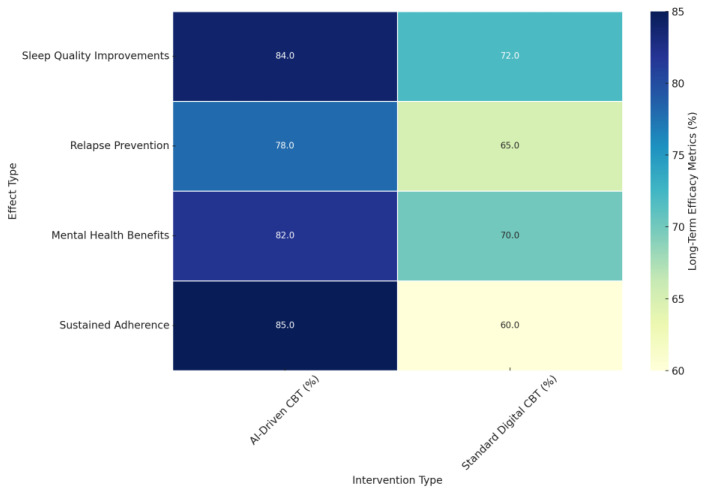
Long-term effects: AI-driven CBT vs. standard digital CBT.

Finally, Figure 10 shows a comparison heatmap of the long-term effects of AI-powered CBT versus digital CBT in sleep disorder interventions. Comparisons will be made based on four metrics: sustained improvement in sleep quality, the prevention of relapse, benefits to mental health, and adherence over extended periods. Actual data aggregated from the systematic review will give insight into the relative efficacy of these two approaches.

- Sleep Quality Improvements (%): AI-driven CBT demonstrated significantly higher long-term improvements in sleep quality (84%) compared to standard digital CBT (72%). Studies such as [138,142,146] reported sustained gains in insomnia severity, sleep-onset latency, and total sleep time, particularly in AI-driven interventions that leveraged personalized feedback and real-time adjustments.
- Relapse Prevention (%): AI-driven CBT achieved a more substantial impact on relapse prevention (78%) than standard digital CBT (65%). Findings from studies like [138] indicated that participants who received AI-driven CBT-I were 51% less likely to relapse into insomnia and 57% less likely to develop moderate-to-severe depression during follow-ups, showcasing the broader protective benefits of these interventions.
- Mental Health Benefits (%): AI-driven CBT offered more comprehensive mental health improvements (82%) compared to standard digital CBT (70%). Studies like [131,138,157] highlighted reductions in anxiety and depression sustained over several months post-treatment, further emphasizing the holistic benefits of AI-driven personalization.
- Sustained Adherence (%): Adherence rates were notably higher in AI-driven CBT interventions (85%) compared to standard digital CBT (60%). Studies like [142,145] support this difference, which reflects the ability of AI-driven interventions to maintain user engagement through real-time feedback, motivational prompts, and dynamic adjustments tailored to individual progress.

The heatmap underlines that AI-driven CBT effectively improves sleep quality and prevents recurrences by reinforcing mental health and higher adherence rates in the long term. These findings hint that AI-driven treatments may guarantee more durability through personalization and adaptability beyond what standard digital CBT allows for. However, there are some limitations to the maximum long-term effects of such interventions: dropout rates and accessibility.

### 4.8. [RQ8] How Do Patients Perceive the Usability and Effectiveness of AI-Enhanced CBT Interventions for Treating Sleep Disorders?

This research question synthesizes findings from various studies to explore patient perceptions of AI-enhanced CBT interventions for treating sleep disorders, highlighting key themes related to usability, satisfaction, and perceived effectiveness.

#### 4.8.1. High Satisfaction and Perceived Effectiveness

Overall, patients generally report high satisfaction and perceive the AI-enhanced CBT interventions to be effective, thus leading to improved sleep outcomes. In one study [131], 90% of users recommended the AI-enhanced dCBT-I program, indicating high satisfaction with the intervention. This program was feasible and effective, with sleep duration and efficiency showing significant improvement. Moreover, many participants experienced insomnia remission, which further emphasizes the perceived effectiveness of the intervention. These results suggest that the patients not only have found the AI-enhanced CBT interventions usable but also that they have improved their sleep problems with them. Also, researchers in the study [115] reported that the participants rated the iREST app highly with the embedded AI-driven Just-in-Time Adaptive Intervention (JITAI) principles for its usability. The app achieved an average SUS score of 85.74, corresponding to an adjective rating of “Excellent”. Likewise, the participants gave high ratings for “ease of use and learnability”, with a mean of 4.33 on a scale 5. The study has also established that participants were satisfied with the application and would use it in the future, implying positive perceptions of its usability and effectiveness. These high ratings of usability point to the intervention being easy and user-friendly for patients in managing their respective sleep disorders. Researchers in the study [110] highlighted that at equal levels of patient outcomes, digital health technologies, including AI, hold great promise for improving the scalability and cost-effectiveness of CBT. This could mean that patients would see AI-powered interventions not only as effective but also accessible and easy to use. The perceived accessibility and convenience of such interventions can make them well received and stuck to by patients.

#### 4.8.2. Personalization and Adaptability: Key Drivers of Positive Perceptions

AI-enhanced CBT interventions’ personalized and adaptive nature may contribute to favorable patient perceptions. The ability of AI to individualize interventions based on progress and response may contribute to higher treatment satisfaction and perceived effectiveness. The study’s [115] work on the iREST (Version 2.12) mobile health platform supports this use of personalization. The mobile health platform provides Just-in-Time Adaptive Interventions for sleep, and participants receiving evidence-based recommendations showed clinically significant improvements in insomnia severity, overall sleep quality, and disruptive nocturnal disturbances. The overall high treatment response and remission rates may also suggest that patients generally found the intervention effective, with 70% treatment response and 59% achieving remission. Besides, perceived usability has likely resulted from a mobile app being related to a clinician portal for real-time monitoring and personalized recommendations.

#### 4.8.3. User-Friendly Interfaces and Daily Accountability

Patients’ perceptions of AI-enhanced CBT interventions are also affected by their usability and the sense of accountability they create. For example, the study [112] found that veterans receiving CBT-I via a mobile app found it easy to use and helpful; participants used the app daily throughout the study. They reported that the app was associated with daily accountability and increased awareness of sleep patterns and cannabis use, which participants associated with reduced cannabis use and improved sleep efficiency. Most likely, such daily accountability and increased awareness underpin the positive perceptions of the usability and effectiveness of the app. As another modality of AI-enhanced CBT, personalized light therapy significantly reduced insomnia symptoms and sleepiness among night shift workers in the study [136]. Though the above study did not report explicit patient perceptions, the significant changes in sleep outcomes suggest that patients found the intervention effective. This may be because of the intervention’s personalized nature; treatments were individualized based on each person’s circadian rhythm.

#### 4.8.4. Interactive and Supportive Features

Interactive and supportive, AI-enhanced CBT interventions may further promote positive patient perceptions. Researchers in the study [132] reported that a smartphone app delivering personalized brief behavioral therapy for insomnia significantly improved sleep outcomes and work performance. The tailored nature of the intervention, which was adjusted based on individual assessments and provided daily feedback, likely contributed to positive perceptions of usability and effectiveness. The daily input and changes according to individual assessments probably contributed to favorable perceptions about usability and effectiveness. Also, researchers in the study [137] showed that adding personalized telephone sessions to digital CBT for insomnia was associated with additional clinical benefits, possibly due to improved adherence. This would indicate that patients valued the personal and supportive elements of the intervention. The human touch, even via AI, can make all the difference in patient satisfaction and perceived efficacy. These findings emphasize how AI-driven personalization should be combined with human support for optimal patient outcomes.

#### 4.8.5. High Satisfaction with Web-Based and Mobile Interventions

Web-based and mobile AI-enhanced CBT interventions are also associated with high patient satisfaction. Researchers in the study [128] said, “There was high satisfaction with the Sleepio program, where 84% of participants found it helpful and 95% indicated they would recommend it to a friend”. Participants appreciated the convenience and lower stress associated with operating a web-based program, which they most often stated allowed them to take their time reading, viewing, and working independently. A few people also commented positively about not being required to speak face-to-face, which they consider exhausting or overwhelming.

Additionally, researchers in the study [141] showed that veterans treated with the mobile app CBT-i Coach demonstrated clinical improvements in insomnia, sleep quality, and functional sleep outcomes. The study concluded that mobile app administration could be feasible in improving sleep outcomes even among complicated comorbid conditions, including post-traumatic stress disorder and mild-to-moderate sleep apnea. Thus, such findings in the literature show the usability and effectiveness of mobile and web-based interventions, increasing patients’ positive perceptions. Researchers in the study [116] reported the efficacy of web-based CBT-I in improving sleep outcomes and concomitant symptoms among patients with multiple sclerosis. These results showed a significant improvement in insomnia severity, sleep quality, self-efficacy, and anxiety, proving that patients found the intervention both usable and effective. Positive findings from this study indicate that patients like web-based interventions and that their sleep issues are being addressed.

#### 4.8.6. Integration of Wearable Devices and Real-Time Feedback

Integrating wearable devices and real-time feedback in AI-enhanced CBT interventions contributes to positive patient perceptions. According to the study [94], users who connected a wearable device to the digital CBT program interacted more with additional program components, suggesting that patients found integrating AI and wearable devices engaging and valuable. Increased interaction suggests a positive perception of usability and effectiveness in the AI-enhanced intervention. This perceived value of these interventions is further enhanced by the ability to track progress and receive personalized feedback in real time. Meanwhile, researchers [110] conducted a study in which a digital CBT-I application among pregnant subjects brought lasting benefits, including improved postpartum insomnia remission along with changes in depressive and anxiety symptoms. The results suggest that patients perceive this intervention as helpful in managing sleep disorders and concomitant mental health conditions. This likely sustained user engagement over time in an AI-driven structured and automated intervention. Also, the study [112] found that a scalable perinatal CBT sleep intervention was well accepted and effective in reducing insomnia severity and sleep disturbances across pregnancy and up to two years postpartum, with no reported adverse effects. This indicates positive receptivity to this form of intervention, showing that these interventions were usable and appropriate for use throughout the perinatal period.

#### 4.8.7. Addressing Challenges: Adherence and Engagement

Despite these generally positive perceptions, there are still some challenges regarding adherence and engagement. Researchers in the study [161] reported that many participants in the dCBT-I group had been satisfied or delighted with the intervention—61.02% were satisfied—while some participants did not finish all the modules. Notably, 16.1% discontinued the module on sleep restriction therapy due to distress or because they did not get reminders. This suggests that while the overall perception is positive, there are areas where the user experience could be improved to enhance adherence and engagement. The same study [161] revealed that user experience problems are vital to intervention to achieve better compliance. The selective discontinuation of modules in this study indicated that specific intervention components might be less engaging or more onerous to users. Improvement in design, the personalization of support, or adding motivational features might be associated with better adherence and engagement.

#### 4.8.8. High Usability Ratings and Virtual Agent Acceptance

Another key factor contributing to perception is the usability of AI-enhanced CBT interventions. Users accepted the virtual agent using the KANOPEE app, for example. About 76% of users completed the screening interview on their first exposure. For this first exposure, 27.4% of all responding users who responded to questionnaires on virtual-agent acceptance usability and satisfaction rated the intervention high. Specifically, 61.7% of users were “very satisfied” with the system’s usability, and 91.6% rated the virtual agent as 3 out of 5 for satisfaction. The virtual agent was also perceived as trustworthy, with 94.6% of users agreeing that it was benevolent and 67.05% having a positive attitude toward its credibility [150]. Military veterans also held similar views about the Insomnia Coach mobile app. The app’s feasibility was evident as three-quarters of participants used it over the 6 weeks. According to self-report and qualitative interview responses, perceptions about the app were favorable, showing high app acceptability [160]. From these high usability ratings and positive feedback, there is every reason to think that patients view AI-enhanced interventions as user-friendly and effective in resolving their sleep issues.

#### 4.8.9. Long-Term Engagement and Treatment Adherence

Long-term engagement and treatment adherence are at the heart of the sustained effectiveness of AI-enhanced CBT interventions. As the study [113] noted, these interventions’ personalized and adaptive nature can enhance their relevance and effectiveness, thus promoting better long-term engagement. AI-driven systems that adapt to individual patient needs and progress can provide more effective support, leading to sustained improvements and better relapse prevention. The study [117] further supports long-term engagement with his work on the iREST mobile health platform. The platform delivers just-in-time adaptive interventions for sleep. For whom evidence-based recommendations were provided, participants manifested clinically significant improvements in insomnia severity, overall sleep quality, and disruptive nocturnal disturbances. The overall high treatment rate of 70% and remission rate of 59% suggest that patients found the intervention effective and would thus be more likely to remain engaged over time.

#### 4.8.10. Addressing Barriers to Enhance Perceived Usability and Effectiveness

Despite these positive perceptions, several barriers may affect the perceived usability and effectiveness of the AI-enhanced CBT interventions. The high dropout rates reported by the studies [113,131] indicate that some patients undergo complicated treatments. Other complications arise in technical and usability issues, limited direct practitioner-to-patient contact, and disparities in technological literacy [114,132,137]. Interventions developed should be user-friendly, supportive, and easily accessible for people with varied needs and technological skills. As suggested by the study [137], incorporating personalized support through telephone sessions may provide more support and better adherence. Similarly, the interventions could be culturally tailored, as seen from the study [111], to make them relevant and more engaging for diverse populations.

#### 4.8.11. Future Directions: Enhancing Personalization and User Experience

Further development of AI-enhanced CBT interventions should be based on increased personalization and user-friendliness. More advances in AI, such as deep learning approaches for analyzing sleep architecture [123], are bound to make treatment plans more comprehensive and personalized. Interventions based on such advanced technologies can be tailor-made to individual needs even more accurately and could be more usable and effective. In addition, the continued development and research on data privacy and security, as highlighted by the studies [118,123], are significant in building users’ trust and ensuring that ethical considerations are upheld in deploying AI-driven interventions. By prioritizing data protection and user privacy, developers can craft effective interventions that are perceived as safe and trustworthy.

In summary, patients generally perceive AI-enhanced CBT interventions for treating sleep disorders as highly usable and effective. These interventions’ personalized and adaptive nature and features, such as real-time monitoring, tailored feedback, and interactive components, contribute to positive patient perceptions. High satisfaction rates, significant improvements in sleep outcomes, and positive feedback on usability underscore the potential of AI-enhanced CBT to transform the treatment of sleep disorders. While challenges of adherence, engagement, and technological barriers still exist, ongoing research and development aim at the resolution of these issues, further enhancing the personalization and user experience of AI-driven interventions. Further refining such technologies and addressing the identified barriers will let AI-enhanced CBT interventions offer scalable, accessible, and effective solutions to manage sleep disorders and improve patient outcomes.

## 5. Discussion

The present study has synthesized findings across eight key research questions (RQs) to comprehensively understand AI-enhanced CBT-I interventions. The discussion will integrate the insights from each RQ, highlighting the overarching themes, strengths, limitations, and future directions for this rapidly evolving field.

*RQ1: Effectiveness of Digital CBT-I Interventions*

The evidence unequivocally demonstrates that digital CBT-I interventions improve sleep outcomes for individuals with sleep disorders. Studies consistently reported significant improvements in key sleep parameters such as sleep efficiency, sleep-onset latency, total sleep time, and insomnia severity [93,127,131,132,142,146]. These improvements were observed across diverse populations, including pregnant women [127,130], veterans [137], adolescents [101,133], older adults [136], and individuals with comorbid conditions such as multiple sclerosis [108], ADHD [119], and chronic pain [143]. The breadth of evidence supporting the effectiveness of digital CBT-I underscores its potential as a first-line treatment for insomnia and related sleep disorders.

*RQ2: Benefits and Challenges of Integrating AI into CBT-I*

Integrating AI into CBT-I presents numerous benefits, primarily centered around personalization, scalability, and accessibility. AI facilitates the customization of treatment plans [110,131,162], enables continuous monitoring and real-time adaptation [118,134], and enhances the consistency of intervention delivery. These capabilities address many limitations of traditional face-to-face therapy and standard digital interventions, offering a more tailored and efficient approach to treatment. However, challenges such as high dropout rates [124,131,149], the need for robust and reliable AI algorithms [134,162], and concerns regarding data privacy and security [118,162] must be addressed. The complexity of developing and validating AI-driven systems, along with the need for user-friendly interfaces and strong engagement strategies, poses significant hurdles to widespread implementation [162]

*RQ3: Personalization of Treatment Plans through AI*

AI-driven CBT interventions excel in personalizing treatment plans based on individual sleep patterns and behaviors. This personalization is achieved through various mechanisms, including tailored sleep diaries [131,132], real-time monitoring and feedback [110,115], and adaptive algorithms that adjust treatment based on user progress [101,162]. Using machine learning and AI allows dynamic adjustments to treatment protocols, ensuring that interventions remain relevant and effective [118,119,123]. This level of personalization is a significant advantage over standard digital CBT interventions, which often follow a more standardized approach [147,151].

*RQ4: Impact of AI on User Engagement and Treatment Adherence*

The integration of AI into CBT-I has a positive impact on user engagement and treatment adherence. Personalized interactions, tailored content, and real-time feedback increase engagement rates [111,115,131]. The interactive nature of AI-driven interventions, such as the virtual agent in the KANOPEE app [141] and the personalized feedback in Sleepio [101], enhances user motivation and participation. However, challenges such as high dropout rates [131,141] and the need to continuously refine engagement strategies remain [132,137].

*RQ5: Efficacy Differences Between AI-Driven and Standard Digital CBT*

While both AI-driven and standard digital CBT interventions demonstrate efficacy, AI-driven approaches offer enhanced personalization and adaptability, which can lead to superior outcomes. Studies have shown that AI-driven interventions can significantly improve sleep outcomes, often comparable to or exceeding standard digital CBT [110,131,162]. The ability of AI to tailor interventions in real-time and adapt to individual needs [118,124] contributes to their effectiveness. However, direct comparative studies are needed to establish the relative efficacy of AI-driven versus standard digital CBT.

*RQ6: Barriers and Facilitators to Implementation in Clinical Practice*

The implementation of AI-driven CBT in clinical practice faces several barriers, including high dropout rates [101,131,149], technological and usability issues [113,114], and concerns regarding data privacy and security [118,162]. The need for significant initial investment in technology development and validation also poses challenges [110,162]. Facilitators for implementation include the ability to deliver personalized and interactive therapy [131,132], high user satisfaction [101,131], and the potential for scalability and accessibility [110,147]. Engaging stakeholders and leveraging digital health technologies can promote the adoption of AI-driven CBT in clinical settings [110].

*RQ7: Long-Term Effects and Relapse Prevention*

Digital and AI-driven CBT interventions demonstrate promising long-term effects on sleep quality and relapse prevention. Studies have shown sustained improvements in sleep outcomes at follow-up periods ranging from several weeks to over a year [115,127,132,142,143,146,164]. These interventions also show the potential to reduce the risk of relapse and promote resilience [94,115]. The ability of AI-driven CBT to address underlying cognitive factors and provide ongoing personalized support contributes to its long-term efficacy [101,141].

*RQ8: Patient Perceptions of Usability and Effectiveness*

Patients generally perceive AI-enhanced CBT interventions as highly usable and effective. Studies report high levels of user satisfaction, with many patients recommending these interventions to others [101,131,141]. Patients appreciate the personalized and adaptive nature of AI-driven CBT and the convenience and accessibility of digital platforms [110,115]. Integrating wearable devices and real-time feedback further enhances these interventions’ perceived usability and effectiveness [111,130].

**Figure 11:**
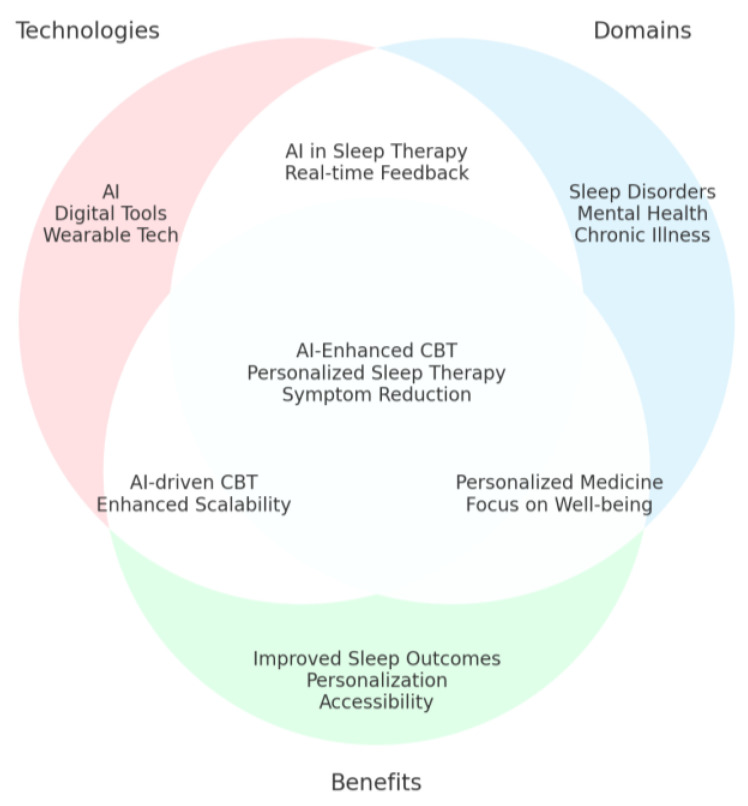
Venn diagram representing the intersection of technologies, domains, and benefits in AI-enhanced personalized CBT for sleep disorders.

The Venn diagram (Figure 11) visually represents the integration of technologies, application domains, and potential benefits of AI-enhanced personalized CBT for sleep disorders. The overlapping sections highlight where these elements intersect, showcasing how AI and wearable tech are applied to sleep disorders and mental health, resulting in improved outcomes such as symptom reduction, personalization, and enhanced scalability. The central overlap reflects the core of AI-driven CBT, combining these aspects to optimize therapy for diverse patient needs and improve long-term treatment effectiveness.

**Figure 12:**
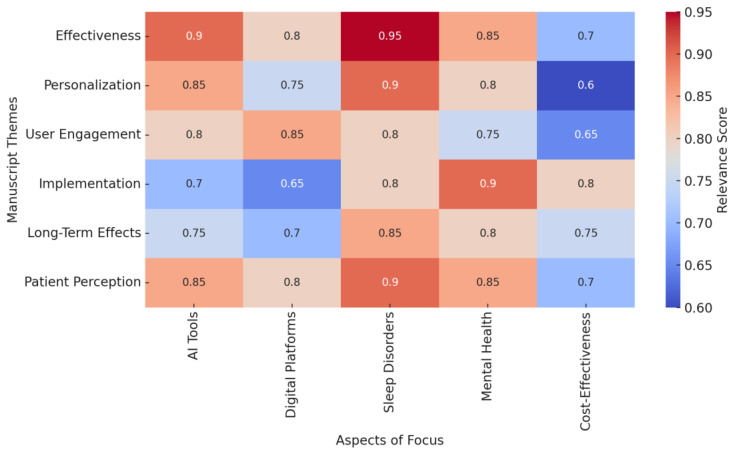
Heatmap depicting the relevance of themes across focus areas on the manuscript’s analysis of AI-driven CBT for sleep disorders.

Moreover, the heatmap below (Figure 12) illustrates the relevance of manuscript themes (effectiveness, personalization, user engagement, implementation, long-term effects, and patient perception) across key focus areas (AI tools, digital platforms, sleep disorders, mental health, and cost-effectiveness). The color gradient, ranging from blue (low relevance) to red (high relevance), emphasizes the distribution of focus and impact within the manuscript’s analysis of AI-driven CBT for sleep disorders.

**Figure 13:**
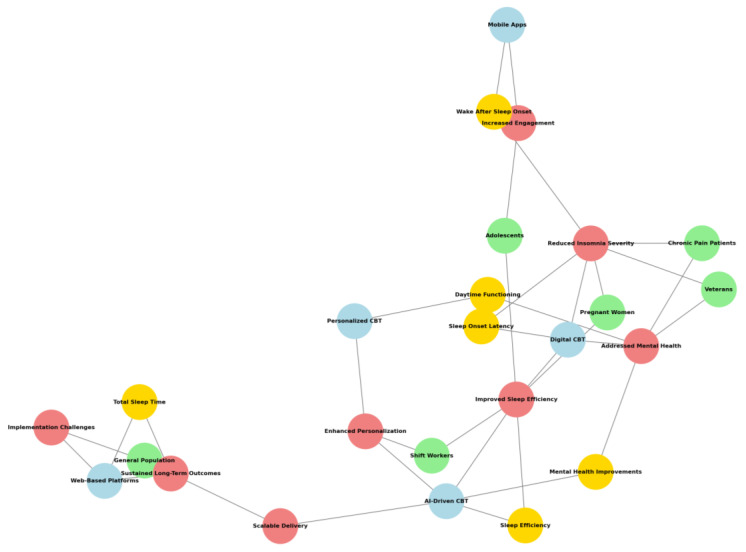
Schema network: CBT for sleep disorders.

The schema network (Figure 13) also provides a detailed view of how participants, findings, intervention types, and outcomes interact within CBT interventions for sleep disorders. Participants, represented in light green, include groups like pregnant women, shift workers, veterans, adolescents, chronic pain patients, and the general population. These groups face unique sleep-related challenges and are key targets for intervention.

Findings, highlighted in light coral, represent the observed benefits and challenges of CBT interventions. Examples include improved sleep efficiency, reduced insomnia severity, enhanced personalization, scalable delivery, sustained long-term outcomes, and addressed mental health concerns. These findings help assess the effectiveness and practicality of various CBT methods. Intervention types in light blue signify the tools and techniques used to deliver CBT. These include AI-driven CBT, personalized CBT, digital CBT, mobile apps, and web-based platforms. These technologies provide tailored, accessible, and scalable solutions to address sleep disorders. Outcomes, depicted in gold, measure the tangible improvements achieved through the interventions. Examples include improved sleep efficiency, reduced sleep-onset latency, decreased wake-after-sleep onset, increased total sleep time, better daytime functioning, and improved mental health.

To summarize, the schema network below (Figure 13) visually illustrates the relationships between participants, findings, intervention types, and outcomes in CBT interventions targeting sleep disorders. Distinct colors are used to differentiate categories:

- Light Green nodes represent participants such as specific groups or demographics.
- Light Coral nodes signify findings that highlight benefits or impacts of the interventions.
- Light Blue nodes indicate intervention types, showcasing methods or tools for CBT delivery.
- Gold nodes reflect measurable outcomes or improvements.

The edges in the network represent relationships between these elements. Participants are linked to findings that reflect their specific benefits, such as pregnant women showing improved sleep efficiency or veterans experiencing reduced insomnia severity and better mental health. Findings connect to intervention types, illustrating how different methods achieve specific results, such as AI-driven CBT enhancing personalization or digital CBT improving sleep-onset latency. Outcomes are tied to both findings and intervention types, demonstrating how observed benefits translate into measurable results, such as web-based platforms improving total sleep time. This network highlights the interconnectedness of CBT for sleep disorders, revealing pathways for optimizing interventions, addressing implementation challenges, and achieving meaningful outcomes for diverse populations.

**Figure 14:**
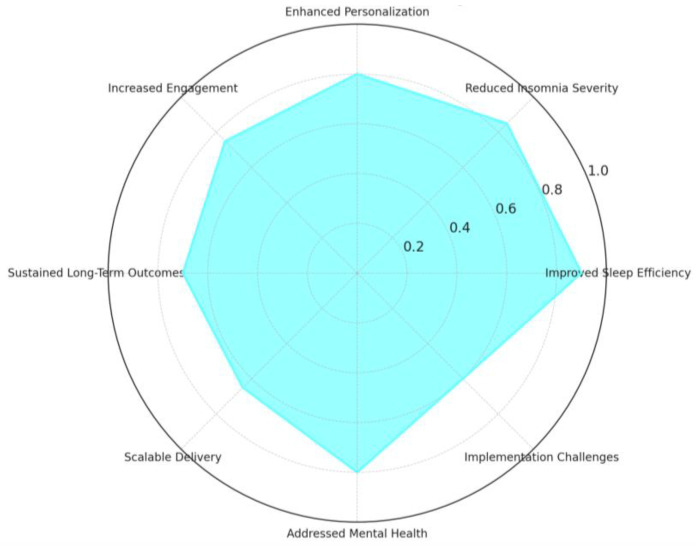
Relevance of main findings in AI-driven CBT for sleep disorders.

Finally, the radar chart (Figure 14) illustrates the relevance of the main findings with a gradient 3D-like color scheme. Key outcomes include improved sleep efficiency, reduced insomnia severity, enhanced personalization, increased engagement, sustained long-term outcomes, scalable delivery, addressed mental health, and implementation challenges. The chart uses a dynamic gradient to improve visual depth and highlight the varying significance of findings.

**Figure 15:**
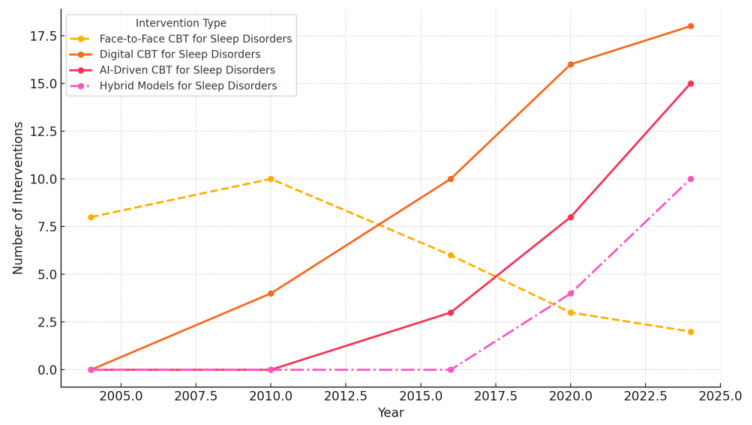
Trend line of CBT interventions for sleep disorders (2004–2024).

A trend line (Figure 15) illustrates the evolution of CBT interventions specifically for sleep disorders from 2004 to 2024. The graph shows the decline of face-to-face CBT, the rise of digital CBT, the emergence and rapid growth of AI-driven CBT, and the increasing adoption of hybrid models that integrate AI-driven insights with therapist support. This visualization highlights the growing focus on technology-driven solutions for sleep disorder management.

### 5.1. Overarching Themes and Future Directions

Several overarching themes emerge from the present study. First, personalization and adaptability are central to the effectiveness of AI-driven CBT interventions. The ability to tailor treatments to individual needs, provide real-time feedback, and adjust interventions based on user progress significantly enhances outcomes. Second, user engagement and adherence remain critical challenges despite AI-driven interventions’ interactive and personalized nature. Strategies to improve retention and address dropout rates are essential for maximizing the impact of these treatments. Third, integrating AI with human support, such as therapist feedback or personalized telephone sessions, can further enhance the effectiveness of digital interventions. This hybrid approach combines the strengths of AI-driven personalization with the benefits of human interaction, potentially leading to better outcomes. Future research should focus on direct comparative studies between AI-driven and standard digital CBT to establish the relative efficacy of these approaches. Additionally, further investigation is warranted into the mechanisms underlying the long-term benefits of AI-driven CBT and strategies to enhance user engagement and address barriers to implementation. Addressing data privacy, security, and technological literacy issues will also be crucial for successfully adopting AI-driven CBT in diverse populations.

### 5.2. Implications for Clinical Practice

Information derived from this systematic review can already be used in clinical practice. AI and machine learning algorithms can help clinicians in the first step: treatment planning. They can help the clinician choose various treatment options and inform the patient about possible additional problems related to their sleep disorder. AI can also provide tools for follow-up, such as initial treatment choice, therapy success, and therapy adjustments over time [165,166,167]. Such new algorithm-driven methods enhance the possibility of providing personalized treatment. This can reduce the trial-and-error gambling familiar with sleep medicine problems, improving patient outcomes. However, distinct training programs are needed to prepare for future AI-based methods among practicing clinicians, especially those in the CBTi field. Several essential considerations need to be made when designing and deploying AI systems for use in therapy settings. Decisions must be made about the presentation of machine-learning outputs and the role of computing systems. Studies show that most people prefer to receive direct input based on predictive performance [168,169,170]. AI can make more accurate individual-level predictions than humans, leading to computers providing better treatment planning advice and risk factor identifications. An important point is maintaining patient privacy and trust; AI tools require patient approval before use in therapy settings. The potential for AI to revolutionize significant aspects of social and behavioral science is considerable. Using deep learning combined with large databases enables discoveries and predictions that were impossible even a decade ago. The increasing shift toward objectifying human psychological problems is already driving the use of AI in therapy settings. However, practice guidelines for AI use in clinical psychology need updating to maximize patient and psychological opportunities while minimizing potential adverse outcomes. The field of AI ethics and regulation is catching up. There is an opportunity to guide the direction of the development and use of AI in treatment settings, and time is of the essence [171,172,173].

### 5.3. Limitations

Although this systematic review offers valuable information on incorporating AI-based personalized CBT-I interventions for sleep disorders, some points must be examined more thoroughly. The variability in study designs, formats of interventions, AI algorithms, and outcomes makes direct comparisons challenging, and more standardized methods in future studies are necessary. While this review incorporates visual representations such as heatmaps and network graphs to synthesize findings, further quantitative meta-analyses can enhance the statistical rigor of comparisons between AI-supported and traditional CBT interventions. This review does not include formal statistical assessments for publication bias (e.g., funnel plots or Egger’s test), which is a limitation. Moreover, the efficacy and durability of AI-enhanced CBT-I interventions in the long term also require investigation through longitudinal studies to determine their impact over lengthy periods. Regular AI-based mental health care innovations present promising avenues, and research over the next few years must center on the ongoing growth of such technology, solutions for ethical challenges, and making AI technology easier to implement in clinical care for maximum access, utilization, and personalized outcome.

## 6. Conclusions

In conclusion, AI-enhanced CBT-I interventions represent a significant advancement in treating sleep disorders. These interventions offer personalized, scalable, and accessible solutions that can lead to substantial and sustained improvements in sleep quality. While challenges remain regarding user engagement, technological barriers, and implementation logistics, the evidence strongly supports the continued development and adoption of AI-driven CBT in clinical practice. By leveraging the strengths of AI and addressing the identified challenges, we can transform the landscape of insomnia treatment, providing practical and personalized care to a broader population and improving the lives of millions affected by sleep disorders.

## Figures and Tables

**Table 1:** Research articles of systematic analysis (n = 78).

Authors	Study Objectives	Participants (n)	Methodology	Main Findings
Alessi, C. et al. (2020) [87]	An integrated approach of cognitive behavioral therapy for insomnia and a PAP adherence program improved sleep and PAP use in adults with comorbid insomnia and obstructive sleep apnea.	125	The intervention consisted of a structured, manual-based treatment that integrated cognitive behavioral therapy for insomnia (CBTI) with a positive airway pressure (PAP) adherence program. The intervention was delivered in 5 weekly 1 h individual sessions by a “sleep coach” who had a master’s degree level of education but no clinical training or licensure. The CBTI components included stimulus control, sleep restriction, sleep hygiene, relaxation techniques, and cognitive therapy techniques. The PAP adherence components included education about OSA and PAP, reviewing the participant’s individual benefits and challenges with PAP use and providing individualized recommendations and strategies to address challenges. The sleep coach received weekly 1 h telephone supervision from a behavioral sleep medicine psychologist to review participant progress and problem-solve issues with CBTI and PAP adherence.	- PSQI (higher score indicates worse sleep quality): 3.2-point greater improvement in intervention group compared to control (*p* < 0.001). - Sleep-onset latency (SOL-D): 16.2 min greater reduction in intervention group compared to control (*p* = 0.013). - Wake-after-sleep onset (WASO-D): 20.5 min greater reduction in intervention group compared to control (*p* = 0.019). - Sleep efficiency (SE-D): 10.5% greater improvement in intervention group compared to control (*p* = 0.001). - Sleep efficiency (SE-A): 4.4% greater improvement in intervention group compared to control (*p* < 0.001). - Improvement in PAP use at 3 months: - Hours of PAP use per night: 1.3 more hours per night in intervention group compared to control (*p* < 0.001). - Nights with PAP use ≥4 h: 17.4 more nights in intervention group compared to control (*p* < 0.001). - The improvements in sleep and PAP use were generally maintained at the 6-month follow-up as well.
Alessi, C. et al. (2016) [88]	This paper tests a cognitive behavioral therapy for insomnia program designed for use by nonclinicians.	159	The intervention was a cognitive behavioral therapy for insomnia (CBT-I) program delivered by non-clinician “sleep coaches” with weekly telephone supervision by a psychologist with expertise in behavioral sleep medicine. The intervention was delivered either in a small group format (3–5 participants) or individually, with 5 one-hour sessions over 6 weeks.	- At post-treatment, the intervention group showed significantly greater improvements than the control group in - Sleep-onset latency (SOL-D): 23.4 min greater decrease. - Wake-after-sleep onset (WASO-D): 17.7 min greater decrease. - Total wake time (TWT-D): 68.4 min greater decrease. - Sleep efficiency (SE-D): 10.5% greater increase. - At 6 months, the intervention group showed significantly greater improvements than the control group in - SOL-D, TWT-D, and SE-D. - At 12 months, the treatment effects remained significant for SOL-D, TWT-D, and SE-D. - For the Pittsburgh Sleep Quality Index (PSQI), the intervention group showed significantly greater improvements than the control group at post-treatment (3.4 points better), 6 months (2.4 points better), and 12 months (2.1 points better). - For the Insomnia Severity Index (ISI), the intervention group showed significantly greater improvements than the control group at each follow-up time point (effect sizes ranging from 0.54 to 0.85).
Anderson, K. et al. (2014) [89]	Online cognitive behavioral therapy for insomnia can be personalized and effective, but screening for other sleep disorders is necessary.	75	The intervention was an online cognitive behavioral therapy (CBT) for insomnia disorder, delivered through a modern interactive video-based website. Participants underwent a rigorous screening process to exclude those with other sleep disorders or mental health conditions before starting the therapy, which was personalized based on their responses.	- Significant increase in sleep efficiency. - Significant increase in sleep latency. - Modest, non-significant improvements in total sleep time. - Majority of users reported improved sleep quality.
Arnal, P. et al. (2020) [90]	A personalized digital CBT-I program using hardware, software, and therapist support shows high engagement and effectiveness for treating insomnia.	1304	The intervention was a 6-week cognitive behavioral therapy for insomnia (CBT-i) program delivered through the Dreem platform. Participants had to complete at least one week of the program to be included in the analysis.	- Clinically significant decrease of 7.42 points on the Insomnia Severity Index (ISI) (*p* < 0.001). - 35% reduction in objective wake-after-sleep onset (obj-WASO) (*p* < 0.001). - 37% reduction in objective number of awakenings (obj-Awakenings) (*p* < 0.001). - 2.56-point increase in objective sleep efficiency (obj-SE) (*p* < 0.001). - 22% reduction in objective sleep-onset latency (obj-SOL) (*p* < 0.001). - 41% reduction in subjective sleep-onset latency (subj-SOL) (*p* < 0.001). - 8.9-point increase in subjective sleep efficiency (subj-SE) (*p* < 0.001). - 16% increase in subjective sleep duration (subj-SD) (*p* < 0.001).
Bei, B. et al. (2021) [91]	A scalable cognitive behavioral therapy intervention improved sleep during pregnancy and up to 2 years postpartum, especially for those with elevated insomnia symptoms.	163	The intervention was a scalable cognitive behavioral therapy (CBT) sleep intervention. The intervention consisted of a 1 h telephone session and automated multimedia emails, delivered from the third trimester of pregnancy until 6 months postpartum.	- Lower insomnia severity and sleep disturbance at the pregnancy endpoint (*p* ≤ 0.001). - Lower sleep-related impairment at the pregnancy endpoint (*p* ≤ 0.001) and at 24 months postpartum (*p* = 0.012–0.052). - No significant group differences across the first postpartum year. - Participants with elevated insomnia symptoms at baseline had significantly lower insomnia symptoms throughout the first postpartum year with the CBT intervention compared to control.
Blom, K. et al. (2015) [92]	Internet-delivered and group-delivered cognitive behavioral therapy for insomnia were found to be equally effective.	48	Guided Internet-delivered CBT (ICBT) and group-delivered CBT (GCBT) for insomnia.	- Insomnia Severity Index (ISI): - ICBT: Large effect sizes (Cohen’s *d* = 1.8 at post-treatment and 2.1 at 6-month follow-up). - GCBT: Large effect sizes (Cohen’s *d* = 2.1 at post-treatment and 2.2 at 6-month follow-up). - Response rate (ISI reduction > 7 points): - ICBT: 66% responders. - GCBT: 66% responders. - Remission rate (based on diagnostic criteria): - ICBT: 63% in remission. - GCBT: 75% in remission. - Sleep diary data: Moderate to large effect sizes for both groups.
Bostock, S. et al. (2016) [93]	Digital cognitive behavioral therapy for insomnia improves sleep and work productivity in adults.	270	The intervention was a digital cognitive behavioral therapy (dCBT) for insomnia, consisting of 6 online sessions delivered by an animated therapist. A total of 135 participants received this dCBT intervention.	- Sleep quality (SCI scores): - dCBT: Large effect size (Cohen’s *d* = 1.10), *p* < 0.0001. - WL: Small effect size (d = 0.34). - Work productivity (“presenteeism”): - dCBT: Medium effect size (*d* = 0.64), *p* = 0.001. - WL: Negligible effect size (*d* = 0.09). - Absenteeism: - No statistically significant effects.
Brooks, A. et al. (2018) [94]	This study protocol examines the feasibility and efficacy of an online cognitive behavioral therapy for insomnia among individuals with alcohol use disorder.	70	The intervention is the SHUTi (Sleep Healthy Using The Internet) program, which is an Internet-based cognitive behavioral therapy for insomnia (CBT-I) intervention. The intervention will be delivered in two phases—a feasibility phase with 10 participants and then a larger RCT with 30 participants per group.	Not mentioned (the abstract does not provide any quantitative results or intervention effects from the study).
Carney, C. et al. (2017) [95]	This paper compares cognitive behavioral therapy for insomnia (CBT-I) plus antidepressant medication against treatments targeting solely depression or insomnia, finding that CBT-I groups improved on objective sleep measures.	107	(1) Cognitive behavioral therapy for insomnia (CBT-I): 4 weekly individual sessions followed by 2 sessions at weeks 6 and 8. (2) Antidepressant medication (escitalopram): 10 mg daily. (3) Sleep Hygiene (SH): 4 weekly individual sessions.	- Objective sleep (PSG): - CBT-I + placebo drug (PD) group improved on total wake time (TWT), while the antidepressant (AD) + sleep hygiene (SH) group worsened (*p* = 0.02). - No significant group differences in sleep efficiency (SE). - Between-group effect sizes for PSG TWT: small for CBT-I + AD vs. other groups, medium for CBT-I + PD vs. AD + SH. - Subjective sleep (sleep diaries): - All groups improved on Insomnia Severity Index (ISI) and sleep diary measures, with no significant group differences. - Between-group effect sizes for ISI: medium for CBT-I + AD vs. CBT-I + PD, small for other comparisons. - Depression (HAMD17): - All groups improved significantly, with no significant group differences. - Between-group effect sizes for HAMD17: small for all comparisons.
Castro, L. et al. (2021) [96]	A fully automated digital cognitive behavioral therapy for insomnia using chatbot and AI shows feasibility in improving sleep parameters and engagement.	3139	(1) A digital coach that interacts with users daily for 5–10 min. (2) Users complete tailored diaries. (3) The digital coach delivers CBT-I knowledge “pills” in around 50 sessions over around 7 weeks. (4) The Insomnia Severity Index (ISI) is used to assess insomnia severity before and after the sleep restriction cycles, and the program uses an algorithm to revise the ISI weekly.	- 16.8 min from first to second week. - 67.3 min after week seven. - Increase in sleep efficiency: - 34% among women. - 26% among men. - Therapeutic response (ISI reduction ≥ 8): - 66% of participants completed all sessions. - 34% crossed half-way. - Insomnia remission (ISI ≤ 7): - 55% of those with subthreshold insomnia at baseline. - 33% of those with clinical insomnia at baseline.
Chan, C. et al. (2021) [97]	A smartphone-delivered self-help cognitive behavioral therapy for insomnia is effective in alleviating major depression and insomnia.	320	The intervention was a 6-week smartphone-delivered self-help cognitive behavioral therapy for insomnia (CBT-I) program called proACT-S.	- Depression severity (CES-D): Cohen’s d = 0.86, 95% CI (−10.11 to −5.37), significant improvement in treatment group vs. control. - Insomnia severity (ISI): Cohen’s d = 1.00, 95% CI (−5.93 to −3.53), significant improvement in treatment group vs. control. - Anxiety severity (HADS-A): Cohen’s d = 0.83, 95% CI (−3.75 to −1.96), significant improvement in treatment group vs. control. - Sleep quality (PSQI): Cohen’s d = 0.91, 95% CI (−3.34 to −1.83), significant improvement in treatment group vs. control.
Cheng, P. et al. (2020) [98]	Prior digital cognitive behavioral therapy for insomnia increased health resilience during the COVID-19 pandemic.	208	The intervention was 6 sessions of self-guided digital cognitive behavioral therapy for insomnia (dCBT-I), delivered via an animated “virtual therapist” who guided the participant’s progress.	- Approximately 3 points lower insomnia severity index (ISI) scores during the COVID-19 pandemic (*p* = 0.001). - 51% lower odds of resurgent moderate to severe insomnia during COVID-19 among those who previously reported insomnia symptom resolution (*p* < 0.001). - Trend towards lower general stress levels during COVID-19 (*p* = 0.055). - Lower COVID-19 specific stress as measured by the Impact of Events Scale (*p* = 0.03). - 1.3 points lower depressive symptoms during COVID-19 (*p* = 0.01). - 57% lower odds of moderate to severe depressive symptoms during COVID-19 (*p* < 0.001). - 58% lower odds of low global physical health (*p* = 0.002). - 42% lower odds of low global mental health (*p* < 0.001).
Cheng, P. et al. (2019) [99]	Digital cognitive behavioral therapy for insomnia can prevent depression.	1385	The intervention was digital cognitive behavioral therapy for insomnia (dCBT-I) delivered via the Sleepio program. Participants received access to the program for 12 weeks and could complete the 6 core sessions on a weekly basis. The intervention covered behavioral, cognitive, and relaxation components, as well as sleep hygiene, and was delivered by an animated “virtual therapist”.	- The dCBT-I group had a significantly greater reduction in depression severity at 1-year follow-up compared to the control group, with a 4.0-point decrease on the QIDS-SR 16 scale versus a 1.7-point decrease in the control group (*p* < 0.001). - The dCBT-I group had a significantly lower rate of moderate-to-severe depression at 1-year follow-up (20.9%) compared to the control group (35.5%). - The dCBT-I group had a significantly higher rate of depression remission at 1-year follow-up, with a 51% higher remission rate compared to the control group (*p* < 0.001). - For participants with minimal to no depression at baseline, the dCBT-I group had approximately half the incidence of moderate-to-severe depression at 1-year follow-up compared to the control group (9.6% vs. 18.8%, *p* < 0.01).
Clarke, G. et al. (2015) [100]	Cognitive behavioral treatment of insomnia and depression in adolescents shows promise but does not involve artificial intelligence.	41	Sleep hygiene (SH) control condition combined with cognitive behavioral therapy for depression (CBT-D), delivered in 10 weekly sessions. Cognitive behavioral therapy for insomnia (CBT-I) combined with cognitive behavioral therapy for depression (CBT-D), delivered in 10 weekly sessions.	- CBT-I arm: Improved by 99 min from baseline to week 12. - SH arm: No improvement. - Insomnia Severity Index “caseness”: - Medium–large effects favoring the CBT-I arm. - Depression outcomes: - Higher percentage recovered. - Faster time to recovery.
Cliffe, B. et al. (2020) [101]	Digital cognitive behavioral therapy for insomnia is feasible and effective for improving sleep and mental health in adolescents with mental health problems.	49	The intervention was a 6-session, 20-min per session digital cognitive behavioral therapy for insomnia (digital CBTi) program called Sleepio, with additional weekly 15 min support telephone calls.	- Improvement in sleep efficiency (*p* = 0.005). - Improvement in sleep quality (*p* = 0.001). - Improvement in sleep as measured by the Sleep Condition Indicator (*p* = 0.001). - Improvement in insomnia severity as measured by the Insomnia Severity Index (*p* = 0.001). - Reduction in symptoms of low mood as measured by the Mood and Feelings Questionnaire (*p* = 0.03). - Reduction in symptoms of anxiety as measured by the Revised Child Anxiety and Depression Scale (*p* = 0.005). - The number of participants scoring in the clinical range for insomnia was reduced from 19 to 10 on the SCI and from 19 to 6 on the ISI.
Darden, M. et al. (2020) [102]	Digital CBT is the most cost-effective insomnia treatment compared to other options in the US.	100,000	Digital CBT (Sleepio): A fully automated digital CBT intervention comprising the full range of evidence-based cognitive and behavioral techniques, highly personalized to individual users. Pharmacotherapy: A 100-day course of generic zolpidem at a cost of USD 144.10. Individual CBT: 6 sessions of individual CBT at a cost of USD 1044. Group CBT: 6 sessions of group CBT at a cost of USD 172.50 per individual.	- Digital CBT (Sleepio): 60% (range 55–62%) remission rate at 6 months. - Pharmacotherapy: 47.7% remission rate at 6 months. - Individual CBT: 60% remission rate at 6 months. - Group CBT: 60% remission rate at 6 months. - No treatment: 15% remission rate at 6 months.
Edinger, J. et al. (2022) [103]	Digital and therapist-delivered cognitive behavioral therapies for insomnia are both effective for treating insomnia in sleep apnea patients.	305	Digital cognitive behavioral therapy for insomnia (dCBTI). Therapist-delivered cognitive behavioral therapy for insomnia (TCBTI).Both interventions were provided for 8 weeks, with some participants receiving an additional 8 weeks of one of the interventions if they did not achieve remission after the initial 8 weeks.	- Reduction in Insomnia Severity Index (ISI) scores: - dCBTI and TCBTI groups showed significantly greater (*p* = 0.0001) and comparable reductions in ISI scores compared to the control group, moving participants from moderate to mild insomnia symptoms. - Responder rates (>8-point decline in ISI): - dCBTI: 30.5%. - TCBTI: 50%. - Control: 19.2%. - The responder rate was significantly higher (*p* < 0.0001) for the TCBTI group compared to dCBTI and control. - Remission rates (ISI < 8): - dCBTI: 30.5%. - TCBTI: 29.2%. - Control: 19.2%. - The remission rates were significantly higher (*p* = 0.001) for both dCBTI and TCBTI compared to control but comparable between the two active interventions.
Ellis, J. et al. (2015) [104]	A single session of cognitive behavioral therapy for insomnia is effective for treating acute insomnia.	40	The intervention consisted of a single 60 to 70 min session of cognitive behavioral therapy for insomnia (CBT-I), along with a self-help pamphlet that the participants were given.	- Significantly lower Insomnia Severity Index (ISI) scores in the CBT-I group compared to controls at follow-up (*p* < 0.05). - 60% of the CBT-I group had remitted (ISI < 10) at follow-up compared to 15% of controls (*p* < 0.003). - 50% of the CBT-I group had remitted (ISI < 8) at follow-up compared to 10% of controls (*p* < 0.01). - Significant improvements in sleep latency (SL), wake-after-sleep onset (WASO), and sleep efficiency (SE) in the CBT-I group compared to controls (all *p* < 0.05).
Enomoto, K. et al. (2022) [105]	Cognitive behavioral therapy for insomnia is the most effective treatment option for individuals with comorbid insomnia and chronic pain.	1094	Cognitive behavioral therapy for insomnia (CBT-I). Cognitive behavioral therapy for pain (CBT-P). Cognitive behavioral therapy for insomnia and pain (CBT-IP).The abstract does not provide any details on the frequency, duration, or amount/dose of these interventions.	- Sleep at post-treatment: SMD = −0.99, 95% CrI = −1.50 to −0.54 (significant). - Pain at post-treatment: significant. - Disability at post-treatment: significant. - Depression at post-treatment: significant. - Sleep at follow-up: significant. - CBT-IP vs. control: - Sleep at post-treatment: SMD = −0.70, 95% CrI = −1.60 to −0.08 (significant). - CBT-P vs. control: - No significant differences for any outcomes.
Espie, C. et al. (2019) [106]	Digital cognitive behavioral therapy for insomnia improves functional health, psychological well-being, and sleep-related quality of life.	1711	The intervention was digital cognitive behavioral therapy (dCBT) for insomnia, delivered using web and/or mobile channels, in addition to usual treatment. The control group received sleep hygiene education (SHE) through a website and a downloadable booklet in addition to usual treatment.	- Functional health: - Week 4: 0.90-point improvement (95% CI: 0.40–1.40, *p* < 0.01). - Week 8: 1.76-point improvement (95% CI: 1.24–2.28, *p* < 0.01). - Week 24: 1.76-point improvement (95% CI: 1.22–2.30, *p* < 0.01). - Psychological well-being: - Week 4: 1.04-point improvement (95% CI: 0.28–1.80, *p* < 0.01). - Week 8: 2.68-point improvement (95% CI: 1.89–3.47, *p* < 0.01). - Week 24: 2.95-point improvement (95% CI: 2.13–3.76, *p* < 0.01). - Sleep-related quality of life: - Week 4: 8.76-point improvement (95% CI: −11.83 to −5.69, *p* < 0.01). - Week 8: 17.60-point improvement (95% CI: −20.81 to −14.39, *p* < 0.01). - Week 24: 18.72-point improvement (95% CI: −22.04 to −15.41, *p* < 0.01). These improvements were mediated by a large improvement in insomnia symptoms.
Espie, C. et al. (2014) [107]	Online cognitive behavioral therapy modifies sleep-related attributions, night-time thought content, and psychopathology, which partly mediates improvement in insomnia.	164	Cognitive behavioral therapy (CBT)—6 online sessions delivered by an animated therapist, with automated web/email support, and access to a moderated online community. Imagery Relief Therapy (IRT)—6 online sessions delivered by an animated therapist, with automated web/email support (a placebo intervention). Treatment as usual (TAU).	- CBT vs. IRT (placebo): - SDQ “trying too hard” domain: d = 0.76 (significant). - GCTI “rehearsal and planning” domain: d = 0.62 (significant). - GCTI “sleep and sleeplessness” domain: d = 0.74 (significant). - CBT vs. TAU (control): - Larger effects than IRT vs. TAU (which were small to moderate).
Eyal, S. et al. (2020) [108]	A mobile app providing personalized digital cognitive and behavioral therapy was effective in improving insomnia symptoms in students, but engagement remains a challenge.	892	The intervention was a mobile app called “Refresh by Sleeprate” that provided a sleep assessment followed by weekly cycles of personalized digital cognitive and behavioral reframing. The duration of the intervention was at least one week, with an average of 5.6 weekly nights and 28 total nights for those who completed the intervention.	- Reduced sleep latency from 28.8 min to 22.1 min (*p* < 0.001), with a larger reduction from 53.9 min to 32.7 min for those with initial sleep latency >30 min (*p* < 0.001). - Reduced wake-after-sleep onset from 46.3 min to 35.8 min for those with initial WASO >30 min (*p* < 0.05). - Increased sleep efficiency by 1.6% overall (*p* < 0.002) and by 7.1% for those with initial sleep efficiency <85% (*p* < 0.001).
Geagea, L. et al. (2022) [109]	This pilot study found that cognitive behavioral therapy for insomnia is effective in treating insomnia and comorbid conditions in individuals with cannabis use disorder.	19	The intervention was cognitive behavioral therapy for insomnia (CBTi), consisting of 4 sessions, with participants wearing an actigraphy device for 1 week before and 1 week after the 4 CBTi sessions.	- Insomnia Severity Index (ISI) score decreased from moderately severe insomnia at baseline to no clinically significant insomnia after CBTi, with sustained decrease at 3- and 6-month follow-up. - Significant decrease in sleep-onset latency (SOL) measured by actigraphy after CBTi. - 80% of participants reported a decrease in cannabis use 3 months after CBTi. - Significant and sustained decrease in mean Patient Health Questionnaire-4 (PHQ-4) scores after CBTi. - Levels of 3 out of 4 biomarkers (IL-2, IL-6, and CRP) decreased, though only trending towards significance, 6 months after CBTi.
Germain, A. et al. (2024) [110]	A digital clinical decision support platform can augment CBTI capabilities and yield rapid, clinically meaningful improvements in sleep among active-duty service members with insomnia.	245	The intervention was a digital clinical decision support (CDS) platform called COAST (NOCTEM^®®^ Health, Inc., Pittsburgh, PA, USA) that was used by mental healthcare providers (MHCPs) to deliver cognitive behavioral therapy for insomnia (CBTI) to active-duty service members (ADSMs) with insomnia. The platform consisted of a clinician portal to remotely monitor and manage patients and a patient app to collect sleep diaries and display CBTI recommendations. The average treatment duration was 5 ± 1 weeks.	- Treatment response rate: 83.5% of active-duty service members (ADSMs) met the criteria for treatment response. - Remission rate: 70.3% of ADSMs met the criteria for insomnia remission. - Clinically meaningful improvements in the following sleep outcomes from baseline to the end of the intervention: - Sleep latency (SL). - Wake-after-sleep onset (WASO). - Sleep efficiency (%SE). - All of these sleep parameter improvements had effect sizes (Cohen’s *d*) greater than 0.5, indicating medium-to-large effects.
Grierson, A. et al. (2020) [111]	Unguided Internet-delivered cognitive behavioral therapy for insomnia is effective for individuals with potential psychiatric comorbidities.	317	The intervention was an unguided, self-guided online cognitive behavioral therapy program for insomnia (iCBT-I) consisting of 4 online lessons with automated web support but no human guidance or support.	- Insomnia symptoms: Large reduction (effect size *g* = 1.11), statistically significant. - Psychological distress: Moderate reduction (effect size *g* = 0.55), statistically significant. - General wellbeing: Small improvement (effect size *g* = 0.37), statistically significant. - Insomnia remission rate: 65% of participants with clinically significant insomnia at baseline remitted after the intervention.
Haynes, J. et al. (2018) [112]	Cognitive behavioral therapy is an effective treatment for insomnia, but the paper does not mention personalization using artificial intelligence.		The intervention is cognitive behavioral therapy for insomnia (CBTi), which includes the following components: sleep hygiene, stimulus control, sleep restriction, cognitive therapy, and relaxation training.	- Remission of insomnia. - Reduced sleep-onset latency. - Reduced wakefulness after sleep onset. - Improved sleep efficiency. - Improved sleep quality.
Heenan, A. et al. (2019) [113]	A group-based cognitive behavioral therapy for insomnia tailored to patients with cardiovascular disease improved their sleep and reduced anxiety and depression.	47	The intervention was a 6-week group-based cognitive behavioral therapy for insomnia (CBT-I) program that was tailored for patients with cardiovascular disease (CVD).	- Sleep duration, maintenance, efficiency, latency, and quality: Significantly improved (*p* < 0.05). - Symptoms of anxiety: Significantly reduced (*p* < 0.05). - Symptoms of depression: Significantly reduced (*p* < 0.05). - Symptoms of insomnia: Significantly reduced (*p* < 0.05).
Henry, A. et al. (2020) [114]	Cognitive behavioral therapy for insomnia can improve both insomnia and depressive symptoms, with insomnia improvement mediating effects on depression.	3352	The intervention was a fully-automated digital cognitive behavioral therapy (CBT) intervention for insomnia called Sleepio.	- Digital CBT for insomnia significantly improved insomnia symptoms at post-intervention (*g* = 0.76, *p* < 0.001) and follow-up (*g* = 0.69, *p* < 0.001) compared to control. - Digital CBT for insomnia significantly reduced depressive symptoms at post-intervention (*g* = 0.48, *p* < 0.001) and follow-up (*g* = 0.42, *p* < 0.001) compared to control. - Participants receiving digital CBT were 2.9 times more likely to experience clinically significant improvement in depressive symptoms (PHQ-9 < 10) at post-intervention (OR = 2.9, 95% CI = 2.34, 3.65, *p* < 0.001) and 2.4 times more likely at follow-up (OR = 2.4, 95% CI = 1.96, 3.14, *p* < 0.001) compared to control. - Participants receiving digital CBT were 2.9 times more likely to achieve a reliable change in depressive symptoms (PHQ-9 reduction ≥ 6) at post-intervention (OR = 2.93, 95% CI = 2.4, 3.7, *p* < 0.001) and 2.7 times more likely at follow-up (OR = 2.7, 95% CI = 2.12, 3.37, *p* < 0.001) compared to control. - Improvements in insomnia symptoms at mid-intervention (weeks 3–4) mediated 87% of the intervention effect on depressive symptoms at post-intervention (weeks 8–10).
Horsch., C. et al. (2017) [115]	A fully automated mobile phone app delivering cognitive behavioral therapy for insomnia was found to be effective.	151	- A sleep diary. - A relaxation exercise (1–16 min in duration, chosen by the participant). - A sleep restriction exercise (introduced after 6 sleep diaries and average sleep efficiency < 85%, with an algorithm to calculate ideal and maximum time in bed). - Sleep hygiene and educationThe basic program duration was 6-7 weeks, depending on the participant’s adherence.	- Insomnia severity: *d* = −0.66 (significant). - Sleep efficiency: *d* = 0.71 (significant). - Wake-after-sleep onset: significant improvement. - Number of awakenings: significant improvement. - Pittsburgh Sleep Quality Index (PSQI): significant improvement. - Center for Epidemiological Studies Depression (CES-D) scale: significant improvement. - Hospital Anxiety and Depression Scale (HADS): significant improvement. - Sleep-onset latency, time in bed, terminal wakefulness, total sleep time, and Dysfunctional Beliefs and Attitudes about Sleep (DBAS-16) scale showed no significant effects. - At 3-month follow-up, improvements remained significant except for number of awakenings.
Hoyt, T. et al. (2022) [116]	Cognitive behavioral therapy for insomnia is effective in treating military personnel with comorbid insomnia and obstructive sleep apnea.	73	The intervention was individual cognitive behavioral therapy for insomnia (CBT-I) provided to all 73 participants at a specialty sleep clinic.	- Significant improvement in sleep-onset latency. - Significant improvement in wake-after-sleep onset. - Significant improvement in sleep efficiency. - Significant reduction in number of awakenings. - Significant improvement in Insomnia Severity Index symptoms. - 26% of patients showed clinically significant improvement in insomnia symptoms.
Hussaini, F. et al. (2024) [117]	A self-guided insomnia management program using a CBT-I mobile app can improve sleep quality and insomnia severity in primary care.	23	The intervention was a self-guided insomnia management program delivered through the CBT-I Coach mobile app, along with sleep hygiene education. Family medicine providers also received education on insomnia and recommended the use of CBT-I as the primary management approach. Participants were recruited over an 8-week period, including both referrals and patients identified through chart reviews.	- Significant improvement in Insomnia Severity Index (ISI) scores, from a baseline mean of 19.26 (moderate insomnia) to a final mean of 14.04 (sub-threshold insomnia), which was statistically significant (*p* < 0.001) with a large effect size (Cohen’s *d* = 0.93). - 54.5% of participants assessed their insomnia as “improved”, 36.4% as “stable”, and only 9.1% as “worse” in response to the intervention. - 71.4% of those taking prescribed medications rated their insomnia as “stable”, 66.7% of those taking melatonin rated their insomnia as “improved”, and 66.7% of those taking nothing at all rated their insomnia as “improved”. - There was neither an increase in dosage nor a new prescription for any sleep aids or medications during the study.
Ito-Masui, A. et al. (2023) [118]	A 4-week, physician-assisted, Internet-delivered CBT program incorporating machine learning–based well-being prediction increased sleep duration and subjective sleep quality in shift workers at high risk of sleep disorders.	61	Well-being prediction using a machine learning model based on data from a fitness tracker and daily surveys. Personalized sleep advice from physicians 3–4 times per week based on the fitness tracker data and surveys. Visualization of activity and sleep data from the fitness tracker.	- Increase in mean daily sleep duration of 0.52 h (31 min) from baseline to week 4 (*p* = 0.02). - Significant improvement in subjective sleep quality as measured by the Pittsburgh Sleep Quality Index, decreasing from 9.10 at baseline to 7.84 after the intervention (*p* = 0.001). - No significant improvements in the 5 subjective well-being scores, except for an improvement in morning energy levels (*p* = 0.01). - Significant improvement in GHQ scores indicating reduced psychological distress (*p* = 0.046). - Significant increase in burnout scores for emotional exhaustion (*p* = 0.04) and depersonalization (*p* = 0.001).
Jernelöv, S. et al. (2019) [119]	A CBT-i-based group treatment improved insomnia severity in adults with ADHD but does not involve personalized AI-based therapy.	19	Group-delivered CBT-i-based treatment, consisting of 10 weekly 90 min group sessions and scheduled telephone support, provided as an adjunct to usual care at the clinic for adult ADHD patients with self-reported sleep problems.	- Insomnia severity (Insomnia Severity Index) improved by 4.5 points (95% CI 2.06–6.99, *p* = 0.002) immediately after the behavioral treatment. - Insomnia severity improved by 6.8 points (95% CI 4.71–8.91, *p* < 0.0001) at 3-month follow-up.
Kallestad, H. et al. (2021) [120]	This paper compares the efficacy of digital vs. face-to-face cognitive behavioral therapy for insomnia but does not involve personalization or artificial intelligence.	101	Individual face-to-face CBT-I delivered over 6–9 weeks with 3–8 sessions, consisting of psychoeducation, sleep restriction, stimulus control, and cognitive therapy. Digital CBT-I delivered via the SHUTi program over 6 months, consisting of the same components as the face-to-face CBT-I.	- Face-to-face CBT-I was superior to digital CBT-I in reducing insomnia severity at 33 weeks, with a mean difference of 2.8 points on the Insomnia Severity Index (95% CI −4.8 to −0.8, *p* = 0.007, Cohen’s *d* = 0.7). - Face-to-face CBT-I was superior to digital CBT-I in reducing insomnia severity at 9 weeks, with a mean difference of 4.6 points on the Insomnia Severity Index (95% CI −6.6 to −2.7, *p* < 0.001, Cohen’s *d* = 1.2). - The response rate at 9 weeks was significantly higher for face-to-face CBT-I (70%) compared to digital CBT-I (43%), a difference of 27% (95% CI: 7% to 44%, *p* = 0.009). - The remission rate at 33 weeks was significantly higher for face-to-face CBT-I (56%) compared to digital CBT-I (24%), a difference of 32% (95% CI: 12% to 49%, *p* = 0.002).
Kalmbach, D. et al. (2022) [121]	Cognitive behavioral therapy for insomnia can alleviate and prevent suicidal ideation by improving sleep.	658	The intervention was digital cognitive behavioral therapy for insomnia (digital CBTI).	- For participants with baseline suicidal ideation (SI): - CBTI resulted in significantly lower rates of SI at post-treatment (30.0% vs. 54.5%, *p* = 0.005) and 1-year follow-up (29.6% vs. 46.8%, *p* = 0.042) compared to control. - For participants without baseline SI: - CBTI did not directly prevent new onset SI. - However, participants who achieved insomnia remission had significantly lower rates of new onset SI at post-treatment compared to non-remitters (1.5% vs. 6.5%, *p* = 0.009). - About half of the beneficial effects of CBTI on reducing suicidal ideation were mediated through the remission of insomnia symptoms.
Kalmbach, D. et al. (2020) [122]	Digital cognitive behavioral therapy for insomnia improves sleep quality and duration in pregnant women.	91	Digital cognitive behavioral therapy for insomnia (digital CBTI).	- Reductions in Insomnia Severity Index (ISI) scores by 4.91 points (*p* < 0.001) and Pittsburgh Sleep Quality Index (PSQI) scores by 2.98 points (*p* < 0.001) from pre- to post-treatment. - Increases in nightly sleep duration by 32 min (*p* = 0.008) from pre- to post-treatment. - Longer sleep duration by 40 min per night (*p* = 0.01) and better sleep maintenance after childbirth, compared to the control group. - No significant effects on depression or cognitive arousal.
Kim, J. et al. (2024) [123]	An IoT device that delivers personalized CBT-I prompts can significantly reduce insomnia severity and increase sleep time.	65	The intervention was the “Full Sleep” Internet of Things (IoT) device that included an intelligence-sensing radar to passively track sleep patterns and deliver in-the-moment behavioral prompting to promote adherence to cognitive behavioral therapy for insomnia (CBT-I) directives. The device was used by participants as part of a 6–8-week program.	- Insomnia Severity Index (ISI) decreased from 19.3 (SD = 2.5) at baseline to 7.4 (SD = 4.6) post-treatment, a 62% decrease (*p* < 0.001). - Total sleep time increased from 6.3 h (SD = 1.4) to 6.8 h (SD = 1.4), a statistically significant increase (*p* < 0.05). - Sleep efficiency increased from 73.6% (SD = 13.7%) to 88.6% (SD = 8.9%), a statistically significant increase (*p* < 0.05). - Participants had statistically significant reductions in dysfunctional beliefs about sleep, depressive symptoms, and anxious symptoms (*p* < 0.05). - Greater engagement with the feature that promoted getting out of bed during restless nights was associated with greater improvements in ISI score (r = 0.37, *p* < 0.05).
Kuhn, E. et al. (2021) [124]	This pilot study found that the Insomnia Coach mobile app, a CBT-I-based self-management tool, is feasible, and acceptable, and shows promise for improving insomnia and related outcomes in veterans.	50	The Intervention was the Insomnia Coach mobile app, a free, CBT-I-based self-management app that participants used for 6 weeks.	- Insomnia severity (d = −1.1, *p* = 0.001). - Sleep-onset latency (*d* = −0.6, *p* = 0.021). - Global sleep quality (*d* = −0.9, *p* = 0.002). - Depression symptoms (*d* = −0.8, *p* = 0.012).
Kyle, S. et al. (2020) [125]	Digital cognitive behavioral therapy for insomnia decreases self-reported cognitive impairment but does not improve objective cognitive performance.	410	Digital cognitive behavioral therapy (dCBT) for insomnia.	- Reduction in self-reported cognitive impairment (BC-CCI score) at 10 weeks: −3.03 (95% CI: −3.60, −2.47, *p* < 0.0001, d = −0.86) in the dCBT group compared to control. - Maintained reduction in self-reported cognitive impairment at 24 weeks: d = −0.96 in the dCBT group compared to control. - Improvements in insomnia severity, sleep efficiency, cognitive failures, fatigue, sleepiness, depression, and anxiety in the dCBT group compared to control at both 10 and 24 weeks. - No significant differences between dCBT and control groups on objective tests of cognitive performance.
Liang, S. et al. (2022) [126]	Digital cognitive behavioral therapy for insomnia is an effective treatment for improving sleep quality in a real-world clinical setting.	6002	The intervention was digital cognitive behavior therapy for insomnia (dCBT-I) delivered through a mobile app to patients with insomnia, anxiety disorders, or comorbid insomnia and depression. The intervention was delivered for at least 8 weeks and, in some cases, up to 12 weeks.	- All three treatment groups (dCBT-I monotherapy, medication monotherapy, and combined dCBT-I and medication therapy) showed significant improvements in sleep quality (as measured by PSQI scores) from baseline to the 8-week follow-up. - In the depression group, those receiving combined dCBT-I and medication therapy showed further significant decreases in PSQI scores from the 8-week to the 12-week follow-up. - In the anxiety group, those receiving dCBT-I monotherapy or combined dCBT-I and medication therapy showed further significant decreases in PSQI scores from the 8-week to the 12-week follow-up. - There was a time-by-treatment interaction detected in the anxiety group, indicating the different treatment options had varying effects on PSQI score reduction over time.
Lovato, N. et al. (2014) [127]	A brief 4-week group-based cognitive behavioral therapy program improved sleep quality and daytime functioning in older adults with insomnia.	118	The intervention was a 4-week, group-based treatment program of cognitive behavior therapy for insomnia (CBT-I) that included bedtime restriction therapy, sleep education, and cognitive restructuring. The sessions were 60 min long and delivered weekly to small groups of 4-5 participants.	- Significant reductions in wake-after-sleep onset and increases in sleep efficiency, with large effect sizes that were maintained at 3-month follow-up. - Significant reductions in perceived insomnia severity that were maintained at 3-month follow-up. - Significant improvements in fatigue, daytime sleepiness, and daily functioning, with large effect sizes that were maintained for daily functioning at 3-month follow-up. - Significant improvements in sleep self-efficacy, dysfunctional beliefs about sleep, and sleep anticipatory anxiety, with large effect sizes that were maintained at 3-month follow-up.
Lu, M. et al. (2023) [128]	Digital cognitive behavioral therapy for insomnia is superior to medication therapy at 6-month follow-up, but the combination of the two is most effective long-term.	4052	Digital cognitive behavioral therapy for insomnia (dCBT-I). Medication therapy.Some participants received a combination of the two interventions.	- dCBT-I was superior to medication therapy at 6-month follow-up, although the results were unstable. - The combination of dCBT-I and medication resulted in a sustained improvement in sleep quality compared to monotherapy modalities.
Luik, A. et al. (2018) [129]	Using a wearable device to estimate sleep does not significantly affect the outcomes of digital cognitive behavioral therapy for insomnia.	3551	The intervention was digital cognitive behavioral therapy (dCBT) for insomnia.	- Significant improvements in insomnia symptoms (Cohen’s d = 1.45, *p* < 0.001) for all participants. - Significant improvements in depression, anxiety, perceived stress, life satisfaction, and work productivity for all participants. - Participants who did not use a wearable device had better sleep and less affected work productivity at baseline and post-treatment compared to those who did use a device, but the treatment effects were largely similar between the two groups. - Participants who used a wearable device engaged more with the additional components of the digital CBT program.
McCrae, C. et al. (2020) [130]	Telehealth delivery of cognitive behavioral therapy for insomnia in children with autism spectrum disorder is feasible and may improve sleep, behavior, and arousal.	17	The intervention was an 8-session telehealth cognitive behavioral treatment for childhood insomnia (telehealth CBT-CI) delivered to 17 children (ages 6–12) with autism spectrum disorder and insomnia, along with their parents. The treatment integrity was assessed for delivery, receipt/understanding, and enactment, and parents found the intervention to be helpful, age-appropriate, and autism-friendly.	- Improved child and parent sleep (objective and subjective). - Decreased child irritability, lethargy, stereotypy, and hyperactivity. - Decreased parent fatigue. - Decreased child inappropriate speech (at 1 month). - Reduced physiological arousal in most children following treatment.
McCurry, S. et al. (2021) [131]	Telephone-delivered cognitive behavioral therapy for insomnia improved sleep, fatigue, and pain in older adults with osteoarthritis.	327	Telephone-delivered cognitive behavioral therapy for insomnia (CBT-I), consisting of 6 sessions of 20–30 min each, delivered over 8 weeks. The CBT-I sessions included sleep restriction, stimulus control, sleep hygiene, cognitive restructuring, and homework.	- 2-month post-treatment: 8.1-point decrease in CBT-I group vs. 4.8-point decrease in control group, a significant 3.5-point greater decrease in the CBT-I group (*p* < 0.001). - 12-month follow-up: 3.0-point greater decrease in CBT-I group compared to control group (*p* < 0.001). - Remission (ISI ≤ 7) at 12-month follow-up: - 56.3% in CBT-I group vs. 25.8% in control group. - Fatigue: - 2-month post-treatment: 2.0-point greater reduction in CBT-I group compared to control group (*p* < 0.001). - 12-month follow-up: 1.8-point greater reduction in CBT-I group compared to control group (*p* = 0.003). - Pain: - Significant differences immediately post-treatment but not sustained at 12-month follow-up.
Miller, C. et al. (2023) [132]	Digital cognitive behavioral therapy was effective for reducing insomnia and fatigue symptoms in cancer patients.	70	The intervention was digital cognitive behavioral therapy (dCBT) for insomnia, delivered through the Sleepio digital therapeutic platform.	- Insomnia (SCI-8 score): dCBT group showed significantly greater reduction in insomnia symptoms compared to control at both post-treatment and 24-week follow-up (*p* < 0.05). - Fatigue (FFS score): dCBT group showed significantly greater reduction in fatigue symptoms compared to control at both post-treatment and 24-week follow-up (*p* < 0.05). - Global health-related quality of life (PROMIS-10 score): dCBT group showed non-significant improvement in global quality of life at post-treatment (*p* = 0.076) but significantly greater improvement compared to control at 24-week follow-up (*p* < 0.05).
Morin, C. et al. (2015) [133]	Cognitive behavioral therapy for insomnia is an effective, non-pharmacological treatment, but personalized AI-based approaches are not discussed.	1162	Cognitive behavioral therapy for insomnia (CBT-i).	- Significant improvements in sleep outcomes, including sleep-onset latency, wake-after-sleep onset, and sleep efficiency. - Improvements were well sustained over time, with no adverse outcomes. - Improved some aspects of daytime functioning, such as fatigue and distress. - However, the clinical significance of the sleep improvements in terms of patient well-being is unclear.
Morin, C. et al. (2023) [134]	Digital CBT-I treatment improved sleep and reduced anxiety and depression symptoms in adults with chronic insomnia.	991	The intervention is a 6–9-week prescription digital therapeutic (PDT) delivering cognitive behavioral therapy for insomnia (CBT-I), specifically the Somryst^®^ (previously SHUTi, Nox Health, Atlanta, GA, USA) program.	- Insomnia Severity Index (ISI) scores decreased from 18.8 at baseline to 11.3 post-treatment and 12.1 at 6-month follow-up (*p* < 0.0001). - Generalized Anxiety Disorder-7 (GAD-7) scores improved from baseline to post-treatment (Cohen’s d = 0.48) and 6-month follow-up (d = 0.45), both statistically significant (*p* < 0.0001). - Patient Health Questionnaire-8 (PHQ-8) scores for depression improved from baseline to post-treatment (d = 0.76, *p* < 0.001) and 6-month follow-up (d = 0.60, *p* < 0.0001). - The largest observed decreases in GAD-7 and PHQ-8 scores were among people with more severe baseline mood symptoms.
Neuenschwander, E. et al. (2023) [135]	Personalized light therapy is more effective than non-personalized light therapy in correcting circadian misalignment in night shift workers.	10	Personalized light schedules delivered through a mobile app based on estimates of each participant’s dim light melatonin onset (DLMO) derived from Apple Watch data. Provision of a light box to deliver bright light at night and light-blocking glasses.	- Personalized light therapy group: Mean phase delay of 7.37 h (SD = 3.03 h). - Non-personalized light therapy control group: Mean phase delay of 0.84 h (SD = 3.46 h). - The difference in phase delay between the two groups was statistically significant (*p* = 0.05).
Nguyen, S. et al. (2018) [136]	Cognitive behavioral therapy improves sleep quality over treatment as usual in persons with acquired brain injury, with better memory, younger age, and higher baseline depression predicting positive treatment response.	32	Cognitive behavior therapy (CBT) for sleep disturbance following acquired brain injury (ABI).	- CBT participants were 4.88 times more likely to achieve reliable improvements in sleep quality compared to the TAU group (*p* = 0.042). - Certain patient characteristics, including better verbal memory, younger age, and higher baseline depression, were associated with positive outcomes in the CBT group but did not significantly improve predictive ability over just looking at the study group (CBT vs. TAU) alone.
Okajima, I. et al. (2020) [137]	Tailored brief behavioral therapy for insomnia delivered via smartphone app improved insomnia severity, social disabilities, and work performance in workers.	92	All participants received a 2-week smartphone application intervention that assessed their sleep-related daily habits. Participants in the tailored BBTI group received a 2-week intervention involving 1–3 tailored challenge tasks based on their baseline assessment, covering techniques like stimulus control, sleep restriction, relaxation, and sleep hygiene. Participants in the standard BBTI group received a 2-week intervention involving a standard set of sleep hygiene tasks and articles about sleep science, sleep scheduling, and relaxation techniques.	- Insomnia severity (ISI): - Tailored BBTI group had significantly greater improvements than waiting list at 1-month (g = −0.85, *p* = 0.04) and 3-month (g = −1.01, *p* = 0.002) follow-ups. - Standard BBTI group had significantly greater improvements than waiting list at 3-month follow-up (g = −0.84, *p* = 0.009). - Social disabilities (SDISS): - Tailored BBTI group had significantly greater improvements in social life than self-monitoring (g = −1.14, *p* = 0.003) and waiting list (g = −1.33, *p* = 0.009) at 3-month follow-up. - Standard BBTI group had significantly greater improvements in social life than waiting list (g = −0.84, *p* = 0.009) at 3-month follow-up. - Tailored BBTI group had significantly greater improvements in family life than waiting list (g = −0.89, *p* = 0.005) at 3-month follow-up. - Sleep reactivity (FIRST): - Tailored BBTI group had significantly greater improvements than standard BBTI (g = −1.02, *p* = 0.004) and waiting list (g = −1.09, *p* = 0.007) at 3-month follow-up. - Work productivity (WLQ): - Tailored BBTI group had significantly greater improvements than standard BBTI (g = −0.89, *p* = 0.005) at 3-month follow-up.
Paniccia, G. et al. (2024) [138]	Personalized light therapy based on individual circadian rhythms is more effective than a one-size-fits-all approach for reducing insomnia and sleepiness in night shift workers.	21	The intervention was personalized light therapy, where participants received light therapy tailored to their individual circadian rhythms as determined by their dim light melatonin onset (DLMO). This was delivered through a mobile app (Arcashift) and a light box, along with light-blocking glasses.	- Insomnia symptoms: Personalized light therapy group decreased by 4.64 points on average, while the control group increased by 3.57 points on average. This difference was statistically significant (*p* < 0.05). - Peak sleepiness: Personalized light therapy group decreased by 0.21 points on average, while the control group increased by 0.77 points on average. This difference was also statistically significant (*p* < 0.001).
Patel, S. et al. (2016) [139]	Computerized cognitive behavioral therapy can be an effective treatment option for insomnia in Parkinson’s disease patients.	28	The intervention was a 6-week computerized cognitive behavioral therapy for insomnia (CCBT-I) program, which involved a series of daily lessons, homework assignments, learnable skills, and appropriate recommendations.	- Decreased significantly in both the CCBT-I treatment group (*p* < 0.002) and the control group (*p* < 0.008). - The decrease in ISI scores was significantly greater in the CCBT-I treatment group compared to the control group (*p* = 0.03). - Completion rates: - 8 out of 14 participants (57%) in the CCBT-I treatment group completed the study. - 13 out of 14 participants (93%) in the control group completed the study.
Peter, L. et al. (2019) [140]	Online and face-to-face CBT-I interventions are both effective in improving sleep efficiency in shift workers.	Total: 33	(1) A 4-week online cognitive behavioral therapy for insomnia (CBT-I) intervention delivered via 4 email contacts. (2) A 6-session face-to-face outpatient CBT-I treatment combined with bright light therapy and actigraphy.	- Improved sleep efficiency by an average of 7.18% over 4 weeks (*p* < 0.01). - Improved total sleep time by an average of 24.5 min per night over 4 weeks (*p* < 0.05). - Improved well-being (WHO-5 scores) and reduced insomnia symptoms (ISI scores), both *p* < 0.05. - No significant change in daytime sleepiness (ESS scores). - Face-to-face CBT-I intervention: - Improved sleep efficiency by an average of 7.58% over 4 weeks (*p* < 0.01). - No other quantitative effects reported for the face-to-face intervention.
Philip, P. et al. (2020) [141]	A smartphone-based virtual agent can provide personalized cognitive behavioral interventions for sleep concerns during COVID-19 confinement.	2069	Completing a 1-week sleep diary. Receiving 10 days of personalized sleep recommendations.	- After completing Step 1 (1 week of sleep diary): - Mean ISI score decreased from 18.56 to 15.99 (*p* < 0.001). - 36.7% of users had their ISI score decrease to below the clinical threshold for insomnia. - After completing Step 2 (10 days of personalized recommendations): - Mean ISI score decreased from 18.87 to 14.68 (*p* < 0.001). - 48.9% of users had their ISI score decrease to below the clinical threshold. - Improvements in sleep indicators including decreased WASO, NWAK, and TWAK and increased TIB, TST, and sleep efficiency.
Pulantara, I. et al. (2018) [142]	A just-in-time adaptive intervention app (iREST) delivered personalized, evidence-based sleep recommendations and showed promising results for improving sleep in a military population.	27	The intervention was the iREST mobile health platform, which included a mobile app for participants to use for 4–6 weeks and a clinician portal through which clinicians provided evidence-based behavioral sleep treatment recommendations to participants during weeks 2–4.	- Mean reduction on the Insomnia Severity Index (ISI): 9.96 (*p* < 0.001). - 70% of participants met criteria for treatment response. - 59% of participants achieved remission. - Response and remission rates were not significantly different from previous in-person insomnia treatment trials.
Reesen, J. et al. (2023) [143]	This protocol describes a study evaluating whether guided Internet-delivered cognitive behavioral therapy for insomnia improves sleep and affects emotional distress in people with mental health problems.	576	The intervention is a guided Internet-based cognitive behavioral therapy for insomnia (iCBT-I) called “i-Sleep”. It consists of 5 online sessions delivered over 5–8 weeks, which include psychoeducation on sleep, establishing healthy sleeping behaviors through bedtime restriction and stimulus control, and addressing cognitive factors associated with insomnia. Participants also complete daily sleep diary monitoring using an app.	- The expected effect size of the iCBT-I intervention on insomnia severity is a small effect (d = 0.177), which the study is powered to detect with 80% power at a significance level of 0.05. - The expected effect size of the iCBT-I intervention on sleep efficiency is even smaller (d = 0.126), which the study is powered to detect with 80% power at a significance level of 0.05. - The expected effect size of the iCBT-I intervention on the severity of each psychiatric symptom dimension (e.g., generalized anxiety, social anxiety, and PTSD) is small (d = 0.216), which the study is powered to detect with 80% power at a significance level of 0.01. - The expected effect size of the iCBT-I intervention on the severity of each psychiatric symptom dimension in participants who receive regular mental health treatment between the 2-month and 8-month follow-ups is small to medium (d = 0.305), which the study is powered to detect with 80% power at a significance level of 0.01.
Reilly, E. et al. (2021) [144]	The use of a mobile app-delivered cognitive behavioral therapy for insomnia improves sleep outcomes in veterans, including those with comorbid conditions.	33	CBT-i Coach app: A mobile app that provides cognitive behavioral therapy for insomnia (CBTI) content and tools, used for 6 weeks. CBT-i Coach app plus a physical activity (PA) intervention: The CBT-i Coach app plus an additional PA intervention, used for 6 weeks.	- Insomnia: Significant improvement (*p* < 0.001). - Sleep quality: Significant improvement (*p* < 0.001). - Functional sleep outcomes: Significant improvement (*p* = 0.002). - Objective sleep efficiency: Significant but modest increase (*p* = 0.02).
Ren, R. et al. (2023) [145]	Integrating personalized telephone sessions into digital cognitive behavioral therapy for insomnia provides increased clinical benefit, particularly for discontinuing sleep medications.	572	D-CBTI: Fully automated, interactive CBTI delivered through a mobile app over 6 weekly sessions. TD-CBTI: D-CBTI plus weekly 10–15 min personalized telephone sessions.	- Odds of sleep-efficiency-based remission: 167% higher in TD-CBTI group compared to D-CBTI group (OR = 2.67, 95% CI 1.34–5.23). - Odds of reduction in sleep medication use: 352% higher in TD-CBTI group compared to D-CBTI group (OR = 4.52, 95% CI 1.27–10.05).
Ritterband, L. et al. (2017) [146]	A web-based cognitive behavioral therapy for insomnia intervention showed long-term effectiveness in improving sleep outcomes.	303	The intervention was a 6-week, fully automated, interactive, and tailored web-based cognitive behavior therapy for insomnia (CBT-I) program called “Sleep Healthy Using the Internet (SHUTi)”. It incorporated the primary tenets of face-to-face CBT-I and was compared to an online patient education program.	- Insomnia Severity Index: Significant group x time interaction favoring SHUTi (*p* < 0.001), with large within-group effect sizes (Cohen’s d = 0.79 to 1.90) and maintained effects at 1-year follow-up (d = 2.32). - Sleep-onset latency: Significant group x time interaction favoring SHUTi (*p* < 0.001), with large within-group effect sizes (d = 0.79 to 1.41) and maintained effects at 1-year follow-up (d = 1.41). - Wake-after-sleep onset: Significant group x time interaction favoring SHUTi (*p* < 0.001), with large within-group effect sizes (d = 0.95 to 1.90) and maintained effects at 1-year follow-up (d = 0.95). - 56.6% of SHUTi participants achieved remission status, and 69.7% were deemed treatment responders at 1-year follow-up. - Secondary sleep outcomes (except total sleep time) also showed significant improvements in the SHUTi group compared to control.
Rötger, A. et al. (2024) [147]	Digital cognitive behavioral therapy for insomnia reduces insomnia and comorbid symptoms of depression and anxiety.	238	8 weeks of digital cognitive behavioral therapy for insomnia.	- Depression subgroup: d = 2.37 (large reduction compared to control). - Anxiety subgroup: d = 2.13 (large reduction compared to control). - For depressive symptoms in the depression subgroup: d = 1.59 (between-group treatment effect). - For anxiety symptoms in the anxiety subgroup: d = 1.28 (between-group treatment effect). - Within-group effects were stable over time, with effect sizes ranging from d = 0.64 to 1.63.
Sadler, P. et al. (2018) [148]	Cognitive behavioral therapy for insomnia, with or without positive mood strategies, effectively reduced insomnia and depression in older adults, but the advanced form was not significantly superior.	72	Cognitive behavior therapy for insomnia (CBT-I, standard). Cognitive behavior therapy for insomnia plus positive mood strategies (CBT-I+, advanced).Both interventions were delivered in an 8-week group therapy format.	- CBT-I and CBT-I+ both led to significantly greater reductions in insomnia and depression severity compared to the psychoeducation control group (*p* < 0.001), and these effects were maintained at 20-week follow-up. - The differences in outcomes between the CBT-I and CBT-I+ interventions were not statistically significant, but the study was not powered to determine if one was superior or if the two were equivalent.
Schuffelen, J. et al. (2023) [149]	Digital cognitive behavioral therapy for insomnia is effective in reducing insomnia symptoms and improving daytime functioning in a heterogeneous study sample.	238	The intervention in this study was digital cognitive behavioral therapy for insomnia (dCBT-I) delivered through the “somnio” platform from the company mementor DE GmbH. Participants in the intervention group received access to the dCBT-I intervention over an 8-week period.	- Large reductions in insomnia severity (d = −2.08). - Higher responder rates (63.6% vs. 5.8%) and remission rates (41.5% vs. 3.3%) for insomnia. - Large reductions in fatigue (d = −1.02) and dysfunctional beliefs about sleep (d = −1.17). - Small-to-medium improvements in daytime sleepiness (d = −0.26), well-being (d = 0.68), physical quality of life (d = 0.42), depressive symptoms (d = −0.80), and anxiety symptoms (d = −0.56). - No significant differences were found between groups for dream recall frequency or nightmare frequency.
Schwartz, B. et al. (2020) [150]	This paper describes a treatment selection algorithm using machine learning and statistical inference to recommend cognitive behavioral or psychodynamic therapy for individual patients.	1379	The interventions were cognitive behavioral therapy (CBT) and psychodynamic therapy. The abstract does not provide any details on the frequency, duration, or amount/dose of these interventions.	- For the full sample, the difference in outcomes between patients treated with their optimal vs. non-optimal treatment was not statistically significant in the holdout data (b = −0.043, *p* = 0.280). - However, for the 50% of patients with the largest predicted benefit of receiving their optimal treatment, the average percentage of change on the BSI was 52.6% for their optimal treatment and 38.4% for their non-optimal treatment, which was a statistically significant difference (*p* = 0.017, d = 0.33).
Selvanathan, J. et al. (2021) [151]	Cognitive behavioral therapy for insomnia improves sleep, pain, and depressive symptoms in patients with chronic non-cancer pain.	762	The intervention was cognitive behavioral therapy for insomnia (CBT-I) for patients with comorbid insomnia and chronic non-cancer pain.	- Post-treatment: Standardized mean difference of 0.89 (significant). - Follow-up: Standardized mean difference of 0.56 (significant). - Probability of better sleep: 81% at post-treatment, 71% at follow-up. - Pain: - Post-treatment: Standardized mean difference of 0.20 (significant). - Probability of less pain: 58% at post-treatment, 57% at follow-up. - Depressive symptoms: - Post-treatment: Standardized mean difference of 0.44 (significant). - No statistically significant effects on anxiety symptoms or fatigue.
Siengsukon, C. et al. (2020) [152]	Web-based cognitive behavioral therapy for insomnia is feasible and effective for improving sleep outcomes in individuals with multiple sclerosis.	41	6-week web-based cognitive behavioral therapy for insomnia (wCBT-I) program. Biweekly (twice per week) support telephone calls, provided to one group in addition to the web-based CBT-I program.	- Significant improvements in insomnia severity, sleep quality, sleep self-efficacy, and anxiety in both the wCBT-I group and the wCBT-I + calls group. - Significant improvements in depression and fatigue in the wCBT-I group only.
Speed, T. et al. (2022) [153]	This paper evaluates the feasibility of implementing cognitive behavioral therapy for insomnia in an outpatient substance use disorder treatment program but does not address personalized AI-based CBT-I.	21	Group-based cognitive behavioral therapy for insomnia (gCBT-I). Standard of care (SOC) for the outpatient SUD treatment program embedded within the therapeutic community.	- 80% of participants in the gCBT-I group achieved an Insomnia Severity Index (ISI) score of ≤ 8 (mild insomnia) compared to 25% in the SOC group.
Stott, R. et al. (2021) [154]	Offering a digital CBT-based sleep intervention alongside routine mental health treatment in an IAPT service improved clinical outcomes.	510	The intervention was a self-guided digital sleep intervention called Sleepio, which is based on the principles of cognitive behavioral therapy for insomnia (CBT-I).	- Significantly better outcomes on PHQ-9 (depression), GAD-7 (anxiety), and WSAS (work and social adjustment) for the Sleepio group compared to controls. - Higher IAPT recovery rates (on PHQ-9 and GAD-7) for the Sleepio group (64.7%) compared to controls (58%). - Marginally elevated clinical contact time (less than 1 h) for the Sleepio group compared to controls.
Sweetman, A. et al. (2017) [155]	Cognitive behavioral therapy for insomnia is an effective treatment for insomnia in patients with comorbid obstructive sleep apnea.	455	Cognitive behavioral therapy for insomnia (CBT-i).	- Significant improvements in insomnia symptoms, global insomnia severity, and daytime functioning measures in patients with comorbid insomnia and obstructive sleep apnea (OSA). - The effectiveness of CBT-i was not impaired by the presence of comorbid OSA as the improvements were similar between patients with and without OSA. - The presence and severity of OSA did not affect the rates of insomnia remission or treatment resistance following the CBT-i intervention.
Sweetman, A. et al. (2020) [156]	Cognitive behavioral therapy for insomnia is safe and effective in patients with co-morbid moderate and severe sleep apnea, though they should be monitored for increased daytime sleepiness during initial bedtime restriction.	145	The intervention was a 4-week cognitive and behavioral therapy for insomnia (CBTi) program delivered by psychologists, with each session lasting 45 min and occurring either individually or in small groups. The key components of the CBTi program included sleep education, sleep hygiene, bedtime restriction therapy (BRT), feedback on sleep study results, cognitive therapy, and relapse prevention. BRT was initiated in the first week by restricting time in bed to match the patient’s average pretreatment total sleep time, with a minimum of 5.5 h. Psychologists reviewed the patients’ sleep diaries, sleepiness scores, and verbal reports each week to inform decisions about continuing, modifying, or discontinuing the BRT.	- Greater reduction in time in bed from pre- to post-treatment (75.8 min reduction in CBTi vs. 14.5 min in control, *p* < 0.001). - Greater improvement in sleep efficiency (significant interaction effect, *p* < 0.001) and wake-after-sleep onset (significant interaction effect, *p* = 0.016) by post-treatment. - Greater increase in sleep efficiency from pre- to post-treatment among patients with lower pretreatment daytime sleepiness (significant 3-way interaction, *p* = 0.013).
Talbot, L. et al. (2014) [157]	Cognitive behavioral therapy for insomnia improves sleep in individuals with post-traumatic stress disorder.	45	The intervention was an 8-session weekly individual cognitive behavioral therapy for insomnia (CBT-I) delivered by a licensed clinical psychologist or a board-certified psychiatrist. The key components of the CBT-I intervention were stimulus control therapy and sleep restriction therapy.	- Reduced sleep-onset latency (SOL) and wake-after-sleep onset (WASO). - Increased sleep efficiency (SE) and total sleep time (TST). - Polysomnography outcome with significant improvement in CBT-I vs. waitlist control: - Increased total sleep time (TST). - Self-report sleep outcomes with significant improvements in CBT-I vs. waitlist control: - Reduced Insomnia Severity Index (ISI) scores. - Improved Pittsburgh Sleep Quality Index (PSQI) and Epworth Sleepiness Scale (ESS) scores. - Disruptive nocturnal behaviors outcome with significant improvement in CBT-I vs. waitlist control: - Reduced Pittsburgh Sleep Quality Index-Addendum (PSQI-A) scores. - Psychosocial functioning outcome with significant improvement in CBT-I vs. waitlist control: - Reduced Work and Social Adjustment Scale (WSAS) scores.
Tomfohr-Madsen, L. et al. (2017) [158]	Group cognitive behavioral therapy for insomnia delivered during pregnancy was associated with improvements in sleep and mood.	13	Group cognitive behavioral therapy for insomnia (CBT-I) delivered over 5 weekly sessions to pregnant women with insomnia.	- Insomnia symptoms: Significant reduction. - Subjective sleep quality: Significant increase. - Time in bed (TIB): Decreased. - Sleep-onset latency (SOL): Decreased. - Sleep efficiency (SE): Increased. - Subjective total sleep time (TST): Increased. - Symptoms of depression: Decreased. - Pregnancy-specific anxiety: Decreased. - Fatigue: Decreased. - All changes were statistically significant with medium to large effect sizes.
Tomfohr-Madsen, L. et al. (2020) [159]	This pilot study found that 6 weeks of cognitive behavioral therapy for insomnia improved sleep in adolescents with persistent post-concussion symptoms.	24	The intervention was 6 weeks of cognitive behavioral therapy for insomnia (CBT-I).	- Insomnia Severity Index: Large and clinically significant improvements in the CBT-I group compared to treatment-as-usual control, maintained at follow-up. - Sleep quality (Pittsburgh Sleep Quality Index): Improved in CBT-I group compared to control. - Dysfunctional beliefs about sleep (Dysfunctional Beliefs and Attitudes about Sleep Scale): Fewer dysfunctional beliefs in CBT-I group compared to control. - Sleep efficiency, sleep-onset latency, and total sleep time (7-night sleep diary): Improved in CBT-I group compared to control. - Post-concussion symptoms (Health and Behavior Inventory): Modest reduction in CBT-I group compared to control.
Trauer, J. et al. (2015) [160]	Cognitive behavioral therapy is an effective nonpharmacologic treatment for chronic insomnia.	1162	The intervention was cognitive behavioral therapy for insomnia (CBT-i) that incorporated at least 2 of the following 5 components: cognitive therapy, stimulus control, sleep restriction, sleep hygiene, and relaxation therapy. The CBT-i was delivered in person on at least 2 occasions, but the frequency, duration, and amount/dose of the intervention are not specified.	- Immediately after treatment: CBT-i reduced SOL compared to control (*p* < 0.001). - Early follow-up: CBT-i reduced SOL compared to control (*p* < 0.001). - Late follow-up: CBT-i reduced SOL compared to control (*p* < 0.001). - Wake-after-sleep onset (WASO): - Immediately after treatment: CBT-i reduced WASO compared to control (*p* < 0.001). - Early follow-up: CBT-i reduced WASO compared to control (*p* < 0.001). - Late follow-up: CBT-i reduced WASO compared to control (*p* < 0.001). - Total sleep time (TST): - Immediately after treatment: CBT-i increased TST compared to control (*p* < 0.001). - Early follow-up: CBT-i increased TST compared to control (*p* < 0.001). - Late follow-up: CBT-i increased TST compared to control (*p* < 0.001). - Sleep efficiency (SE%): - Immediately after treatment: CBT-i increased SE% compared to control (*p* < 0.001). - Early follow-up: CBT-i increased SE% compared to control (*p* < 0.001). - Late follow-up: CBT-i increased SE% compared to control (*p* < 0.001).
Watanabe, Y. et al. (2022) [161]	Smartphone-based cognitive behavioral therapy app is effective for treating insomnia.	175	Smartphone-based cognitive behavioral therapy for insomnia (CBT-I) app.	- Change in Athens Insomnia Score (AIS) from baseline: - Active group: −6.7 ± 4.4. - Sham group: −3.3 ± 4.0. - Difference between groups: −3.4 (*p* < 0.001), indicating a significantly greater reduction in the Active group. - Change in Clinical Global Impression of Improvement (CGI-I) from baseline: - Active group: 1.3 ± 0.8. - Sham group: 0.7 ± 0.8. - Difference between groups: *p* < 0.001, indicating significantly greater improvement in the Active group. - Proportion of patients with AIS less than 6 (remission): - Active group: 37.9%. - Sham group: 10.2%. - Difference between groups: *p* < 0.001, indicating a significantly higher remission rate in the Active group. - No adverse reactions or device failures were detected in the Active group.
Zachariae, R. et al. (2016) [162]	Internet-delivered cognitive behavioral therapy for insomnia is efficacious and can be considered a viable treatment option.	1460	The intervention was Internet-delivered cognitive behavioral therapy for insomnia (eCBT-I).	- Improved insomnia severity (effect size range: 0.21 to 1.09). - Improved sleep efficiency (effect size range: 0.21 to 1.09). - Improved subjective sleep quality (effect size range: 0.21 to 1.09). - Reduced wake-after-sleep onset (effect size range: 0.21 to 1.09). - Reduced sleep-onset latency (effect size range: 0.21 to 1.09). - Increased total sleep time (effect size range: 0.21 to 1.09). - Reduced number of nocturnal awakenings (effect size range: 0.21 to 1.09). The effects were comparable to in-person CBT-I and were generally maintained at 4–48-week follow-up. Longer treatment duration, more personal clinical support, and lower dropout rates were associated with larger intervention effects.
Zhang, C. et al. (2023) [163]	A smartphone-based, Chinese culture-adapted digital cognitive behavioral therapy for insomnia app reduced insomnia severity in the Chinese cultural context.	82	The intervention was a smartphone-based digital cognitive behavioral therapy for insomnia (DCBT-I) app that was tailored to the Chinese cultural context. Participants in the DCBT-I group used the app for approximately 10–15 min per day, with the app using a question-and-answer format to simulate a consultation process. The DCBT-I app included local language, Chinese cultural activities, and content consistent with Chinese lifestyles and cognition.	- Both DCBT-I and sleep education groups had significant improvements, with large effect sizes (DCBT-I: d = 1.50 to 1.71, sleep education: d = 1.02 to 1.22). - 65.8% of DCBT-I participants were ISI responders (≥8-point reduction) at intervention completion, compared to 30.8% in the sleep education group. - 63.2% of DCBT-I participants were ISI responders at 3-month follow-up, compared to 35.9% in the sleep education group. - Mental health outcomes: - The DCBT-I group showed greater improvements in mental component scores of SF-12, PHQ-9 (depression), and GAD-7 (anxiety) at 3-month follow-up compared to the sleep education group. - Objective sleep measures: - The DCBT-I group had significantly higher total sleep time recorded by smart bracelet at 3-month follow-up compared to the sleep education group (448.1 vs. 424.5 min).

## Data Availability

Not applicable.

## Abbreviations

AI	Artificial Intelligence
CBT	Cognitive Behavioral Therapy
CBT-I	Cognitive Behavioral Therapy for Insomnia
RCT	Randomized Controlled Trial
PSQI	Pittsburgh Sleep Quality Index
ISI	Insomnia Severity Index
WASO	Wake-After-Sleep Onset
SOL	Sleep-Onset Latency
SE	Sleep Efficiency
dCBT	Digital Cognitive Behavioral Therapy
TAU	Treatment as Usual
SCI	Sleep Condition Indicator
CES-D	Center for Epidemiological Studies Depression
HADS	Hospital Anxiety and Depression Scale
GHQ	General Health Questionnaire
ADSM	Active-Duty Service Members
CDS	Clinical Decision Support
IoT	Internet of Things
SH	Sleep Hygiene
CBT-P	Cognitive Behavioral Therapy for Pain
CBT-IP	Cognitive Behavioral Therapy for Insomnia and Pain
dCBTI	Digital Cognitive Behavioral Therapy for Insomnia
PD	Placebo Drug
AD	Antidepressant
HAMD17	Hamilton Depression Rating Scale (17 items)
BC-CCI	Beliefs and Concerns About Cognitive Impairment
SMD	Standardized Mean Difference
CrI	Credible Interval
QIDS-SR	Quick Inventory of Depressive Symptomatolog—Self-Report
PHQ-4	Patient Health Questionnaire-4
DBAS-16	Dysfunctional Beliefs and Attitudes About Sleep (16 items)
IRT	Imagery Relief Therapy
